# Yanghe Decoction Suppresses the Experimental Autoimmune Thyroiditis in Rats by Improving NLRP3 Inflammasome and Immune Dysregulation

**DOI:** 10.3389/fphar.2021.645354

**Published:** 2021-06-21

**Authors:** Bing’e Ma, Dexuan Chen, Yangjing Liu, Zhengping Zhao, Jianhua Wang, Guowei Zhou, Kun Xu, Taiyang Zhu, Qiong Wang, Chaoqun Ma

**Affiliations:** ^1^ Department of General Surgery, Affiliated Hospital of Nanjing University of Chinese Medicine, Jiangsu Province Hospital of Chinese Medicine, Nanjing, China; ^2^ Department of Thyroid and Breast Surgery, Affiliated Hospital of Integrated Traditional Chinese and Western Medicine for Nanjing University of Chinese Medicine, Jiangsu, China; ^3^ Department of Pharmacology, Affiliated Hospital of Nanjing University of Chinese Medicine, Jiangsu Province Hospital of Chinese Medicine, Nanjing, China

**Keywords:** autoimmune thyroiditis, Yanghe decoction, IL-35, Th17/Treg, Wnt/β-catenin, NLRP3 inflammasome

## Abstract

Inflammation is an important contributor to autoimmune thyroiditis. Yanghe decoction (YH) is a traditional Chinese herbal formulation which has various anti-inflammatory effects. It has been used for the treatment of autoimmune diseases such as ankylosing spondylitis In this study we aimed to investigate the effects of YH on autoimmune thyroiditis *in a rat model* and elucidate the underlying mechanisms. The experimental autoimmune thyroiditis (EAT) model was established by thyroglobulin (pTG) injections and excessive iodine intake. Thyroid lesions were observed using hematoxylin and eosin (H and E) staining and serum TgAb, TPOAb, TSH, T3, and T4 levels were measured by enzyme-linked immunosorbent assay IL-35 levels were evaluated using real-time polymerase chain reaction (RT-PCR) and Th17/Treg balance in peripheral blood mononuclear cells (PBMCs) was determined by flow cytometry and RT-PCR. Changes in Wnt/β-catenin signaling were evaluated using Western blot. Immunofluorescence staining and western blot were employed to examine NLRP3 inflammasome activation in the thyroid. YH minimized thyroid follicle injury and decreased concentrations of serum TgAb, TPOAb, TSH, T3, and T4 in EAT model. The mRNA of IL-35 was increased after YH treatment. YH also increased the percentage of Treg cells, and decreased Th17 proportion as well as Th17/Treg ratio in PBMCs. Meanwhile, the mRNA levels of Th17 related cytokines (RORγt, IL-17A, IL-21, and IL-22) were suppressed and Treg related cytokines (FoxP3, TGF-β, and IL-10) were promoted in PBMCs. Additionally, the protein expressions of Wnt-1 and β-catenin were unregulated after YH treatment. NLRP3 immunostaining signal and protein levels of IL-17, p-NF-κB, NLRP3, ASC, cleaved-Caspase-1, cleaved-IL-1β, and IL-18 were downregulated in the thyroid after YH intervention. Overall, the present study demonstrated that YH alleviated autoimmune thyroiditis in rats by improving NLRP3 inflammasome and immune dysregulation.

## Introduction

Autoimmune thyroiditis (AIT), the main cause of acquired hypothyroidism, constitutes 30% of all the auto-aggressive diseases. Autoimmune thyroiditis is characterized by lymphocytic infiltration eventually leads to the destruction of the thyroid follicles and the presence of autoantibodies against various thyroid antigens, particularly thyroid peroxidase (TPOAb) and thyroglobulin (TgAb). In clinical practice, the treatment of AIT predominantly depends on nonspecific anti-thyroid drugs, immunosuppressant and anti-inflammatory agents. However, disadvantages such as insufficient efficacy and obvious side effects limit the clinical usages of these drugs and novel therapies are urgently needed (Ma et al., 2017).

Traditional Chinese Medicine (TCM) seems to be an alternative treatment for AIT in China considering its efficacy, low adverse effects and costs. Yanghe decoction (YH) has been recorded in the *Life-saving Manual of Diagnosis and Treatment of External Diseases from the Qing dynasty.* It is able to dissipate cold and dispel dampness according to basic theory of TCM. It has been reported that modified YH can be used for ankylosing spondylitis (AS) treatment (Wang and Xiao 2018). Interestingly, the rate of autoimmune thyroid disease, specifically Hashimoto’s thyroiditis, is significantly higher in patients with ankylosing spondylitis than the healthy control group. We speculated that YH might protect against autoimmune thyroiditis. Therefore, a rat EAT model was established to investigate the effect of YH on autoimmune thyroiditis.

YH contains seven herbal medicines including Radix Rehmanniae praeparata [Scrophulariaceae; Rehmanniae Radix praeparata], Cortex Cinnamomi [Lauraceae; Cinnamomi Cortex], Ephedra sinica stapf [Ephedreae; Ephedrae Herba], Semen brassicae [Cruciferae; Sinapis Semen], Zingiber offcinale Rose [Zingiberaceae; Zingiberis Rhizome], Radix Rhizoma glycyrrhizae [Leguminosae; Glycyrrhizae radix et rhizoma], Colla cornus cervi [Cervidae; Cervi cornus colla]. Various anti-pharmacological activities have been detected in the components of Yanghe decoction. Radix glycyrrhizae extract can suppress NF-κB-mediated NLRP3 inflammasome activation on intracerebral hemorrhage model (Zeng et al., 2017). Semen brassicae attenuated the thoracic aortic remodeling, inflammation, and oxidative damage in spontaneously hypertensive rats via the downregulation of NF-κB and IL-1β (Lin et al., 2020). The oral administration of Cinnamomum cassia powder protected mice from experimental allergic encephalomyelitis by promoting the production of Foxp3+ T cells, and increasing the mRNA levels of Foxp3 and CD25 (Mondal and Pahan, 2015). Moreover, Cinnamomum cassia was able to increase IL-35, Foxp3, TGF-β, IL-10 mRNA expression in the spleen and thymus of rats with heat syndrome (Wu et al., 2019). These findings indicate that the effects of YH in the rat with experimental autoimmune thyroiditis (EAT) may be related to immune-inflammatory signaling.

The balance between Th17 cells and regulatory T cells (Tregs) has emerged as a prominent factor in regulating autoimmunity. Th17 triggers the immune response via the release of inflammatory cytokines such as IL-17A, IL-21, IL-22, while Treg inhibits the immune response via secreting immunosuppressive cytokines including IL-10 and TGF-β. The Th17/Treg imbalance was identified in several subtypes of autoimmune thyroid diseases such as Hashimoto’s thyroiditis, Graves’ disease and Graves’ ophthalmopathy (Li et al., 2016). Therapies exerted protective efficacy in EAT models by regulating Th17/Treg cell balance (Hou et al., 2018).

Recently, NLRP3 has been reported as a new negative regulator of Treg differentiation (Park et al., 2019). The NLRP3 inflammasome is a large intracellular multiprotein complex, which consists of the sensor molecule NLRP3, the adaptor protein ASC, and the precursor pro-Caspase-1. NLRP3 inflammasome activation leads to the maturation of Caspase-1, which further triggers the release of proinflammatory cytokines IL-1β and IL-18 (Wan et al., 2016). The NLRP3 inflammasome has been demonstrated to be essential for the pathogenesis and development of AIT. Upregulated mRNA and protein expressions of NLRP3, ASC, Caspase-1, pro-IL-1β, and pro-IL-18 were observed in thyroid tissues from AIT patients (Guo et al., 2018). Excessive iodine intake contributed to the development of AIT via the NLRP3 inflammasome activation in thyroid follicular cells (Liu et al., 2019).

The present study was aimed to investigate the effect of YH in a rat EAT model and to elucidate the immune-inflammatory mechanism. The EAT model was established by thyroglobulin (pTG) injections accompanied by excessive iodine intake. We demonstrated that YH intervention could mitigate thyroid follicle injury and reduce the concentration of TgAb, TPOAb, TSH, T3, and T4 in the serum. Furthermore, we also found that YH treatment could suppress the EAT by improving NLRP3 inflammasome and immune dysregulation.

## Materials and Methods

### Reagents

Selenious Yeast Tablet (Selenious) was obtained from Mudanjiang Lingtai Pharmaceuticals CO., Ltd. (Mudanjiang, China). Porcine thyroglobulin (pTg), complete Freund’s adjuvant (CFA) and incomplete Freund’s adjuvant (IFA) were provided by Sigma Aldrich (CA, United States). Lugol’s iodine solution was supplied by Preparation Room of Jiangsu Traditional Chinese and Western Medicine Hospital (Nanjing, China). The antibodies were purchased from CST (Danvers, United States) and Abcam (Burlingame, United States). Antibodies to Wnt-1 (AF5315, Affinity), β-catenin (8,480, CST), IL-17A (DF6127, Affinity), p-NF-κB p65 (3,039, CST), NF-κB p65 (8,242, CST), NLRP3 (ab214185, Abcam), ASC (DF6304, Affinity), Cleaved-Caspase1 (AF4022, Affinity), Cleaved-IL-1β (AF5103, Affinity), IL-18 (ab191860, Abcam), β-Tubulin (AF7011, Affinity) and HRP-conjugated secondary antibodies (7,074, CST) were used for western blot. NLRP3 (DF7438, Affinity) and Alexa Fluor® 488 secondary antibodies (62,304, CST) were used for the immunofluorescent detection of NLRP3.

### Extract Preparation and Quality Control of YH Extract

YH consists of seven herbal medicines including Radix Rehmanniae praeparata [Scrophulariaceae; Rehmanniae Radix praeparata] (30 G, batch No. 19010101), Cortex Cinnamomi [Lauraceae; Cinnamomi Cortex] (3 G, batch No. 190706), Ephedra sinica stapf [Ephedreae; Ephedrae Herba] (2 G, batch No. 190812), Semen brassicae [Cruciferae; Sinapis Semen] (6 G, batch No. 190609), Zingiber offcinale Rose [Zingiberaceae; Zingiberis Rhizome] (2 G, batch No. 1908015), Radix Rhizoma glycyrrhizae [Leguminosae; Glycyrrhizae radix et rhizoma] (3 G, batch No. 190728), and Colla cornus cervi [Cervidae; Cervi cornus colla] (9 G, batch No. 1906002). To obtain the water-soluble extract, 0.5 kG YH powder was soaked in water for 1 h and heated at 110°C for another hour. After filtration, YH fluid extract was evaporated into dried extract powder under vacuum of 60°C. The dried powder was dissolved in distilled water for further experiments.

**FIGURE 1:**
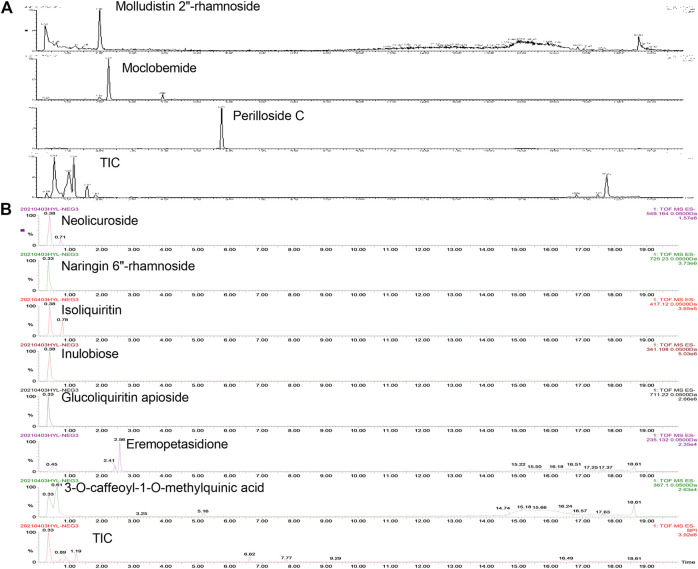
Representative extracted ion and total ion currents (TIC) chromatograms of the major constituents in YH decoction. The compounds are detected under positive (**A** [M + H]^+^) and negative (**B** [M-H]^−^) mode, respectively.

For the quantitative determination of compounds, 50 mG of dried YH extraction was dissolved in 1 ml of distilled water and filtered through a microporous membrane (0.22 μM) as the testing sample. Sample was detected using QTOF-MS (Xevo G2-XS, Waters) operating in positive ion mode. LC separation was achieved on an BEH C18 column using a gradient of solve A (10 mM ammonium acetate) and solvent B (acetonitrile). The flow rate was 0.4 ml/min. The gradient was: 0 min, 20% B; 1.5 min, 20% B; 16 min, 99% B; 18 min, 99% B; 18.1 min, 20% B; 20 min, 20% B. The mass spectrometer was operated with the spray voltage of 3 kV in negative mode. Cone gas and desolvation gas were set at 500 and 800 L/h, respectively. The source temperature was 120°C. Fast data-dependent acquisition (DDA) MS/MS experiments were performed with collision energy map which included low mass ramp start 5 eV from 10 eV and high mass ramp start 40 eV from 65 eV CE. The Progenesis QI (Nonlinear Dynamics, Newcastle, United Kingdom) was used for peak picking and alignment to screen the compounds in sample. Molecular identification was accomplished by matching the acquired precursors and fragment ions against several standard metabolome databases including the Human Metabolome database (http://www.hmdb.ca/), MassBank (http://www.massbank.jp/index.html). The representative extracted ions and total ion currents (TIC) chromatograms of the major constituents in YH decoction are shown in Figure 1. The main components are summarized in Table 1.

**TABLE 1:** Main components in YH decoction.

Compound	m/z	Retention time (min)	Accepted description	Adducts	Formula
17.05_547.4119 m/z	547.4119	17.04642	2-Hexaprenyl-6-methoxy-1,4-benzoquinone	M + H	C_37_H_54_O_3_
5.75_145.0990 m/z	145.0990	5.749,917	4-Amino-1-piperidinecarboxylic acid	M + H	C_6_H_12_N_2_O_2_
2.88_371.2028 m/z	371.2028	2.878,917	5-Megastigmen-7-yne-3,9-diol 3-glucoside	M + H	C_19_H_30_O_7_
2.93_401.1755 m/z	401.1755	2.9278	Corchoionoside B	M + H	C_19_H_28_O_9_
4.39_265.1254 m/z	265.1254	4.3867	Ilicifolinoside A	M + H	C_11_H_20_O_7_
2.27_269.1085 m/z	269.1085	2.265,733	Moclobemide	M + H	C_13_H_17_ClN_2_O_2_
1.98_563.1736 m/z	563.1736	1.9841	Molludistin 2″-rhamnoside	M + H	C_27_H_30_O_13_
5.75_317.1970 m/z	317.1970	5.749,917	Perilloside C	M + H	C_16_H_28_O_6_
5.30_233.0742 m/z	233.0742	5.303,217	Sampangine	M + H	C_15_H_8_N_2_O
18.13_279.1216 m/z	279.1216	18.12807	Solanolone	M + H	C_15_H_18_O_5_
1.47_249.2249 m/z	249.2249	1.473,567	Sterol	M + H	C_17_H_28_O
16.51_325.1830 m/z	325.1830	16.51483	2-Dodecylbenzenesulfonic acid	M-H	C_18_H_30_O_3_S
4.03_331.0807 m/z	331.0807	4.032383	3,4′,5-trihydroxy-3′,7-dimethoxyflavanone	M-H	C_17_H_16_O_7_
0.38_367.1057 m/z	367.1057	0.37775	3-O-Caffeoyl-1-O-methylquinic acid	M-H	C_17_H_20_O_9_
2.93_207.1374 m/z	207.1374	2.9307	Carvyl propionate	M-H	C_13_H_20_O_2_
2.56_235.1322 m/z	235.1322	2.56145	Eremopetasidione	M-H	C_14_H_20_O_3_
0.33_711.2208 m/z	711.2208	0.33155	Glucoliquiritin apioside	M-H	C_32_H_40_O_18_
0.38_341.1080 m/z	341.1080	0.37775	Inulobiose	M-H	C_12_H_22_O_11_
0.38_417.1195 m/z	417.1195	0.37775	Isoliquiritin	M-H	C_21_H_22_O_9_
0.33_725.2362 m/z	725.2362	0.33155	Naringin 6″-rhamnoside	M-H	C_33_H_42_O_18_
0.38_549.1650 m/z	549.1650	0.37775	Neolicuroside	M-H	C_26_H_30_O_13_

### Animals

Female Sprague-Dawley rats, weighing 220–240 G, were purchased from Beijing Vital River Laboratory Animal Technology CO., Ltd. (Beijing, China). Rats were housed under specific pathogen-free conditions with ad libitum access to food and water. All experiments were performed in accordance with the Guide for the Care and Use of Laboratory Animals approved by the Institutional Animal Care and Use Committee at Nanjing University of Chinese medicine.

### Experimental Design

After acclimation, rats were randomized into five groups: Control, Model, YH (5 g crude drug/kG), YH (15 g crude drug/kG), and Selenious (300 μg/kG). The induction of EAT was performed as previously described with minor modifications (Cui et al., 2014). Briefly, rats in all groups except control were immunized by multi-site injection (back, abdomen, subcutis, hind foot) of 5 mG bovine TG (bTg) in Freund’s adjuvant once every 2 weeks for a total of 6 weeks, during which drinking water containing Lugol’s iodine (5%) was supplied. Daily oral administration of YH, selenious and saline were conducted from the seventh week to the 12th week.

### Histological Examination

Hematoxylin and eosin (H and E) staining was carried out to examine the injury of thyroid gland. Thyroid glands were fixed in 4% paraformaldehyde and embedded in wax, followed by the preparation of 4 µM-thick sections. Then thyroid tissue slices were stained with hematoxylin and eosin.

### Biochemical Analysis

Serum TgAb, TPOAb, TSH, T3, and T4 were assayed using the ELISA kits (IMMCO Diagnostics, NY, United States), according to the manufacturer’s protocol. All samples were analyzed in duplicate.

### Immunofluorescence Staining

After deparaffinization, thyroid sections were performed with antigen retrieval in 0.01 M citrate buffer solution. Sections were then incubated with a primary antibody against NLRP3 at 4°C overnight, followed by incubation with secondary antibody. A fluorescence microscope (Eclipse TE2000-E, Nikon, Tokyo, Japan) was used to obtain images of the tissues.

### Western Blot

Equal amounts of total proteins were separated on 10% SDS-PAGE and transferred onto PVDF membranes. Protein-transferred membranes were blocked with 3% BSA and incubated with respective primary antibodies overnight at 4 °C. The membranes were subsequently rinsed with TBST and incubated with horseradish peroxidase (HRP)-conjugated secondary antibodies for 90 min at room temperature. The density of bands was visualized and determined by chemiluminescence.

### Real-Time Polymerase Chain Reaction

Total RNA was extracted using TRIzol reagent (Invitrogen, Paisley, United Kingdom) according to the manufacturer’s instructions. The purified mRNA was reverse transcribed into cDNA, and real-time PCR was performed using SYBR Premix Ex Taq (TaKaRa, Dalian, China). Data were standardized to the endogenous expression of GAPDH. The sequences of the primers are listed in Table 2.

**TABLE 2:** Sequences of the Real-time PCR primers.

Gene	Forward	Reverse
FoxP3	AGC​CTG​CCT​CAG​ACA​AGA​AC	GAA​GAA​GAG​GAG​GTG​TGG​GC
TGF-β	CAC​TCC​CGT​GGC​TTC​TAG​TG	GGA​CTG​GCG​AGC​CTT​AGT​TT
IL-10	TGC​CCC​AGG​CAG​AGA​ACC​AT	TCT​TCA​CCT​GCT​CCA​CTG​CC
RORγt	GTG​CAA​TGT​GGC​CTA​CTC​CT	GCA​GAC​TGT​CCC​TCT​GCT​TC
IL-17A	TGA​AGG​CAG​CGG​TAC​TCA​TC	GGG​TGA​AGT​GGA​ACG​GTT​GA
IL-21	CCG​TGG​CCC​ATA​AAT​CAA​G	GGA​GCT​GTT​AGA​AGT​TCA​GG
IL-22	CCA​TAC​ATC​GTC​AAC​CGC​AC	GAC​TCC​TCG​GAA​CAG​TTC​CT
IL-12A	CAG​TCT​CTG​GAC​CTG​CCA​AG	GTG​CTG​GTT​TTG​TCC​CGT​GT
Ebi3	TAC​CCT​GTG​GCT​GTG​GAC​TG	GTG​GAA​AAC​AGG​TGC​ACG​TG
Wnt-1	GCA​AGC​AGC​GAC​GAC​TGA​TCC	TCT​CGG​CAG​CCT​CGG​TTG​AC
β-catenin	GTT​GCT​CCA​CTC​CAG​GAA​TGA​AGG	GCA​CCA​ATG​TCC​AGT​CCG​AGA​TC
GAPDH	TGA​TGG​GTG​TGA​ACC​ACG​AG	AGT​GAT​GGC​ATG​GAC​TGT​GG

### Flow Cytometry

The flow cytometry analysis was performed as previously described with minor modifications (Ma et al., 2016). Briefly, cells were incubated with CD4 and CD25, followed by fixation in fixation/permeabilization buffer. Then cells were stained with antibodies against FoxP3 and IL-17. All testing was performed on a BD FacsCalibur instrument and analyzed using the CellQuest software (BD Biosciences).

### Network Pharmacology Analysis of YH in AIT Treatment

To clarify the anti-thyroiditis ingredients of YH, the network pharmacology analysis was conducted. Firstly, we searched the CAS IDs and canonical SMILES strings of the major constituents including 2-Hexaprenyl-6-methoxy-1,4-benzoquinone, 4-Amino-1-piperidinecarboxylic acid, 5-Megastigmen-7-yne-3, 9-diol 3-glucoside, Corchoionoside B, Ilicifolinoside A, Moclobemide, Molludistin 2″-rhamnoside, Perilloside C, Sampangine, Solanolone, Sterol, 2-Dodecylbenzenesulfonic acid, 3,4′,5-Trihydroxy-3′,7-dimethoxyflavanone, 3-O-Caffeoyl-1-O-methylquinic acid, Carvyl propionate, Eremopetasidione, Glucoliquiritin apioside, Inulobiose, Isoliquiritin, Naringin 6″-rhamnoside and Neolicurosidein YH decoction on Pubchem database (https://pubchem.ncbi.nlm.nih.gov/) (Zhang et al., 2020). Afterward, the canonical SMILES strings were imported into the Swiss TargetPrediction tool (www.swisstargetprediction.ch/), a web server for potential drug target prediction by reversed pharmacophore matching query compound against an in-house pharmacophore model database, in order to predict the potential targets of each major compound (Huang et al., 2020). Furthermore, we applied R software version 4.0.2 (http://www.r-project.org) with several R packages, including clusterProfifiler (Yu et al., 2012) and org. Hs.eg.db (version 3.8.2; bioconductor.org/packages/org.Hs.eg.db), for pathway enrichment analysis by the use of the predicted targets. The compound-target-pathway network was then established with the software Cytoscape-v3.7.2 (Shannon et al., 2003) for understanding the complex relationships and revealing the molecular mechanisms (Wang L. et al., 2020).

### Statistical Analysis

All values are expressed as mean ± SEM. The statistical significance was determined by one-way ANOVA with post-hoc Bonferroni test, using GraphPad prism statistical software version 5.0. *p* < 0.05 was taken as statistically significant.

## Results

### Histopathology of Thyroid Inflammation

**FIGURE 2:**
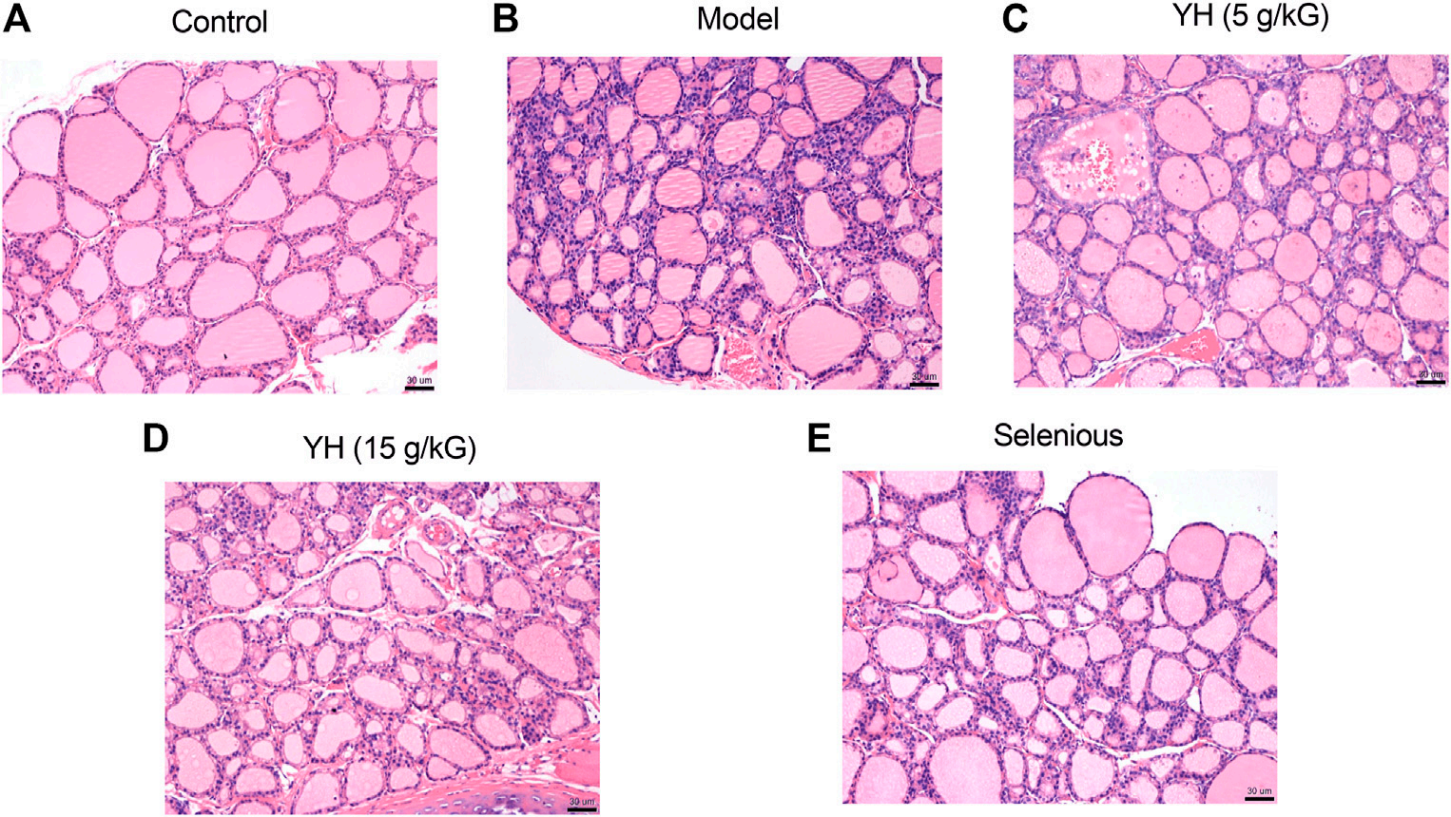
Hematoxylin and eosin staining of thyroid tissue from rats **(A)** control group **(B)** model group **(C)** YH 5 g crude drug/kG group **(D)** YH 15 g crude drug/kG group **(E)** selenious group. Magnification times, ×20.

As demonstrated in Figure 2, rats in the control group had rounded or ovoid thyroid follicles, and there was no evidence of lymphoid infiltration in the thyroid tissue. In contrast to control group, clear damage and atrophy of the thyroid follicles, the presence of epithelial hyperplasia and clear evidence of interstitial lymphoid infiltration were observed in EAT rats. Nonetheless, YH administration (5 g crude drug/kG; 15 g crude drug/kG) noticeably attenuated EAT-elicited thyroid gland injury, epithelial hyperplasia and infiltration of inflammatory cells. These findings suggested that YH could decrease thyroid lesions and lymphoid infiltration of the thyroid.

### YH Improved Serum TgAb, TPOAb, TSH, T3, and T4

**FIGURE 3:**
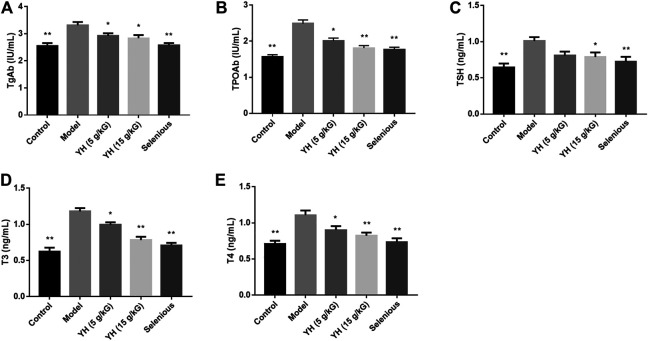
Serum levels of TgAb, TPOAb, and thyroid function-related parameters among groups **(A)** TgAb **(B)** TPOAb **(C)** TSH **(D)** T3 **(E)** T4. All values are expressed as means ± SEM. *p < 0.05, **p < 0.01 vs Model.

Concentrations of serum TgAb, TPOAb, TSH, T3, and T4 are shown in Figure 3. EAT rats presented an increased expression of these cytokines in the serum compared with those of normal controls. However, compared with the model group, YH administration (5 g crude drug/kG; 15 g crude drug/kG) partially or completely reversed the increase of TgAb, TPOAb, TSH, T3, and T4 in EAT rats.

### YH Increased the mRNA Levels of IL-35 Subsets (IL-12A and EBI3) in PBMCs

**FIGURE 4:**
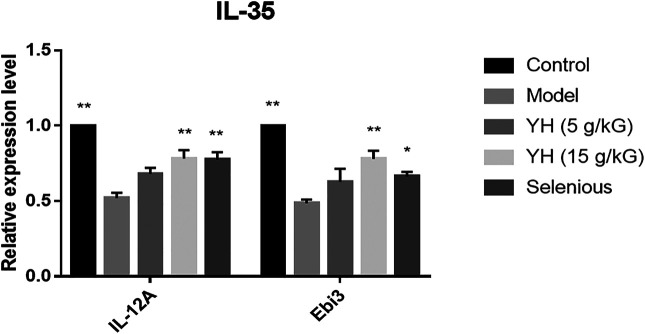
The mRNA levels of the IL-35 subunit (IL-12A, EBI3) in peripheral blood mononuclear cell (PBMCs). All values are expressed as mean ± SEM. *p < 0.05, **p < 0.01 vs Model.

The serum IL-35 levels and the mRNA levels of IL-35 subsets were measured because IL-35 upregulation plays a critical role in autoimmune diseases prevention. As illustrated in Figure 4, the mRNA levels of IL-35 subsets including IL-12A and EBI3 in PBMCs were also evidently lower than those in the non-immunized group. With YH intervention (15 g crude drug/kG), both the serum IL-35 level and the expression of IL-12A and EBI3 in PBMCs were greatly increased when compared to the EAT group.

### YH Rescued Th17/Treg Imbalance in PBMCs

**FIGURE 5:**
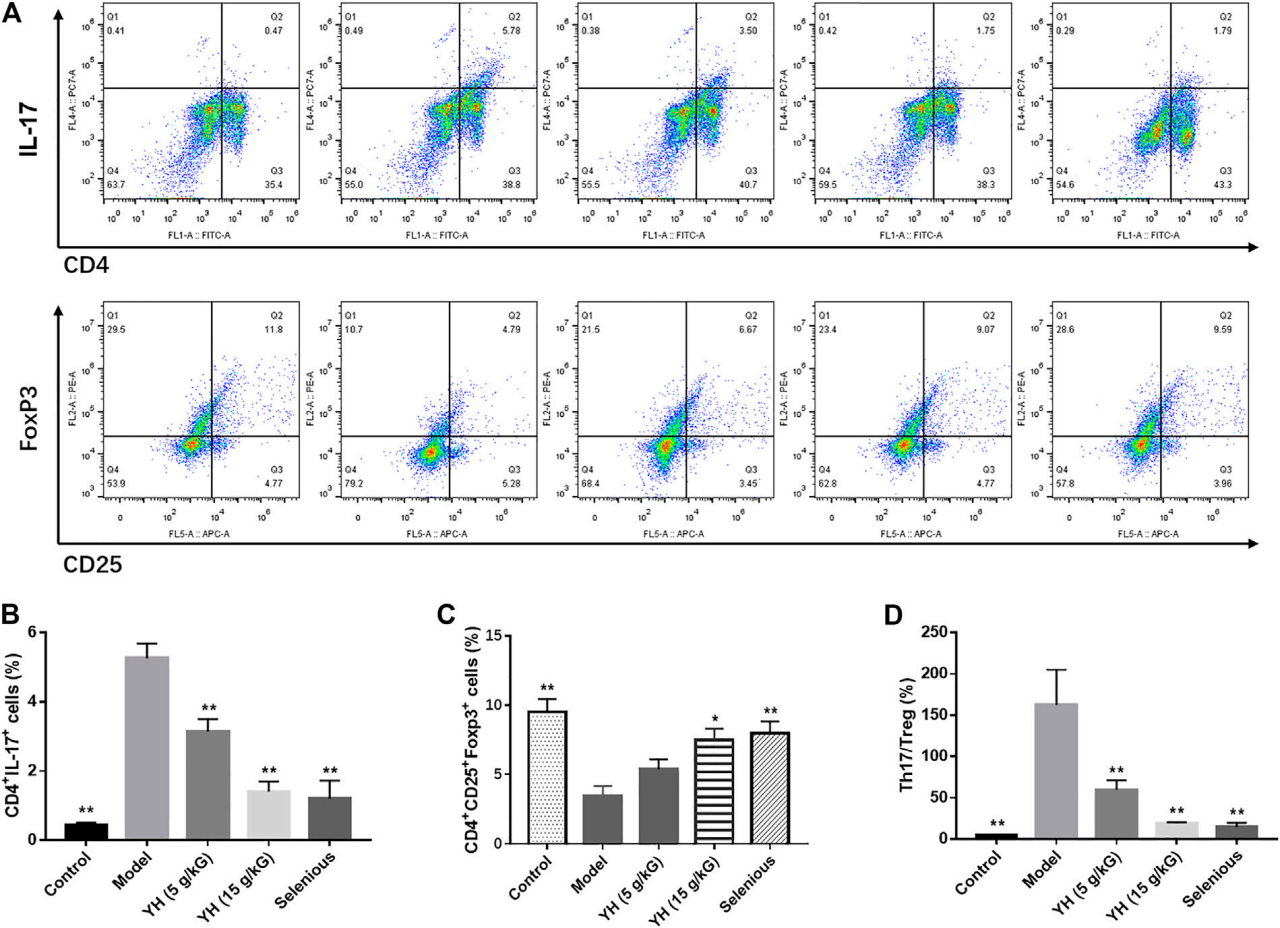
Changes of Th17 and Treg cell percentages in peripheral blood mononuclear cell (PBMCs) **(A)** Representative graphics from flow cytometry for Th17 and Treg measurements. Peripheral blood mononuclear cells were incubated with different antibodies and subjected to flow cytometry analysis. Th17 (CD4^+^IL-17^+^) flow cytometry analysis graphics. Treg (CD4^+^ CD25 ^+^ Foxp3+) flow cytometry analysis graphics. Th17 **(B)** and Treg **(C)** percentage in PMBCs measured by flow cytometry **(D)** Th17/Treg ratio. All values are expressed as mean ± SEM. *p < 0.05, **p < 0.01 vs Model.

As IL-35 is mainly synthesized by Tregs, flow cytometry and RT-PCR were carried out to investigate the role of Treg and Th17 cells in the therapeutic effects of YH on EAT model. According to flow cytometric analysis, the EAT challenge induced a significant increase in the proportion of Th17 cells (CD4^+^IL-17A^+^), and a substantial reduction in the proportion of Treg cells (CD4^+^CD25^+^Foxp3^+^) to CD4^+^ T cells (Figure 5). Moreover, the Th17/Treg ratio was dramatically promoted in model group compared with their control counterparts. Nonetheless, YH intervention increased the percentage of Treg cells (15 g crude drug/kG), and decreased the proportion of Th17 cells (5 g crude drug/kG; 15 g crude drug/kG) as well as Th17/Treg ratio (5 g crude drug/kG; 15 g crude drug/kG). Collectively, above findings suggested that YH could prevent against Th17/Treg imbalance induced by EAT modeling.

**FIGURE 6:**
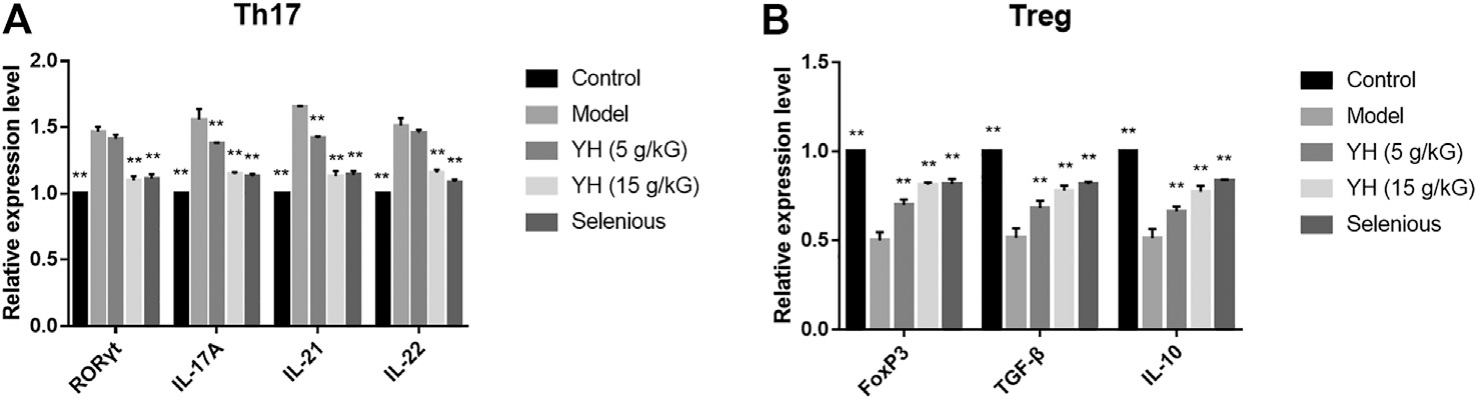
Th17/Treg-related mRNA expression in peripheral blood mononuclear cell (PBMCs) **(A)** mRNA levels of RORγt, IL-17A, IL-21, IL-22 **(B)** mRNA levels of FoxP3, TGF-β, and IL-10. All values are expressed as mean ± SEM. **p < 0.01 vs Model.

The RT-PCR analysis revealed that the RORγt, IL-17A, IL-21 and IL-22 mRNA levels were significantly upregulated in PBMCs relative to the control group. Meanwhile, the expressions of FoxP3, TGF-β, and IL-10 mRNA were significantly decreased (Figure 6). After the treatment with YH, we observed decreased mRNA levels of RORγt, IL-17A, IL-21, and IL-22 and enhanced mRNA expressions of FoxP3, TGF-β, and IL-10 in EAT rats.

### YH Repaired Wnt/β-Catenin Pathway in PBMCs

**FIGURE 7:**
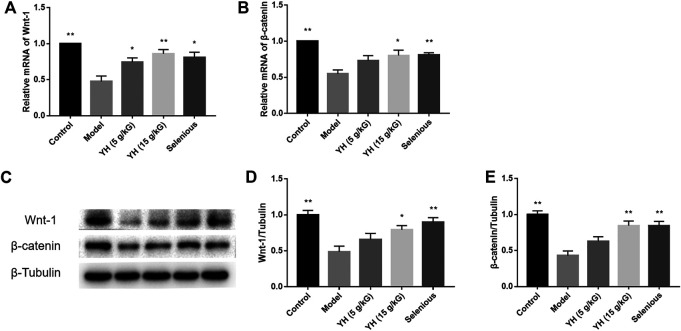
RT-PCR and Western blot analysis for the expression of molecules in the Wnt/β-catenin signaling pathway in peripheral blood mononuclear cell (PBMCs) **(A)** mRNA expression of Wnt-1 and β-catenin **(B)** Western blotting images of Wnt-1and β-catenin: Lane 1, control group; lane 2, model group; lane 3, YH 5 g crude drug/kG group; lane 4, YH 15 g crude drug/kG group; lane 5, selenious group **(C)** Bar graphs indicate the relative ratio of Wnt-1and β-catenin over tubulin. All values are expressed as mean ± SEM. *p < 0.05, **p < 0.01 vs Model.

Since inhibition of β-catenin was documented to disturb Th17/Treg homeostasis in chronic obstructive pulmonary disease (COPD) (Zhou et al., 2019), changes of Wnt/β-catenin signaling in the PBMCs were detected using RT-PCR and western blot in this study. As illustrated in Figure 7, the mRNA levels of Wnt-1 and β-catenin in EAT rats were evidently lower than those in the non-immunized group. With YH intervention, the expressions of Wnt-1 (5 g crude drug/kG; 15 g crude drug/kG) and β-catenin (15 g crude drug/kG) were greatly increased when compared to the model group. The western blot analysis showed that the protein expression of Wnt-1 and β-catenin were significantly inhibited in the thyroid of EAT rats, whereas YH administration (15 g crude drug/kG) evidently upregulated the levels of Wnt-1 and β-catenin compared with model group. These findings indicated that YH treatment could alleviate EAT-induced inflammasome activation in Wnt/β-catenin pathway.

### YH Inhibited NLRP3 Inflammasome in Thyroid

**FIGURE 8:**
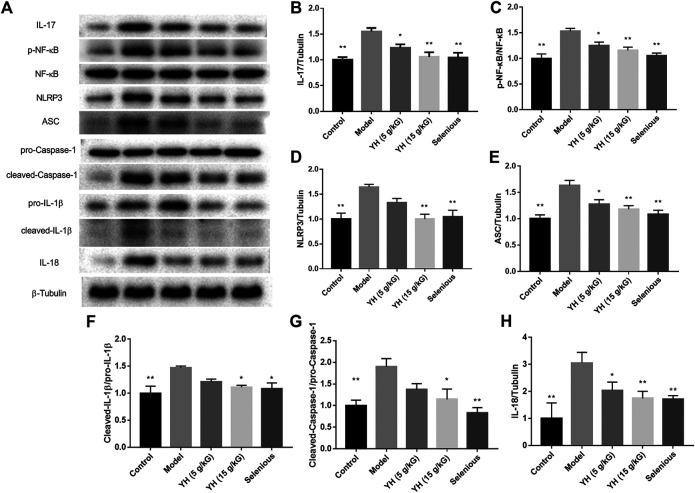
Western blot analysis of NLRP3 inflammasome cascade in thyroid **(A)** Western blotting images of IL-17, p-NF-κB, NF-κB, NLRP3, ASC, cleaved-Caspase1, pro-Caspase 1, pro-IL-1β, cleaved-IL-1β, and IL-18: Lane 1, control group; lane 2, model group; lane 3, YH 5 g crude drug/kG group; lane 4, YH 15 g crude drug/kG group; lane 5, selenious group. Bar graphs indicate the relative ratio of IL-17 over tubulin **(B)**, p-NF-κB over NF-κB **(C)**, NLRP3 over tubulin **(D)**, ASC over tubulin **(E)**, cleaved-Caspase-1 over pro-Caspase-1 **(F)**, cleaved-IL-1β over pro-IL-1β **(G)**, and IL-18 over tubulin **(H)** in thyroid. All values are expressed as mean ± SEM. *p < 0.05, **p < 0.01 vs Model.

**FIGURE 9:**
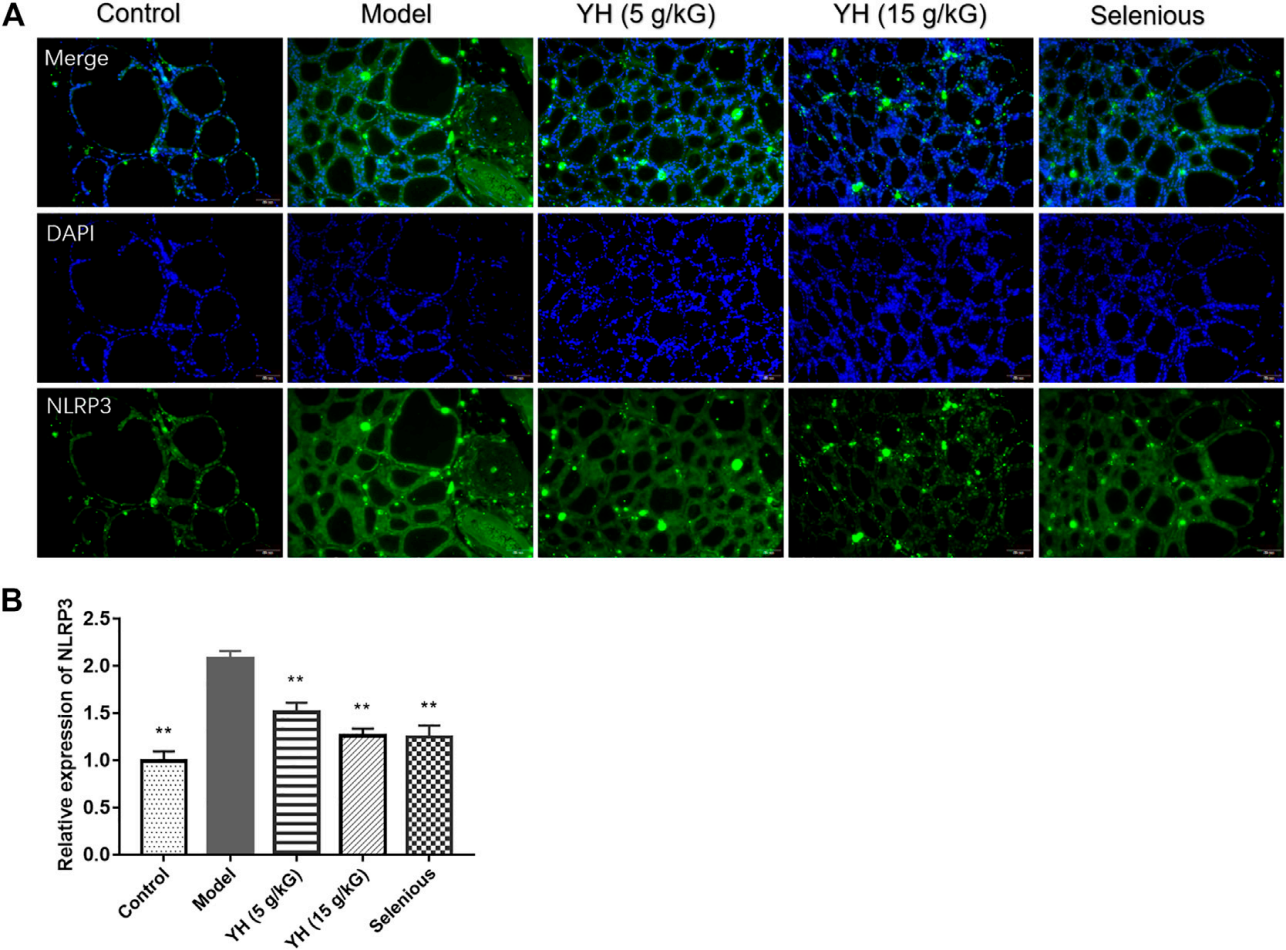
Immunofluorescence staining for NLRP3 in thyroid **(A)** Representative image of immunofluorescence staining of NLRP3 **(B)** Quantification graph of NLRP3 immunofluorescence. All values are expressed as mean ± SEM. **p < 0.01 vs Model.

It has been reported that Treg differentiation was negatively regulated by NLRP3 (Park et al., 2019). We then tested the effect of YH on inflammasome activation applying western blot and immunofluorescence staining (Figures 8, 9). The results showed that rats underwent EAT challenge exhibited increased immunostaining signal for NLRP3 and elevated protein levels of IL-17, p-NF-κB, NLRP3, ASC, cleaved-Caspase1, cleaved-IL-1β and IL-18 in the thyroid when compared to non-immunized rats, suggesting the activation of NLRP3 inflammasome in EAT model. Interestingly, 5 g crude drug/kG of YH profoundly inhibited the aforementioned alternations except for NLRP3 protein expression, while 15 g crude drug/kG of YH suppressed these changes completely, indicating the effect of YH on deactivating the NLRP3 inflammasome in the thyroid.

### Network Pharmacology-Based Strategy for Predicting Active Ingredients of YH in Treating AIT

**FIGURE 10:**
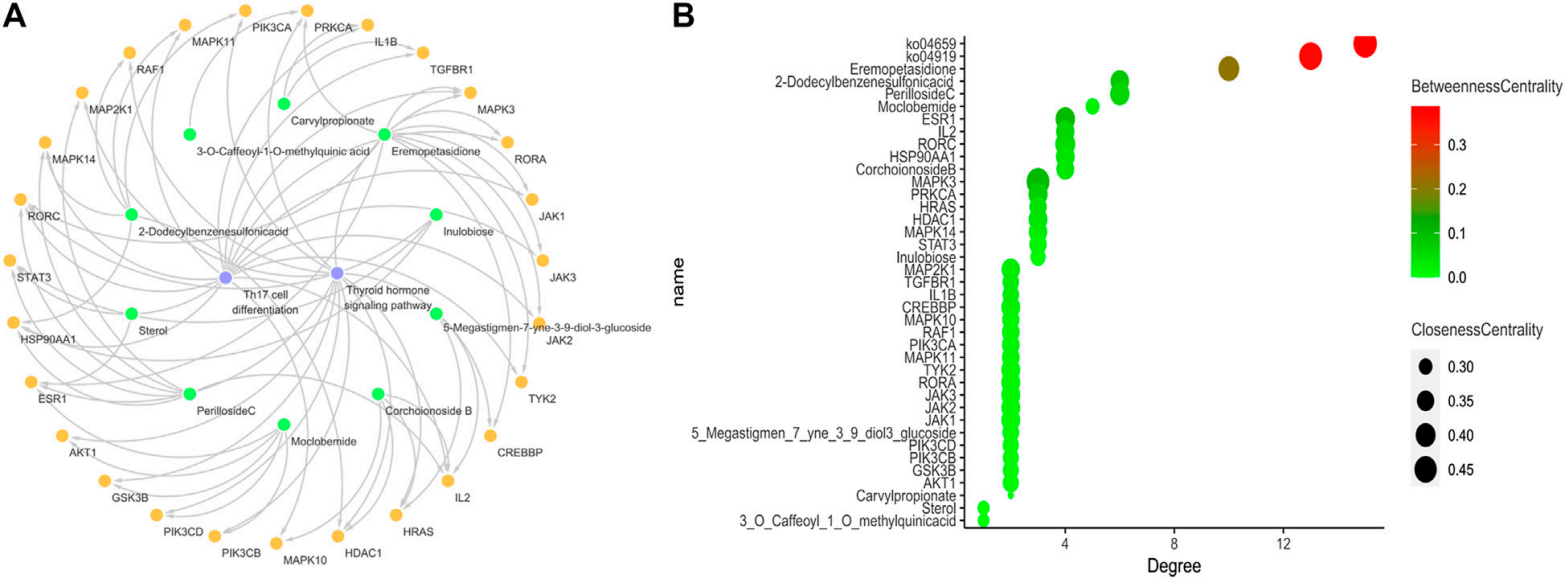
**(A)** Compound-target-pathway network. Nodes in different colors represent different groups: green for compound, yellow for targets, purple for pathways **(B)** The centrality of nodes was evaluated according to the degree centrality, betweenness centrality, and closeness centrality. KEGG ID: ko04919, thyroid hormone signaling pathway; KEGG ID: ko04659, Th17 cell differentiation signal pathway.

The identified ingredients were found to interact with 433 independent predicted targets by searching Swiss Target Prediction tool (Supplementary File). After KEGG pathway enrichment analysis, there were 136 significant pathways that met the screening principal Q value <0.05, including Th17 cell differentiation signal pathway (KEGG ID: ko04659) and thyroid hormone signaling pathway (KEGG ID: ko04919), as listed in Supplementary File. As demonstrated in Figure 10A, for revealing the molecular mechanisms, we constructed the compound-target-pathway network, which included 39 nodes and 68 edges. Furthermore, after analyzing the network (as shown in Supplementary File), we visualized the three important network parameters of the nodes, containing degree, closeness centrality and betweenness centrality. The details are shown in Figure 10B. These results suggested that Eremopetasidione, 2-Dodecylbenzenesulfonic acid, Perilloside C, and Moclobemide could be the key components for the anti-thyroiditis activity of YH.

## Discussion

Experimental autoimmune thyroiditis has been used to simulate human autoimmune thyroid disease for decades. It can be elicited by treatment with pTG and adjuvant, along with excessive iodine intake. Infiltration of lymphocytes into the thyroid, high levels of autoantibodies to thyroglobulin (Tg) and thyroperoxidase (TPO), and elevated thyroid hormones were observed on EAT model (Li et al., 2018). In our study, rats underwent EAT challenge displayed similar thyroid lesions and elevated serum TgAb, TPOAb, TSH, T3, and T4, indicating the development of EAT in rats. In addition, YH treatment mitigated the thyroid lesions and decreased serum TgAb, TPOAb, TSH, T3, and T4 levels, suggesting that YH could effectively ameliorate autoimmune thyroiditis in rats.

It was proposed that the IL-35 dysfunction was closely associated with AIT. A study found decreased IL-35 in the serum of AIT patients that was negatively associated with TPOAb and TSH levels (Yilmaz et al., 2016). IL-35 is a heterodimeric protein composed of two shared subunits, EBI3 (Epstein-Barr-Virus-induced gene 3) and IL-12a (p35).

Herein, we demonstrated that EAT challenge decreased the mRNA expressions of IL-35 submits (IL-12A and Ebi3) in PBMCs. YH treatment could reverse the decline of IL-12A and Ebi3 mRNA expression in PBMCs. IL-35 is a novel cytokine with therapeutic properties in diseases whose pathogenesis is associated with the Th17/Treg imbalance (Cafferata et al., 2020). Evidences showed that IL-35 attenuates the Th17/Treg imbalance through Wnt/β-catenin pathway (Yang et al., 2018; Li Y. et al., 2019). In this study, we found that EAT challenge decreased the mRNA and protein expression of Wnt-1 and β-catenin in the thyroid, confirming the disruption of Wnt/β-catenin axis in rats with AIT. YH treatment reversed this decrease, supporting the involvement of Wnt/β-catenin in YH-mediated protective effects in EAT rats.

Th17 and Treg are new lineages of CD4^+^ T helper cells that are responsible for immune response. While Th17 cells induce the secretion of pro-inflammatory cytokines, Treg cells restrict inflammatory responses and confer immune-tolerance. Recent studies have indicated that the imbalance of Th17/Treg cells is a crucial pathogeny of AIT. Liu and others investigated the alteration of Th17 and Treg cells in AIT patients and found an evident enhancement of Th17 cells in the peripheral blood from AIT patients accompanied by a reduction of Treg cells. Meanwhile, the Th17/Treg ratio in the PBMCs of AIT patients positively correlated with the TgAb levels (Liu et al., 2014). Selenium supplementation has been proven to alleviate the apoptosis of the follicular cells and decrease the levels of TPOAb and TSH in AT via inducing the differentiation of CD4+T cells into Treg cells (Duntas, 2015). In our study, EAT rats showed lower percentage of Treg cells and higher percentage of Th17 cells as well as increased Th17/Treg ratio in the PBMCs. Meanwhile, EAT rats exhibited enhanced RORγt, IL-17A, IL-21, and IL-22 mRNA expression and decreased FoxP3, TGF-β, and IL-10 mRNA levels in the PBMCs when compared with control group. These results indicated that there was a disruption of Th17/Treg balance in EAT rats. YH administration significantly altered these changes, demonstrating the restoration of Th17/Treg balance is involved in YH-induced protective effects in EAT.

Inflammation is a comprehensive array of physiological response to tissue damage, which is resulted from physical injury, infection, exposure to toxins, or other types of trauma. Mounting evidence indicated that inflammation is a major factor for the progression of a variety of diseases including autoimmune thyroiditis (Arulselvan et al., 2016). A study explored the link between inflammasomes and autoimmune thyroiditis, and revealed the activation of NLRP1, NLRC4, and AIM2 inflammasomes in the thyroid tissues from patients with AIT. Furthermore, the thyroid mRNA level of NLRP1 was related to the serum TPOAb and TgAb levels in the AIT group (Guo et al., 2018). Cytotoxic T-lymphocyte associated protein 4 (CTLA-4) inhibition worsened autoimmune thyroiditis in mice via the production of interleukin (IL)-2, interferon gamma, IL-10, and IL-13 cytokines (Sharma et al., 2016). NLRP3 inflammasome plays a crucial role in autoimmune thyroiditis. Promoted posttranslational modifications of Caspase-1, IL-1β and IL-18 were observed in the thyroid tissues from AIT patients; the thyroid ASC mRNA expression was correlated with the serum TPOAb and TgAb in AIT subjects (Guo et al., 2018). Th17 and Treg cells play important roles in regulating NLRP3 inflammasome. The imbalance of Th17/Treg has been reported to trigger NLRP3 inflammasome activation through the IL-17/NF-κB pathway (Wei et al., 2018; Yang et al., 2018). Therefore, we focused on the role of the NLRP3 inflammasome in the present study and found that EAT rats displayed increased NLRP3 immunostaining signal and protein expressions of NLRP3, ASC, cleaved-Caspase1, cleaved-IL-1β, and IL-18 in the thyroid, suggesting the activation of NLRP3 inflammation following EAT challenge. YH successfully reversed the expression of these proteins, reflecting that the beneficial effect of YH in EAT is attributed to the inhibition of the NLRP3 inflammation in the thyroid.

**FIGURE 11:**
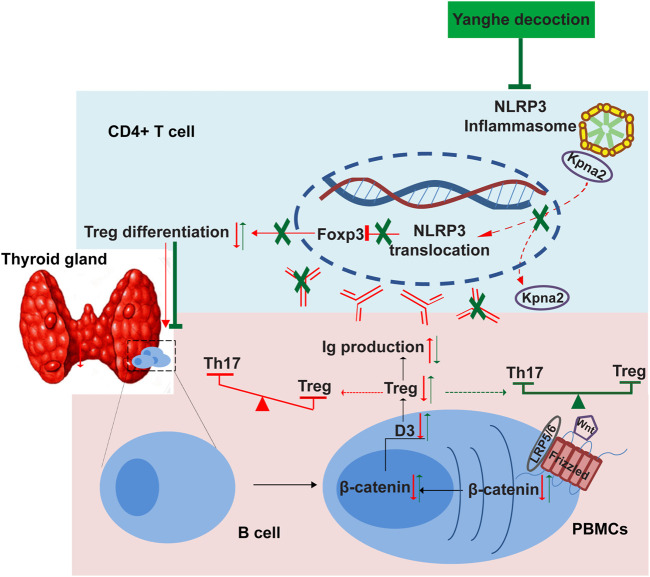
Schematic illustration of the Yanghe decoction negatively regulates NLRP3 inflammasome activation by modulated the Th17/Treg balance.

It has been reported that NLRP3 knockdown by using siRNA in CD4 T cells isolated from rheumatoid arthritis (RA) patients suppressed Th17 differentiation (Zhao et al., 2018). Park et al. demonstrated that NLRP3-deficient mice could elevate Treg generation in various organs in the *de novo* pathway (Park et al., 2019). These data suggested that NLRP3 inflammasome might participate in the regulation of Treg/Th17 cell balance in autoimmune diseases. In a recent study, Jin et al. confirmed that protect in DX (PDX) could restore Treg/Th17 cell balance in RA by inhibiting NLRP3 inflammasome via miR-20a (Jin et al., 2021). Considering that the NLRP3 inflammasome plays an important role in the Treg/Th17 cell balance, which in turn regulates autoimmunity, we speculated that YH treatment alleviated autoimmune thyroiditis in rats, which could be due to the Th17/Treg rebalancing regulated by Wnt/β-catenin pathway through NLRP3 inflammasome deactivation (Figure 11). To further verify this presumption, Nlrp3^−/−^mice should be used to construct an AIT model and intervene with YH decoction.

Recently, network pharmacology approach, a systematic analytical technology based on the development of bioinformatics, network biology and pharmacology analysis, has been broadly applied in TCM and emerged as an indispensable method for the development of TCM (Hopkins, 2008; Jiang et al., 2020; Wang Y. et al., 2020). Considering the commendable ability of network pharmacology approach in exploring the multi-components, multi-targets, and multi-pathways of TCM (Zhou et al., 2020), in the present study, we conduct the network pharmacology analysis to clarify the anti-thyroiditis ingredients of YH. The thyroid hormone signaling pathway and the Th17 cell differentiation signal pathway have been implicated in the pathogenesis of AIT since it is closely related to immune system. In the current study, we have found that YH could act on both signaling pathways using experimental evaluations and network pharmacology. As demonstrated in Figure 10B, Eremopetasidione, 2-Dodecylbenzenesulfonic acid, Perilloside C, and Moclobemide were considered to be the key constituents in the anti-thyroiditis activity of YH. Eremopetasidione, a nor-sesquiterpenoid was firstly found from the rhizomes of Petasites japonicus. Studies have shown that sesquiterpenoid and its analogues have immunosuppressive activities (Bartikova et al., 2014). Artemisinin, also a sesquiterpenoid compound, has good immunomodulatory effects and exerts the therapeutic effects on immune-related disorders via regulating the differentiation of CD4^+^ T cell subsets (Bai et al., 2019). 2-dodecylbenzenesulfonic acid, an AKT inhibitor targets AKT-PH domain, inhibits downstream signaling, and alleviates inflammation (Ahad et al., 2011). A study found that the Akt inhibitor triciribine decreased the severity score of thyroiditis significantly in the EAT model (Li et al., 2018). Perilloside C was found to be an inhibitor of aldose reductase (Fujita et al., 1995). Interestingly, aldose reductase is considered to be linked to thyroiditis in dog thyroid (Schaffhauser et al., 1996). Moclobemide is known as a reversible inhibitor of monoamine oxidase A. Some studies support that monoamine oxidase inhibitors can suppress TSH levels in a fairly high percentage of depression patients (Cabanillas et al., 1994; Kadono et al., 1995).

Given that the research results may be used as a guide to treat diseases, it is critical to discuss adequately for the risk of excessive high doses used in the field of ethnopharmacology (Heinrich et al., 2020). YH decoction has been clinically used as a classical TCM formula in China for thousands of years. 55 g crude drug/70 kG/day is usually used for patients. The rat dose in this study was converted from the human dose based on the body surface area, as was reported (Nair and Jacob, 2016). Therefore, 5 g crude drug/kG is close to the human therapeutic dose and 15 g crude drug/kG is the 3-fold of the human therapeutic dose. In this article higher doses of the crude extract were needed to achieve the effect, consistent with previous research demonstrating a dose of 21.6 g/kg Linggan Wuwei Jiangxin decoction was used in a rat study on cold asthma treatment (Ran et al., 2019). Decoction is the most common form with an average of about 12 ingredients in Chinese medicine. Crude herbs in the amount of 120 g in a decoction is usual. Such large doses of herbs are needed maybe because the majority of the weight of material, (e.g. plant fiber) that is being cooked up is not consumed. In the consumed materials, only a relatively small proportion of the substances might be active ingredients. While the majority such as sugars, starches and other ordinary ingredients are not expected to have a significant biological activity. As described above, in most previous studies the dosage of the decoction reached g crude drug/kG/day in rodents (Li S. et al., 2019; Luo et al., 2019). Future study deserves to repeat the experiment at lower doses levels for evaluating YH decoction’s anti-AIT potential.

Collectively, the present study indicated that YH treatment significantly protected against autoimmune thyroiditis in EAT rats, which was due to improve NLRP3 inflammasome and immune dysregulation. Based on a systematic network pharmacology approach and results of this work, we predicted that Eremopetasidione, 2-Dodecylbenzenesulfonic acid, Perilloside C, and Moclobemide are the main active anti-thyroiditis ingredients of YH. Our findings provide preclinical evidence of the therapeutic benefit of YH decoction in autoimmune thyroiditis and reveals the related immune/inflammatory mechanism.

## Data Availability

The original contributions presented in the study are included in the article/Supplementary Material, further inquiries can be directed to the corresponding authors.

## References

[B1] Ahad A. M. Zuohe S. Du-Cuny L. Moses S. A. Zhou L. L. Zhang S. (2011). Development of Sulfonamide AKT PH Domain Inhibitors. Bioorg. Med. Chem. 19 (6), 2046–2054. 10.1016/j.bmc.2011.01.049 21353784 PMC3088502

[B2] Arulselvan P. Fard M. T. Tan W. S. Gothai S. Fakurazi S. Norhaizan M. E. (2016). Role of Antioxidants and Natural Products in Inflammation. Oxid Med Cell Longev 2016, 5276130. 10.1155/2016/5276130 27803762 PMC5075620

[B3] Bai L. Li H. Li J. Song J. Zhou Y. Liu B. (2019). Immunosuppressive Effect of Artemisinin and Hydroxychloroquine Combination Therapy on IgA Nephropathy via Regulating the Differentiation of CD4+ T Cell Subsets in Rats. Int. Immunopharmacology 70, 313–323. 10.1016/j.intimp.2019.02.056 30852287

[B4] Bartikova H. Hanusova V. Skalova L. Ambroz M. Bousova I. (2014). Antioxidant, Pro-oxidant and Other Biological Activities of Sesquiterpenes. Ctmc 14 (22), 2478–2494. 10.2174/1568026614666141203120833 25478887

[B5] Cabanillas A. M. Masini-Repiso A. M. Costamagna M. E. Pellizas C. Coleoni A. H. (1994). Thyroid Iodide Transport Is Reduced by Administration of Monoamine Oxidase A Inhibitors to Rats. J. Endocrinol. 143 (2), 303–308. 10.1677/joe.0.1430303 7829993

[B6] Cafferata E. A. Terraza‐Aguirre C. Barrera R. Faúndez N. González N. Rojas C. (2020). Interleukin‐35 Inhibits Alveolar Bone Resorption by Modulating the Th17/Treg Imbalance during Periodontitis. J. Clin. Periodontol. 47 (6), 676–688. 10.1111/jcpe.13282 32160331

[B8] Cui S. L. Yu J. Shoujun L. (2014). Iodine Intake Increases IP-10 Expression in the Serum and Thyroids of Rats with Experimental Autoimmune Thyroiditis. Int. J. Endocrinol. 2014, 1–7. 10.1155/2014/581069 PMC3953660 24707288

[B9] Duntas L. (2015). The Role of Iodine and Selenium in Autoimmune Thyroiditis. Horm. Metab. Res. 47 (10), 721–726. 10.1055/s-0035-1559631 26361258

[B10] Fujita T. Ohira K. Miyatake K. Nakano Y. Nakayama M. (1995). Inhibitory Effect of Perillosides A and C, and Related Monoterpene Glucosides on Aldose Reductase and Their Structure-Activity Relationships. Chem. Pharm. Bull. 43 (6), 920–926. 10.1248/cpb.43.920 7641310

[B11] Guo Q. Wu Y. Hou Y. Liu Y. Liu T. Zhang H. (2018). Cytokine Secretion and Pyroptosis of Thyroid Follicular Cells Mediated by Enhanced NLRP3, NLRP1, NLRC4, and AIM2 Inflammasomes Are Associated with Autoimmune Thyroiditis. Front. Immunol. 9, 1197. 10.3389/fimmu.2018.01197 29915579 PMC5994487

[B12] Heinrich M. Appendino G. Efferth T. Fürst R. Izzo A. A. Kayser O. (2020). Best Practice in Research - Overcoming Common Challenges in Phytopharmacological Research. J. Ethnopharmacology 246, 112230. 10.1016/j.jep.2019.112230 31526860

[B13] Hopkins A. L. (2008). Network Pharmacology: the Next Paradigm in Drug Discovery. Nat. Chem. Biol. 4 (11), 682–690. 10.1038/nchembio.118 18936753

[B14] Hou Y. Guo X. Zhang C. Wang T. Guo X. Sun W. (2018). Protective Effects of Jiayan Kangtai Granules on Autoimmune Thyroiditis in a Rat Model by Modulating Th17/Treg Cell Balance. J. Tradit Chin. Med. 38 (3), 380–390. 10.1016/s0254-6272(18)30628-9 32185970

[B15] Huang S. J. Mu F. Li F. Wang W. J. Zhang W. Lei L. (2020). Systematic Elucidation of the Potential Mechanism of Erzhi Pill against Drug-Induced Liver Injury via Network Pharmacology Approach. Evid. Based Complement Alternat. Med. 2020, 1–15. 10.1155/2020/6219432 PMC6970004 31998398

[B16] Jiang Y. Zhong M. Long F. Yang R. (2020). Deciphering the Active Ingredients and Molecular Mechanisms of Tripterygium Hypoglaucum (Levl.) Hutch against Rheumatoid Arthritis Based on Network Pharmacology. Evid. Based Complement Alternat. Med. 2020, 1–9. 10.1155/2020/2361865 PMC6982362 32015751

[B17] Jin S. Sun S. Ling H. Ma J. Zhang X. Xie Z. (2021). Protectin DX Restores Treg/Th17 Cell Balance in Rheumatoid Arthritis by Inhibiting NLRP3 Inflammasome via miR-20a. Cell Death Dis 12 (3), 280. 10.1038/s41419-021-03562-6 33723242 PMC7961047

[B18] Kadono Y. Kaneda H. Maeda K. (1995). Effects of Antidepressants on Thyroid Stimulating Hormone Release in Rats under Ether Stress. Psychiatry Clin. Neurosci. 49 (4), 231–236. 10.1111/j.1440-1819.1995.tb01890.x 9179943

[B19] Li C. Yuan J. Zhu Y. F. Yang X. J. Wang Q. Xu J. (2016). Imbalance of Th17/Treg in Different Subtypes of Autoimmune Thyroid Diseases. Cell Physiol Biochem 40 (1-2), 245–252. 10.1159/000452541 27855396

[B20] Li H. Min J. Mao X. Wang X. Yang Y. Chen Y. (2018). Edaravone Ameliorates Experimental Autoimmune Thyroiditis in Rats through HO-1-dependent STAT3/PI3K/Akt Pathway. Am. J. Transl Res. 10 (7), 2037–2046. 30093941 PMC6079139

[B21] Li S. Qian Y. Xie R. Li Y. Jia Z. Zhang Z. (2019). Exploring the Protective Effect of ShengMai-Yin and Ganmaidazao Decoction Combination against Type 2 Diabetes Mellitus with Nonalcoholic Fatty Liver Disease by Network Pharmacology and Validation in KKAy Mice. J. Ethnopharmacology 242, 112029. 10.1016/j.jep.2019.112029 31216433

[B22] Li Y. Yuan L. Jiang S. Liu S. Xia L. Shen H. (2019). Interleukin-35 Stimulates Tumor Necrosis Factor-α Activated Osteoblasts Differentiation through Wnt/β-Catenin Signaling Pathway in Rheumatoid Arthritis. Int. Immunopharmacology 75, 105810. 10.1016/j.intimp.2019.105810 31404890

[B23] Lin F. Huang X. Xing F. Xu L. Zhang W. Chen Z. (2020). Semen Brassicae Reduces Thoracic Aortic Remodeling, Inflammation, and Oxidative Damage in Spontaneously Hypertensive Rats. Biomed. Pharmacother. 129, 110400. 10.1016/j.biopha.2020.110400 32570115

[B24] Liu J. Mao C. Dong L. Kang P. Ding C. Zheng T. (2019). Excessive Iodine Promotes Pyroptosis of Thyroid Follicular Epithelial Cells in Hashimoto's Thyroiditis through the ROS-NF-kappaB-NLRP3 Pathway. Front. Endocrinol. (Lausanne) 10, 778. 10.3389/fendo.2019.00778 31824415 PMC6880659

[B25] Liu Y. Tang X. Tian J. Zhu C. Peng H. Rui K. (2014). Th17/Treg Cells Imbalance and GITRL Profile in Patients with Hashimoto's Thyroiditis. Ijms 15 (12), 21674–21686. 10.3390/ijms151221674 25429429 PMC4284671

[B26] Luo S. Wen R. Wang Q. Zhao Z. Nong F. Fu Y. (2019). Rhubarb Peony Decoction Ameliorates Ulcerative Colitis in Mice by Regulating Gut Microbiota to Restoring Th17/Treg Balance. J. Ethnopharmacology 231, 39–49. 10.1016/j.jep.2018.08.033 30170079

[B27] Ma S. Chen X. Wang L. Wei Y. Ni Y. Chu Y. (2017). Repairing Effects of ICAM-1-Expressing Mesenchymal Stem Cells in Mice with Autoimmune Thyroiditis. Exp. Ther. Med. 13 (4), 1295–1302. 10.3892/etm.2017.4131 28413469 PMC5377266

[B28] Ma Y.-H. Zhang J. Chen X. Xie Y.-F. Pang Y.-H. Liu X.-J. (2016). Increased CD4+CD45RA-FoxP3lowcells Alter the Balance between Treg and Th17 Cells in Colitis Mice. Wjg 22 (42), 9356–9367. 10.3748/wjg.v22.i42.9356 27895423 PMC5107699

[B29] Mondal S. Pahan K. (2015). Cinnamon Ameliorates Experimental Allergic Encephalomyelitis in Mice via Regulatory T Cells: Implications for Multiple Sclerosis Therapy. PLoS One 10 (1), e0116566. 10.1371/journal.pone.0116566 25569428 PMC4287621

[B30] Nair A. Jacob S. (2016). A Simple Practice Guide for Dose Conversion between Animals and Human. J. Basic Clin. Pharma 7 (2), 27–31. 10.4103/0976-0105.177703 PMC4804402 27057123

[B31] Park S.-H. Ham S. Lee A. Möller A. Kim T. S. (2019). NLRP3 Negatively Regulates Treg Differentiation through Kpna2-Mediated Nuclear Translocation. J. Biol. Chem. 294 (47), 17951–17961. 10.1074/jbc.ra119.010545 31597697 PMC6879348

[B32] Ran S. Sun F. Song Y. Wang X. Hong Y. Han Y. (2019). The Study of Dried Ginger and Linggan Wuwei Jiangxin Decoction Treatment of Cold Asthma Rats Using GC-MS Based Metabolomics. Front. Pharmacol. 10, 284. 10.3389/fphar.2019.00284 31031619 PMC6470627

[B33] Schaffhauser M. A. Sato S. Kador P. F. (1996). NADPH-dependent Reductases in Dog Thyroid: Comparison of a Third Enzyme "glyceral Hyde Reductase" to Dog Thyroid Aldehyde Reductase. Int. J. Biochem. Cel Biol. 28 (3), 275–284. 10.1016/1357-2725(95)00147-6 8920636

[B34] Shannon P. Markiel A. Ozier O. Baliga N. S. Wang J. T. Ramage D. (2003). Cytoscape: a Software Environment for Integrated Models of Biomolecular Interaction Networks. Genome Res. 13 (11), 2498–2504. 10.1101/gr.1239303 14597658 PMC403769

[B35] Sharma R. Di Dalmazi G. Caturegli P. (2016). Exacerbation of Autoimmune Thyroiditis by CTLA-4 Blockade: A Role for IFNγ-Induced Indoleamine 2, 3-Dioxygenase. Thyroid 26 (8), 1117–1124. 10.1089/thy.2016.0092 27296629 PMC4976247

[B36] Wan X. Xu C. Yu C. Li Y. (2016). Role of NLRP3 Inflammasome in the Progression of NAFLD to NASH. Can. J. Gastroenterol. Hepatol. 2016, 6489012. 10.1155/2016/6489012 27446858 PMC4904645

[B37] Wang L. Tan N. Wang H. Hu J. Diwu W. Wang X. (2020). A Systematic Analysis of Natural α-glucosidase Inhibitors from Flavonoids of Radix Scutellariae Using Ultrafiltration UPLC-TripleTOF-MS/MS and Network Pharmacology. BMC Complement. Med. Ther. 20 (1), 72. 10.1186/s12906-020-2871-3 32143602 PMC7076893

[B38] Wang Y. Ru Y. Zhuo G. Sheng M. Wang S. Ma J. (2020). Investigation of the Potential Mechanism Governing the Effect of the Shen Zhu San on COVID-19 by Network Pharmacology. Evid. Based Complement Alternat. Med. 2020, 1–23. 10.1155/2020/8468303 PMC7669347 33224256

[B39] Wang Y. Xiao X. (2018). Clinical Efficacy of Modified Yanghe Decoction in Ankylosing Spondylitis: A Randomized Controlled Trial. Med. Sci. Monit. 24, 2912–2918. 10.12659/msm.909740 29735967 PMC5965017

[B40] Wei L. Xiong H. Li W. Li B. Cheng Y. (2018). Upregulation of IL ‐6 Expression in Human Salivary Gland Cell Line by IL ‐17 via Activation of P38 MAPK , ERK , PI 3K/Akt, and NF ‐κB Pathways. J. Oral Pathol. Med. 47 (9), 847–855. 10.1111/jop.12765 30007092

[B41] Wu D. H. Xu L. Xie G. Q. Fan Y. S. Zhou J. (2019). The Pungent and Hot Chinese Herbs Cause Heat Syndrome in Rats by Affecting the Regulatory T Cells. Evid. Based Complement Alternat. Med. 2019, 1–9. 10.1155/2019/9824906 PMC6652065 31360212

[B42] Yang Y. Zhang X. Xu M. Wu X. Zhao F. Zhao C. (2018). Quercetin Attenuates Collagen-Induced Arthritis by Restoration of Th17/Treg Balance and Activation of Heme Oxygenase 1-mediated Anti-inflammatory Effect. Int. Immunopharmacology 54, 153–162. 10.1016/j.intimp.2017.11.013 29149703

[B43] Yilmaz H. Cakmak M. Ceydilek B. Demir C. Aktas A. (2016). Role of Interlekin-35 as a Biomarker in Patients with Newly Diagnosed Hashimoto's Thyroiditis. Endocr. Regul. 50 (2), 55–61. 10.1515/enr-2016-0009 27560637

[B44] Yu G. Wang L.-G. Han Y. He Q.-Y. (2012). clusterProfiler: an R Package for Comparing Biological Themes Among Gene Clusters. OMICS: A J. Integr. Biol. 16 (5), 284–287. 10.1089/omi.2011.0118 PMC3339379 22455463

[B45] Zeng J. Chen Y. Ding R. Feng L. Fu Z. Yang S. (2017). Isoliquiritigenin Alleviates Early Brain Injury after Experimental Intracerebral Hemorrhage via Suppressing ROS- And/or NF-kappaB-Mediated NLRP3 Inflammasome Activation by Promoting Nrf2 Antioxidant Pathway. J. Neuroinflammation 14 (1), 119. 10.1186/s12974-017-0895-5 28610608 PMC5470182

[B46] Zhang L. Cui M. Chen S. (2020). Identification of the Molecular Mechanisms of Peimine in the Treatment of Cough Using Computational Target Fishing. Molecules 25 (5), 1105. 10.3390/molecules25051105 PMC7179178 32131410

[B47] Zhao C. Gu Y. Zeng X. Wang J. (2018). NLRP3 Inflammasome Regulates Th17 Differentiation in Rheumatoid Arthritis. Clin. Immunol. 197, 154–160. 10.1016/j.clim.2018.09.007 30236770

[B48] Zhou M. Jiao L. Liu Y. (2019). sFRP2 Promotes Airway Inflammation and Th17/Treg Imbalance in COPD via Wnt/β-Catenin Pathway. Respir. Physiol. Neurobiol. 270, 103282. 10.1016/j.resp.2019.103282 31430541

[B49] Zhou S. Ai Z. Li W. You P. Wu C. Li L. (2020). Deciphering the Pharmacological Mechanisms of Taohe-Chengqi Decoction Extract against Renal Fibrosis through Integrating Network Pharmacology and Experimental Validation *In Vitro* and *In Vivo* . Front. Pharmacol. 11, 425. 10.3389/fphar.2020.00425 32372953 PMC7176980

