# Supplementary material for: Yanghe Decoction Suppresses the Experimental Autoimmune Thyroiditis in Rats by Improving NLRP3 Inflammasome and Immune Dysregulation
Source: Front Pharmacol. 2021 Jun 21;12:645354. doi: 10.3389/fphar.2021.645354 (PMC8255388; doi:10.3389/fphar.2021.645354)
Supplement: Supplementary file 1 [file DataSheet1.pdf]

Supplementary Table1

Compound	CAS ID	Canonical SMILES	Target	Probability
2-Hexaprenyl-6-methoxy-1,4-benzoquinone	—	CC(=CCCC(=CCCC(=CCCC(=CCCC(=CCCC(=CCC1=CC(=O)C=C(C1=O)OC)C)C)C)C)C)C	ALOX5	0.151273102
2-Hexaprenyl-6-methoxy-1,4-benzoquinone	—	CC(=CCCC(=CCCC(=CCCC(=CCCC(=CCCC(=CCC1=CC(=O)C=C(C1=O)OC)C)C)C)C)C)C	MPEG1	0.086885855
4-Amino-1-piperidinecarboxylic acid	959317-82-9	C1CN(CCC1N)C(=O)O	CHRNA4	0.064951087
4-Amino-1-piperidinecarboxylic acid	959317-82-9	C1CN(CCC1N)C(=O)O	OAT	0.054487947
4-Amino-1-piperidinecarboxylic acid	959317-82-9	C1CN(CCC1N)C(=O)O	ENPEP	0.044308319
4-Amino-1-piperidinecarboxylic acid	959317-82-9	C1CN(CCC1N)C(=O)O	SLC1A2	0.044308319
4-Amino-1-piperidinecarboxylic acid	959317-82-9	C1CN(CCC1N)C(=O)O	PEPD	0.044308319
4-Amino-1-piperidinecarboxylic acid	959317-82-9	C1CN(CCC1N)C(=O)O	CPB2	0.03397069
4-Amino-1-piperidinecarboxylic acid	959317-82-9	C1CN(CCC1N)C(=O)O	PLG	0.03397069
4-Amino-1-piperidinecarboxylic acid	959317-82-9	C1CN(CCC1N)C(=O)O	CPN1	0.023832743
4-Amino-1-piperidinecarboxylic acid	959317-82-9	C1CN(CCC1N)C(=O)O	CHRNA3	0.023832743
4-Amino-1-piperidinecarboxylic acid	959317-82-9	C1CN(CCC1N)C(=O)O	CHRNA7	0.023832743
4-Amino-1-piperidinecarboxylic acid	959317-82-9	C1CN(CCC1N)C(=O)O	ANPEP	0.023832743
4-Amino-1-piperidinecarboxylic acid	959317-82-9	C1CN(CCC1N)C(=O)O	GABBR2	0.023832743
4-Amino-1-piperidinecarboxylic acid	959317-82-9	C1CN(CCC1N)C(=O)O	CACNA2D1	0.023832743
4-Amino-1-piperidinecarboxylic acid	959317-82-9	C1CN(CCC1N)C(=O)O	SLC6A12	0.023832743
4-Amino-1-piperidinecarboxylic acid	959317-82-9	C1CN(CCC1N)C(=O)O	ADORA3	0.023832743
4-Amino-1-piperidinecarboxylic acid	959317-82-9	C1CN(CCC1N)C(=O)O	CHRNB2	0.064951087
4-Amino-1-piperidinecarboxylic acid	959317-82-9	C1CN(CCC1N)C(=O)O	CHRNB4	0.023832743
4-Amino-1-piperidinecarboxylic acid	959317-82-9	C1CN(CCC1N)C(=O)O	GABBR1	0.023832743
5-Megastigmen-7-yne-3,9-diol 3-glucoside	62512-27-0	CC1=C(C(CC(C1)OC2C(C(C(C(O2)CO)O)O)O)(C)C)C#CC(C)O	SLC5A2	0.109339753

5-Megastigmen-7-yne-3,9-diol 3-glucoside	62512-27-0	CC1=C(C(CC(C1)OC2C(C(C(C(O2)CO)O)O)O)(C)C)C#CC(C)O	SLC5A1	0.109339753
5-Megastigmen-7-yne-3,9-diol 3-glucoside	62512-27-0	CC1=C(C(CC(C1)OC2C(C(C(C(O2)CO)O)O)O)(C)C)C#CC(C)O	TYR	0.109339753
5-Megastigmen-7-yne-3,9-diol 3-glucoside	62512-27-0	CC1=C(C(CC(C1)OC2C(C(C(C(O2)CO)O)O)O)(C)C)C#CC(C)O	ADORA1	0.109339753
5-Megastigmen-7-yne-3,9-diol 3-glucoside	62512-27-0	CC1=C(C(CC(C1)OC2C(C(C(C(O2)CO)O)O)O)(C)C)C#CC(C)O	ADORA2A	0.109339753
5-Megastigmen-7-yne-3,9-diol 3-glucoside	62512-27-0	CC1=C(C(CC(C1)OC2C(C(C(C(O2)CO)O)O)O)(C)C)C#CC(C)O	IL2	0.109339753
5-Megastigmen-7-yne-3,9-diol 3-glucoside	62512-27-0	CC1=C(C(CC(C1)OC2C(C(C(C(O2)CO)O)O)O)(C)C)C#CC(C)O	CA2	0.109339753
5-Megastigmen-7-yne-3,9-diol 3-glucoside	62512-27-0	CC1=C(C(CC(C1)OC2C(C(C(C(O2)CO)O)O)O)(C)C)C#CC(C)O	CA1	0.109339753
5-Megastigmen-7-yne-3,9-diol 3-glucoside	62512-27-0	CC1=C(C(CC(C1)OC2C(C(C(C(O2)CO)O)O)O)(C)C)C#CC(C)O	F3	0.109339753
5-Megastigmen-7-yne-3,9-diol 3-glucoside	62512-27-0	CC1=C(C(CC(C1)OC2C(C(C(C(O2)CO)O)O)O)(C)C)C#CC(C)O	HRAS	0.109339753
5-Megastigmen-7-yne-3,9-diol 3-glucoside	62512-27-0	CC1=C(C(CC(C1)OC2C(C(C(C(O2)CO)O)O)O)(C)C)C#CC(C)O	ADORA3	0.109339753
5-Megastigmen-7-yne-3,9-diol 3-glucoside	62512-27-0	CC1=C(C(CC(C1)OC2C(C(C(C(O2)CO)O)O)O)(C)C)C#CC(C)O	IGFBP3	0.109339753
5-Megastigmen-7-yne-3,9-diol 3-glucoside	62512-27-0	CC1=C(C(CC(C1)OC2C(C(C(C(O2)CO)O)O)O)(C)C)C#CC(C)O	CA12	0.109339753
5-Megastigmen-7-yne-3,9-diol 3-glucoside	62512-27-0	CC1=C(C(CC(C1)OC2C(C(C(C(O2)CO)O)O)O)(C)C)C#CC(C)O	CA14	0.109339753
5-Megastigmen-7-yne-3,9-diol 3-glucoside	62512-27-0	CC1=C(C(CC(C1)OC2C(C(C(C(O2)CO)O)O)O)(C)C)C#CC(C)O	CA9	0.109339753
5-Megastigmen-7-yne-3,9-diol 3-glucoside	62512-27-0	CC1=C(C(CC(C1)OC2C(C(C(C(O2)CO)O)O)O)(C)C)C#CC(C)O	SLC29A1	0.109339753
5-Megastigmen-7-yne-3,9-diol 3-glucoside	62512-27-0	CC1=C(C(CC(C1)OC2C(C(C(C(O2)CO)O)O)O)(C)C)C#CC(C)O	EPHX2	0.109339753
Corchoionoside B	189344-54-5	CC1=CC(=O)CC(C1(C=CC(=O)COC2C(C(C(C(O2)CO)O)O)O)O)(C)C	HSD11B2	0.115736675
Corchoionoside B	189344-54-5	CC1=CC(=O)CC(C1(C=CC(=O)COC2C(C(C(C(O2)CO)O)O)O)O)(C)C	PTPN1	0.115736675
Corchoionoside B	189344-54-5	CC1=CC(=O)CC(C1(C=CC(=O)COC2C(C(C(C(O2)CO)O)O)O)O)(C)C	SLC5A2	0.115736675
Corchoionoside B	189344-54-5	CC1=CC(=O)CC(C1(C=CC(=O)COC2C(C(C(C(O2)CO)O)O)O)O)(C)C	CA2	0.115736675
Corchoionoside B	189344-54-5	CC1=CC(=O)CC(C1(C=CC(=O)COC2C(C(C(C(O2)CO)O)O)O)O)(C)C	CA1	0.115736675
Corchoionoside B	189344-54-5	CC1=CC(=O)CC(C1(C=CC(=O)COC2C(C(C(C(O2)CO)O)O)O)O)(C)C	CA12	0.115736675

Corchoionoside B	189344-54-5	CC1=CC(=O)CC(C1(C=CC(=O)COC2C(C(C(C(O2)CO)O)O)O)O)(C)C	CA9	0.115736675
Corchoionoside B	189344-54-5	CC1=CC(=O)CC(C1(C=CC(=O)COC2C(C(C(C(O2)CO)O)O)O)O)(C)C	ADORA3	0.115736675
Corchoionoside B	189344-54-5	CC1=CC(=O)CC(C1(C=CC(=O)COC2C(C(C(C(O2)CO)O)O)O)O)(C)C	MMP9	0.115736675
Corchoionoside B	189344-54-5	CC1=CC(=O)CC(C1(C=CC(=O)COC2C(C(C(C(O2)CO)O)O)O)O)(C)C	SLC5A1	0.115736675
Corchoionoside B	189344-54-5	CC1=CC(=O)CC(C1(C=CC(=O)COC2C(C(C(C(O2)CO)O)O)O)O)(C)C	ADORA2A	0.115736675
Corchoionoside B	189344-54-5	CC1=CC(=O)CC(C1(C=CC(=O)COC2C(C(C(C(O2)CO)O)O)O)O)(C)C	TYR	0.115736675
Corchoionoside B	189344-54-5	CC1=CC(=O)CC(C1(C=CC(=O)COC2C(C(C(C(O2)CO)O)O)O)O)(C)C	OGA	0.115736675
Corchoionoside B	189344-54-5	CC1=CC(=O)CC(C1(C=CC(=O)COC2C(C(C(C(O2)CO)O)O)O)O)(C)C	HSD11B1	0.115736675
Corchoionoside B	189344-54-5	CC1=CC(=O)CC(C1(C=CC(=O)COC2C(C(C(C(O2)CO)O)O)O)O)(C)C	SLC29A1	0.115736675
Corchoionoside B	189344-54-5	CC1=CC(=O)CC(C1(C=CC(=O)COC2C(C(C(C(O2)CO)O)O)O)O)(C)C	CA14	0.115736675
Corchoionoside B	189344-54-5	CC1=CC(=O)CC(C1(C=CC(=O)COC2C(C(C(C(O2)CO)O)O)O)O)(C)C	PPP1CC	0.115736675
Corchoionoside B	189344-54-5	CC1=CC(=O)CC(C1(C=CC(=O)COC2C(C(C(C(O2)CO)O)O)O)O)(C)C	PPP2CA	0.115736675
Corchoionoside B	189344-54-5	CC1=CC(=O)CC(C1(C=CC(=O)COC2C(C(C(C(O2)CO)O)O)O)O)(C)C	PPP2R5A	0.115736675
Corchoionoside B	189344-54-5	CC1=CC(=O)CC(C1(C=CC(=O)COC2C(C(C(C(O2)CO)O)O)O)O)(C)C	IL2	0.115736675
Corchoionoside B	189344-54-5	CC1=CC(=O)CC(C1(C=CC(=O)COC2C(C(C(C(O2)CO)O)O)O)O)(C)C	ADA	0.115736675
Corchoionoside B	189344-54-5	CC1=CC(=O)CC(C1(C=CC(=O)COC2C(C(C(C(O2)CO)O)O)O)O)(C)C	HDAC1	0.115736675
Corchoionoside B	189344-54-5	CC1=CC(=O)CC(C1(C=CC(=O)COC2C(C(C(C(O2)CO)O)O)O)O)(C)C	GLI1	0.115736675
Corchoionoside B	189344-54-5	CC1=CC(=O)CC(C1(C=CC(=O)COC2C(C(C(C(O2)CO)O)O)O)O)(C)C	GBA	0.115736675
Corchoionoside B	189344-54-5	CC1=CC(=O)CC(C1(C=CC(=O)COC2C(C(C(C(O2)CO)O)O)O)O)(C)C	FUCA1	0.115736675
Corchoionoside B	189344-54-5	CC1=CC(=O)CC(C1(C=CC(=O)COC2C(C(C(C(O2)CO)O)O)O)O)(C)C	MME	0.115736675
Corchoionoside B	189344-54-5	CC1=CC(=O)CC(C1(C=CC(=O)COC2C(C(C(C(O2)CO)O)O)O)O)(C)C	MMP13	0.115736675
Corchoionoside B	189344-54-5	CC1=CC(=O)CC(C1(C=CC(=O)COC2C(C(C(C(O2)CO)O)O)O)O)(C)C	MMP1	0.115736675

Corchoionoside B	189344-54-5	CC1=CC(=O)CC(C1(C=CC(=O)COC2C(C(C(C(O2)CO)O)O)O)O)(C)C	MMP7	0.115736675
Corchoionoside B	189344-54-5	CC1=CC(=O)CC(C1(C=CC(=O)COC2C(C(C(C(O2)CO)O)O)O)O)(C)C	MMP12	0.115736675
Corchoionoside B	189344-54-5	CC1=CC(=O)CC(C1(C=CC(=O)COC2C(C(C(C(O2)CO)O)O)O)O)(C)C	MMP8	0.115736675
Corchoionoside B	189344-54-5	CC1=CC(=O)CC(C1(C=CC(=O)COC2C(C(C(C(O2)CO)O)O)O)O)(C)C	CDK2	0.115736675
Corchoionoside B	189344-54-5	CC1=CC(=O)CC(C1(C=CC(=O)COC2C(C(C(C(O2)CO)O)O)O)O)(C)C	ADK	0.115736675
Corchoionoside B	189344-54-5	CC1=CC(=O)CC(C1(C=CC(=O)COC2C(C(C(C(O2)CO)O)O)O)O)(C)C	EGFR	0.115736675
Corchoionoside B	189344-54-5	CC1=CC(=O)CC(C1(C=CC(=O)COC2C(C(C(C(O2)CO)O)O)O)O)(C)C	PTPN11	0.115736675
Corchoionoside B	189344-54-5	CC1=CC(=O)CC(C1(C=CC(=O)COC2C(C(C(C(O2)CO)O)O)O)O)(C)C	CA7	0.115736675
Corchoionoside B	189344-54-5	CC1=CC(=O)CC(C1(C=CC(=O)COC2C(C(C(C(O2)CO)O)O)O)O)(C)C	NEU3	0.115736675
Corchoionoside B	189344-54-5	CC1=CC(=O)CC(C1(C=CC(=O)COC2C(C(C(C(O2)CO)O)O)O)O)(C)C	CA13	0.115736675
Corchoionoside B	189344-54-5	CC1=CC(=O)CC(C1(C=CC(=O)COC2C(C(C(C(O2)CO)O)O)O)O)(C)C	NEU4	0.115736675
Corchoionoside B	189344-54-5	CC1=CC(=O)CC(C1(C=CC(=O)COC2C(C(C(C(O2)CO)O)O)O)O)(C)C	GAA	0.115736675
Corchoionoside B	189344-54-5	CC1=CC(=O)CC(C1(C=CC(=O)COC2C(C(C(C(O2)CO)O)O)O)O)(C)C	HK2	0.115736675
Corchoionoside B	189344-54-5	CC1=CC(=O)CC(C1(C=CC(=O)COC2C(C(C(C(O2)CO)O)O)O)O)(C)C	HK1	0.115736675
Corchoionoside B	189344-54-5	CC1=CC(=O)CC(C1(C=CC(=O)COC2C(C(C(C(O2)CO)O)O)O)O)(C)C	MANBA	0.115736675
Corchoionoside B	189344-54-5	CC1=CC(=O)CC(C1(C=CC(=O)COC2C(C(C(C(O2)CO)O)O)O)O)(C)C	HSPA8	0.115736675
Corchoionoside B	189344-54-5	CC1=CC(=O)CC(C1(C=CC(=O)COC2C(C(C(C(O2)CO)O)O)O)O)(C)C	MAPK10	0.115736675
Corchoionoside B	189344-54-5	CC1=CC(=O)CC(C1(C=CC(=O)COC2C(C(C(C(O2)CO)O)O)O)O)(C)C	HRAS	0.115736675
Corchoionoside B	189344-54-5	CC1=CC(=O)CC(C1(C=CC(=O)COC2C(C(C(C(O2)CO)O)O)O)O)(C)C	LGALS3	0.115736675
Corchoionoside B	189344-54-5	CC1=CC(=O)CC(C1(C=CC(=O)COC2C(C(C(C(O2)CO)O)O)O)O)(C)C	LGALS9	0.115736675
Corchoionoside B	189344-54-5	CC1=CC(=O)CC(C1(C=CC(=O)COC2C(C(C(C(O2)CO)O)O)O)O)(C)C	MMP3	0.115736675
Corchoionoside B	189344-54-5	CC1=CC(=O)CC(C1(C=CC(=O)COC2C(C(C(C(O2)CO)O)O)O)O)(C)C	ADAM17	0.115736675

Corchoionoside B	189344-54-5	CC1=CC(=O)CC(C1(C=CC(=O)COC2C(C(C(C(O2)CO)O)O)O)O)(C)C	AKR1B1	0.115736675
Corchoionoside B	189344-54-5	CC1=CC(=O)CC(C1(C=CC(=O)COC2C(C(C(C(O2)CO)O)O)O)O)(C)C	CDA	0.115736675
Corchoionoside B	189344-54-5	CC1=CC(=O)CC(C1(C=CC(=O)COC2C(C(C(C(O2)CO)O)O)O)O)(C)C	ERN1	0.115736675
Corchoionoside B	189344-54-5	CC1=CC(=O)CC(C1(C=CC(=O)COC2C(C(C(C(O2)CO)O)O)O)O)(C)C	TYMP	0.115736675
Corchoionoside B	189344-54-5	CC1=CC(=O)CC(C1(C=CC(=O)COC2C(C(C(C(O2)CO)O)O)O)O)(C)C	TOP1	0.115736675
Corchoionoside B	189344-54-5	CC1=CC(=O)CC(C1(C=CC(=O)COC2C(C(C(C(O2)CO)O)O)O)O)(C)C	FLT1	0.115736675
Corchoionoside B	189344-54-5	CC1=CC(=O)CC(C1(C=CC(=O)COC2C(C(C(C(O2)CO)O)O)O)O)(C)C	KIT	0.115736675
Corchoionoside B	189344-54-5	CC1=CC(=O)CC(C1(C=CC(=O)COC2C(C(C(C(O2)CO)O)O)O)O)(C)C	GAPDH	0.115736675
Corchoionoside B	189344-54-5	CC1=CC(=O)CC(C1(C=CC(=O)COC2C(C(C(C(O2)CO)O)O)O)O)(C)C	PNP	0.115736675
Corchoionoside B	189344-54-5	CC1=CC(=O)CC(C1(C=CC(=O)COC2C(C(C(C(O2)CO)O)O)O)O)(C)C	SLC28A2	0.115736675
Corchoionoside B	189344-54-5	CC1=CC(=O)CC(C1(C=CC(=O)COC2C(C(C(C(O2)CO)O)O)O)O)(C)C	SLC5A4	0.115736675
Corchoionoside B	189344-54-5	CC1=CC(=O)CC(C1(C=CC(=O)COC2C(C(C(C(O2)CO)O)O)O)O)(C)C	CDK1	0.115736675
Corchoionoside B	189344-54-5	CC1=CC(=O)CC(C1(C=CC(=O)COC2C(C(C(C(O2)CO)O)O)O)O)(C)C	TK1	0.115736675
Corchoionoside B	189344-54-5	CC1=CC(=O)CC(C1(C=CC(=O)COC2C(C(C(C(O2)CO)O)O)O)O)(C)C	CA6	0.115736675
Corchoionoside B	189344-54-5	CC1=CC(=O)CC(C1(C=CC(=O)COC2C(C(C(C(O2)CO)O)O)O)O)(C)C	CA4	0.115736675
Corchoionoside B	189344-54-5	CC1=CC(=O)CC(C1(C=CC(=O)COC2C(C(C(C(O2)CO)O)O)O)O)(C)C	CA5A	0.115736675
Corchoionoside B	189344-54-5	CC1=CC(=O)CC(C1(C=CC(=O)COC2C(C(C(C(O2)CO)O)O)O)O)(C)C	KDM4C	0.115736675
Corchoionoside B	189344-54-5	CC1=CC(=O)CC(C1(C=CC(=O)COC2C(C(C(C(O2)CO)O)O)O)O)(C)C	F2	0.115736675
Corchoionoside B	189344-54-5	CC1=CC(=O)CC(C1(C=CC(=O)COC2C(C(C(C(O2)CO)O)O)O)O)(C)C	CDK9	0.115736675
Corchoionoside B	189344-54-5	CC1=CC(=O)CC(C1(C=CC(=O)COC2C(C(C(C(O2)CO)O)O)O)O)(C)C	CSNK2A1	0.115736675
Corchoionoside B	189344-54-5	CC1=CC(=O)CC(C1(C=CC(=O)COC2C(C(C(C(O2)CO)O)O)O)O)(C)C	HSPA5	0.115736675
Corchoionoside B	189344-54-5	CC1=CC(=O)CC(C1(C=CC(=O)COC2C(C(C(C(O2)CO)O)O)O)O)(C)C	IGFBP3	0.115736675

Corchoionoside B	189344-54-5	CC1=CC(=O)CC(C1(C=CC(=O)COC2C(C(C(C(O2)CO)O)O)O)O)(C)C	FOLH1	0.115736675
Corchoionoside B	189344-54-5	CC1=CC(=O)CC(C1(C=CC(=O)COC2C(C(C(C(O2)CO)O)O)O)O)(C)C	AMD1	0.115736675
Corchoionoside B	189344-54-5	CC1=CC(=O)CC(C1(C=CC(=O)COC2C(C(C(C(O2)CO)O)O)O)O)(C)C	ADORA1	0.115736675
Corchoionoside B	189344-54-5	CC1=CC(=O)CC(C1(C=CC(=O)COC2C(C(C(C(O2)CO)O)O)O)O)(C)C	VARS	0.115736675
Corchoionoside B	189344-54-5	CC1=CC(=O)CC(C1(C=CC(=O)COC2C(C(C(C(O2)CO)O)O)O)O)(C)C	LARS	0.115736675
Corchoionoside B	189344-54-5	CC1=CC(=O)CC(C1(C=CC(=O)COC2C(C(C(C(O2)CO)O)O)O)O)(C)C	MGAM	0.115736675
Corchoionoside B	189344-54-5	CC1=CC(=O)CC(C1(C=CC(=O)COC2C(C(C(C(O2)CO)O)O)O)O)(C)C	MAG	0.115736675
Corchoionoside B	189344-54-5	CC1=CC(=O)CC(C1(C=CC(=O)COC2C(C(C(C(O2)CO)O)O)O)O)(C)C	CXCR2	0.115736675
Corchoionoside B	189344-54-5	CC1=CC(=O)CC(C1(C=CC(=O)COC2C(C(C(C(O2)CO)O)O)O)O)(C)C	CCNA1	0.115736675
Corchoionoside B	189344-54-5	CC1=CC(=O)CC(C1(C=CC(=O)COC2C(C(C(C(O2)CO)O)O)O)O)(C)C	CCNA2	0.115736675
Corchoionoside B	189344-54-5	CC1=CC(=O)CC(C1(C=CC(=O)COC2C(C(C(C(O2)CO)O)O)O)O)(C)C	CCNT1	0.115736675
Ilicifolinoside A	—	CC(=CCO)COC1C(C(C(C(O1)CO)O)O)O	CDA	0.053556076
Ilicifolinoside A	—	CC(=CCO)COC1C(C(C(C(O1)CO)O)O)O	PYGM	0.053556076
Ilicifolinoside A	—	CC(=CCO)COC1C(C(C(C(O1)CO)O)O)O	VEGFA	0.053556076
Ilicifolinoside A	—	CC(=CCO)COC1C(C(C(C(O1)CO)O)O)O	FGF1	0.053556076
Ilicifolinoside A	—	CC(=CCO)COC1C(C(C(C(O1)CO)O)O)O	FGF2	0.053556076
Ilicifolinoside A	—	CC(=CCO)COC1C(C(C(C(O1)CO)O)O)O	HPSE	0.053556076
Ilicifolinoside A	—	CC(=CCO)COC1C(C(C(C(O1)CO)O)O)O	ADA	0.053556076
Ilicifolinoside A	—	CC(=CCO)COC1C(C(C(C(O1)CO)O)O)O	MGAM	0.053556076
Ilicifolinoside A	—	CC(=CCO)COC1C(C(C(C(O1)CO)O)O)O	ADORA1	0.053556076
Ilicifolinoside A	—	CC(=CCO)COC1C(C(C(C(O1)CO)O)O)O	ADORA2A	0.053556076
Ilicifolinoside A	—	CC(=CCO)COC1C(C(C(C(O1)CO)O)O)O	FOLH1	0.053556076
Ilicifolinoside A	—	CC(=CCO)COC1C(C(C(C(O1)CO)O)O)O	PSEN2	0.053556076
Ilicifolinoside A	—	CC(=CCO)COC1C(C(C(C(O1)CO)O)O)O	SI	0.053556076
Ilicifolinoside A	—	CC(=CCO)COC1C(C(C(C(O1)CO)O)O)O	TREH	0.053556076
Ilicifolinoside A	—	CC(=CCO)COC1C(C(C(C(O1)CO)O)O)O	CDK1	0.053556076
Ilicifolinoside A	—	CC(=CCO)COC1C(C(C(C(O1)CO)O)O)O	PNP	0.053556076
Ilicifolinoside A	—	CC(=CCO)COC1C(C(C(C(O1)CO)O)O)O	ADORA3	0.053556076
Ilicifolinoside A	—	CC(=CCO)COC1C(C(C(C(O1)CO)O)O)O	ADK	0.053556076
Ilicifolinoside A	—	CC(=CCO)COC1C(C(C(C(O1)CO)O)O)O	AMY2A	0.053556076
Ilicifolinoside A	—	CC(=CCO)COC1C(C(C(C(O1)CO)O)O)O	GGH	0.053556076
Ilicifolinoside A	—	CC(=CCO)COC1C(C(C(C(O1)CO)O)O)O	PIN1	0.053556076

Ilicifolinoside A	—	CC(=CCO)COC1C(C(C(C(O1)CO)O)O)O	DPP4	0.053556076
Ilicifolinoside A	—	CC(=CCO)COC1C(C(C(C(O1)CO)O)O)O	HSPA8	0.053556076
Ilicifolinoside A	—	CC(=CCO)COC1C(C(C(C(O1)CO)O)O)O	HSPA5	0.053556076
Ilicifolinoside A	—	CC(=CCO)COC1C(C(C(C(O1)CO)O)O)O	OGA	0.053556076
Ilicifolinoside A	—	CC(=CCO)COC1C(C(C(C(O1)CO)O)O)O	NAALAD2	0.053556076
Ilicifolinoside A	—	CC(=CCO)COC1C(C(C(C(O1)CO)O)O)O	LGALS4	0.053556076
Ilicifolinoside A	—	CC(=CCO)COC1C(C(C(C(O1)CO)O)O)O	LGALS8	0.053556076
Ilicifolinoside A	—	CC(=CCO)COC1C(C(C(C(O1)CO)O)O)O	CDK2	0.053556076
Ilicifolinoside A	—	CC(=CCO)COC1C(C(C(C(O1)CO)O)O)O	PSENEN	0.053556076
Ilicifolinoside A	—	CC(=CCO)COC1C(C(C(C(O1)CO)O)O)O	NCSTN	0.053556076
Ilicifolinoside A	—	CC(=CCO)COC1C(C(C(C(O1)CO)O)O)O	APH1A	0.053556076
Ilicifolinoside A	—	CC(=CCO)COC1C(C(C(C(O1)CO)O)O)O	PSEN1	0.053556076
Ilicifolinoside A	—	CC(=CCO)COC1C(C(C(C(O1)CO)O)O)O	APH1B	0.053556076
Ilicifolinoside A	—	CC(=CCO)COC1C(C(C(C(O1)CO)O)O)O	CCNA1	0.053556076
Ilicifolinoside A	—	CC(=CCO)COC1C(C(C(C(O1)CO)O)O)O	CCNA2	0.053556076
Moclobemide	71320-77-9	C1COCCN1CCNC(=O)C2=CC=C(C=C2)Cl	MAOA	1
Moclobemide	71320-77-9	C1COCCN1CCNC(=O)C2=CC=C(C=C2)Cl	MAOB	1
Moclobemide	71320-77-9	C1COCCN1CCNC(=O)C2=CC=C(C=C2)Cl	HTR1A	1
Moclobemide	71320-77-9	C1COCCN1CCNC(=O)C2=CC=C(C=C2)Cl	AKR1C3	0.062621967
Moclobemide	71320-77-9	C1COCCN1CCNC(=O)C2=CC=C(C=C2)Cl	HRH3	0.062621967
Moclobemide	71320-77-9	C1COCCN1CCNC(=O)C2=CC=C(C=C2)Cl	HPGDS	0.053556076
Moclobemide	71320-77-9	C1COCCN1CCNC(=O)C2=CC=C(C=C2)Cl	KCNA5	0.053556076
Moclobemide	71320-77-9	C1COCCN1CCNC(=O)C2=CC=C(C=C2)Cl	EPHX2	0.053556076
Moclobemide	71320-77-9	C1COCCN1CCNC(=O)C2=CC=C(C=C2)Cl	PARP1	0.053556076
Moclobemide	71320-77-9	C1COCCN1CCNC(=O)C2=CC=C(C=C2)Cl	AR	0.053556076
Moclobemide	71320-77-9	C1COCCN1CCNC(=O)C2=CC=C(C=C2)Cl	PRKDC	0.053556076
Moclobemide	71320-77-9	C1COCCN1CCNC(=O)C2=CC=C(C=C2)Cl	P2RX7	0.053556076
Moclobemide	71320-77-9	C1COCCN1CCNC(=O)C2=CC=C(C=C2)Cl	CYP17A1	0.053556076
Moclobemide	71320-77-9	C1COCCN1CCNC(=O)C2=CC=C(C=C2)Cl	WNT3A	0.053556076
Moclobemide	71320-7	C1COCCN1CCNC(=O)C2=CC=C(C=C2)Cl	HSD11B1	0.053556076

	7-9			
Moclobemide	71320-77-9	C1COCCN1CCNC(=O)C2=CC=C(C=C2)Cl	PIM1	0.053556076
Moclobemide	71320-77-9	C1COCCN1CCNC(=O)C2=CC=C(C=C2)Cl	PIK3CB	0.053556076
Moclobemide	71320-77-9	C1COCCN1CCNC(=O)C2=CC=C(C=C2)Cl	PIM2	0.053556076
Moclobemide	71320-77-9	C1COCCN1CCNC(=O)C2=CC=C(C=C2)Cl	ALPL	0.053556076
Moclobemide	71320-77-9	C1COCCN1CCNC(=O)C2=CC=C(C=C2)Cl	DUSP3	0.053556076
Moclobemide	71320-77-9	C1COCCN1CCNC(=O)C2=CC=C(C=C2)Cl	PTPN1	0.053556076
Moclobemide	71320-77-9	C1COCCN1CCNC(=O)C2=CC=C(C=C2)Cl	CDC25B	0.053556076
Moclobemide	71320-77-9	C1COCCN1CCNC(=O)C2=CC=C(C=C2)Cl	HRH1	0.053556076
Moclobemide	71320-77-9	C1COCCN1CCNC(=O)C2=CC=C(C=C2)Cl	BRD3	0.053556076
Moclobemide	71320-77-9	C1COCCN1CCNC(=O)C2=CC=C(C=C2)Cl	DYRK1A	0.053556076
Moclobemide	71320-77-9	C1COCCN1CCNC(=O)C2=CC=C(C=C2)Cl	CLK4	0.053556076
Moclobemide	71320-77-9	C1COCCN1CCNC(=O)C2=CC=C(C=C2)Cl	MKNK1	0.053556076
Moclobemide	71320-77-9	C1COCCN1CCNC(=O)C2=CC=C(C=C2)Cl	PDE9A	0.053556076
Moclobemide	71320-77-9	C1COCCN1CCNC(=O)C2=CC=C(C=C2)Cl	PDE1C	0.053556076
Moclobemide	71320-77-9	C1COCCN1CCNC(=O)C2=CC=C(C=C2)Cl	RAD51	0.053556076
Moclobemide	71320-77-9	C1COCCN1CCNC(=O)C2=CC=C(C=C2)Cl	RAD1	0.053556076
Moclobemide	71320-77-9	C1COCCN1CCNC(=O)C2=CC=C(C=C2)Cl	BCL2A1	0.053556076
Moclobemide	71320-77-9	C1COCCN1CCNC(=O)C2=CC=C(C=C2)Cl	PIK3CD	0.053556076
Moclobemide	71320-77-9	C1COCCN1CCNC(=O)C2=CC=C(C=C2)Cl	CD38	0.053556076
Moclobemide	71320-77-9	C1COCCN1CCNC(=O)C2=CC=C(C=C2)Cl	LIPE	0.053556076
Moclobemide	71320-77-9	C1COCCN1CCNC(=O)C2=CC=C(C=C2)Cl	PDE10A	0.053556076
Moclobemide	71320-7	C1COCCN1CCNC(=O)C2=CC=C(C=C2)Cl	ADORA1	0.053556076

	7-9			
Moclobemide	71320-77-9	C1COCCN1CCNC(=O)C2=CC=C(C=C2)Cl	ADORA2A	0.053556076
Moclobemide	71320-77-9	C1COCCN1CCNC(=O)C2=CC=C(C=C2)Cl	ADORA2B	0.053556076
Moclobemide	71320-77-9	C1COCCN1CCNC(=O)C2=CC=C(C=C2)Cl	PDE4B	0.053556076
Moclobemide	71320-77-9	C1COCCN1CCNC(=O)C2=CC=C(C=C2)Cl	PRKCZ	0.053556076
Moclobemide	71320-77-9	C1COCCN1CCNC(=O)C2=CC=C(C=C2)Cl	PRMT3	0.053556076
Moclobemide	71320-77-9	C1COCCN1CCNC(=O)C2=CC=C(C=C2)Cl	EDNRB	0.053556076
Moclobemide	71320-77-9	C1COCCN1CCNC(=O)C2=CC=C(C=C2)Cl	EDNRA	0.053556076
Moclobemide	71320-77-9	C1COCCN1CCNC(=O)C2=CC=C(C=C2)Cl	ATM	0.053556076
Moclobemide	71320-77-9	C1COCCN1CCNC(=O)C2=CC=C(C=C2)Cl	CFTR	0.053556076
Moclobemide	71320-77-9	C1COCCN1CCNC(=O)C2=CC=C(C=C2)Cl	TBXAS1	0.053556076
Moclobemide	71320-77-9	C1COCCN1CCNC(=O)C2=CC=C(C=C2)Cl	GSK3B	0.053556076
Moclobemide	71320-77-9	C1COCCN1CCNC(=O)C2=CC=C(C=C2)Cl	CSNK1D	0.053556076
Moclobemide	71320-77-9	C1COCCN1CCNC(=O)C2=CC=C(C=C2)Cl	MMP12	0.053556076
Moclobemide	71320-77-9	C1COCCN1CCNC(=O)C2=CC=C(C=C2)Cl	CSNK1E	0.053556076
Moclobemide	71320-77-9	C1COCCN1CCNC(=O)C2=CC=C(C=C2)Cl	CCR9	0.053556076
Moclobemide	71320-77-9	C1COCCN1CCNC(=O)C2=CC=C(C=C2)Cl	RPS6KB1	0.053556076
Moclobemide	71320-77-9	C1COCCN1CCNC(=O)C2=CC=C(C=C2)Cl	LSS	0.053556076
Moclobemide	71320-77-9	C1COCCN1CCNC(=O)C2=CC=C(C=C2)Cl	PDE4D	0.053556076
Moclobemide	71320-77-9	C1COCCN1CCNC(=O)C2=CC=C(C=C2)Cl	BRD4	0.053556076
Moclobemide	71320-77-9	C1COCCN1CCNC(=O)C2=CC=C(C=C2)Cl	TYMS	0.053556076
Moclobemide	71320-77-9	C1COCCN1CCNC(=O)C2=CC=C(C=C2)Cl	LTA4H	0.053556076
Moclobemide	71320-7	C1COCCN1CCNC(=O)C2=CC=C(C=C2)Cl	ECE1	0.053556076

	7-9			
Moclobemide	71320-77-9	C1COCCN1CCNC(=O)C2=CC=C(C=C2)Cl	MAPKAPK2	0.053556076
Moclobemide	71320-77-9	C1COCCN1CCNC(=O)C2=CC=C(C=C2)Cl	PFKFB3	0.053556076
Moclobemide	71320-77-9	C1COCCN1CCNC(=O)C2=CC=C(C=C2)Cl	FFAR1	0.053556076
Moclobemide	71320-77-9	C1COCCN1CCNC(=O)C2=CC=C(C=C2)Cl	KMT5A	0.053556076
Moclobemide	71320-77-9	C1COCCN1CCNC(=O)C2=CC=C(C=C2)Cl	FLT1	0.053556076
Moclobemide	71320-77-9	C1COCCN1CCNC(=O)C2=CC=C(C=C2)Cl	P2RY12	0.053556076
Moclobemide	71320-77-9	C1COCCN1CCNC(=O)C2=CC=C(C=C2)Cl	CDC25C	0.053556076
Moclobemide	71320-77-9	C1COCCN1CCNC(=O)C2=CC=C(C=C2)Cl	KDR	0.053556076
Moclobemide	71320-77-9	C1COCCN1CCNC(=O)C2=CC=C(C=C2)Cl	CDC25A	0.053556076
Moclobemide	71320-77-9	C1COCCN1CCNC(=O)C2=CC=C(C=C2)Cl	AKT1	0.053556076
Moclobemide	71320-77-9	C1COCCN1CCNC(=O)C2=CC=C(C=C2)Cl	CDK1	0.053556076
Moclobemide	71320-77-9	C1COCCN1CCNC(=O)C2=CC=C(C=C2)Cl	PDGFRB	0.053556076
Moclobemide	71320-77-9	C1COCCN1CCNC(=O)C2=CC=C(C=C2)Cl	EGFR	0.053556076
Moclobemide	71320-77-9	C1COCCN1CCNC(=O)C2=CC=C(C=C2)Cl	ESR2	0.053556076
Moclobemide	71320-77-9	C1COCCN1CCNC(=O)C2=CC=C(C=C2)Cl	LDHA	0.053556076
Moclobemide	71320-77-9	C1COCCN1CCNC(=O)C2=CC=C(C=C2)Cl	CDK5	0.053556076
Moclobemide	71320-77-9	C1COCCN1CCNC(=O)C2=CC=C(C=C2)Cl	STS	0.053556076
Moclobemide	71320-77-9	C1COCCN1CCNC(=O)C2=CC=C(C=C2)Cl	BRS3	0.053556076
Moclobemide	71320-77-9	C1COCCN1CCNC(=O)C2=CC=C(C=C2)Cl	BACE1	0.053556076
Moclobemide	71320-77-9	C1COCCN1CCNC(=O)C2=CC=C(C=C2)Cl	CRHR1	0.053556076
Moclobemide	71320-77-9	C1COCCN1CCNC(=O)C2=CC=C(C=C2)Cl	CDK4	0.053556076
Moclobemide	71320-7	C1COCCN1CCNC(=O)C2=CC=C(C=C2)Cl	CDC7	0.053556076

	7-9			
Moclobemide	71320-77-9	C1COCCN1CCNC(=O)C2=CC=C(C=C2)Cl	ESR1	0.053556076
Moclobemide	71320-77-9	C1COCCN1CCNC(=O)C2=CC=C(C=C2)Cl	DDAH1	0.053556076
Moclobemide	71320-77-9	C1COCCN1CCNC(=O)C2=CC=C(C=C2)Cl	MALT1	0.053556076
Moclobemide	71320-77-9	C1COCCN1CCNC(=O)C2=CC=C(C=C2)Cl	GRK2	0.053556076
Moclobemide	71320-77-9	C1COCCN1CCNC(=O)C2=CC=C(C=C2)Cl	GAK	0.053556076
Moclobemide	71320-77-9	C1COCCN1CCNC(=O)C2=CC=C(C=C2)Cl	RPS6KA2	0.053556076
Moclobemide	71320-77-9	C1COCCN1CCNC(=O)C2=CC=C(C=C2)Cl	KCNMA1	0.053556076
Moclobemide	71320-77-9	C1COCCN1CCNC(=O)C2=CC=C(C=C2)Cl	CA2	0.053556076
Moclobemide	71320-77-9	C1COCCN1CCNC(=O)C2=CC=C(C=C2)Cl	CA1	0.053556076
Moclobemide	71320-77-9	C1COCCN1CCNC(=O)C2=CC=C(C=C2)Cl	LRRK2	0.053556076
Moclobemide	71320-77-9	C1COCCN1CCNC(=O)C2=CC=C(C=C2)Cl	BRD2	0.053556076
Moclobemide	71320-77-9	C1COCCN1CCNC(=O)C2=CC=C(C=C2)Cl	PTGFR	0.053556076
Moclobemide	71320-77-9	C1COCCN1CCNC(=O)C2=CC=C(C=C2)Cl	PTGER3	0.053556076
Moclobemide	71320-77-9	C1COCCN1CCNC(=O)C2=CC=C(C=C2)Cl	TDP2	0.053556076
Moclobemide	71320-77-9	C1COCCN1CCNC(=O)C2=CC=C(C=C2)Cl	NOS2	0.053556076
Moclobemide	71320-77-9	C1COCCN1CCNC(=O)C2=CC=C(C=C2)Cl	PDE5A	0.053556076
Moclobemide	71320-77-9	C1COCCN1CCNC(=O)C2=CC=C(C=C2)Cl	PDE3A	0.053556076
Moclobemide	71320-77-9	C1COCCN1CCNC(=O)C2=CC=C(C=C2)Cl	PDE2A	0.053556076
Moclobemide	71320-77-9	C1COCCN1CCNC(=O)C2=CC=C(C=C2)Cl	PDE11A	0.053556076
Moclobemide	71320-77-9	C1COCCN1CCNC(=O)C2=CC=C(C=C2)Cl	CDK5R1	0.053556076
Moclobemide	71320-77-9	C1COCCN1CCNC(=O)C2=CC=C(C=C2)Cl	PFKFB4	0.053556076
Moclobemide	71320-7	C1COCCN1CCNC(=O)C2=CC=C(C=C2)Cl	CCNB1	0.053556076

	7-9			
Molludistin 2"-rhamnoside	—	CC1C(C(C(C(O1)OC2C(C(COC2C3=C(C=C(C4=C3OC(=CC4=O)C5=CC=C(C=C5)O)O)OC)O)O)O)O)O	AKR1B1	0.105306196
Perilloside C	146763-94-2	CC(=C)C1CCC(CC1)COC2C(C(C(C(O2)CO)O)O)O	PSEN2	0.119403562
Perilloside C	146763-94-2	CC(=C)C1CCC(CC1)COC2C(C(C(C(O2)CO)O)O)O	VEGFA	0.111501865
Perilloside C	146763-94-2	CC(=C)C1CCC(CC1)COC2C(C(C(C(O2)CO)O)O)O	FGF1	0.111501865
Perilloside C	146763-94-2	CC(=C)C1CCC(CC1)COC2C(C(C(C(O2)CO)O)O)O	FGF2	0.111501865
Perilloside C	146763-94-2	CC(=C)C1CCC(CC1)COC2C(C(C(C(O2)CO)O)O)O	HSP90AA1	0.111501865
Perilloside C	146763-94-2	CC(=C)C1CCC(CC1)COC2C(C(C(C(O2)CO)O)O)O	HPSE	0.111501865
Perilloside C	146763-94-2	CC(=C)C1CCC(CC1)COC2C(C(C(C(O2)CO)O)O)O	IL2	0.111501865
Perilloside C	146763-94-2	CC(=C)C1CCC(CC1)COC2C(C(C(C(O2)CO)O)O)O	ADORA1	0.111501865
Perilloside C	146763-94-2	CC(=C)C1CCC(CC1)COC2C(C(C(C(O2)CO)O)O)O	SLC5A1	0.111501865
Perilloside C	146763-94-2	CC(=C)C1CCC(CC1)COC2C(C(C(C(O2)CO)O)O)O	ADK	0.111501865
Perilloside C	146763-94-2	CC(=C)C1CCC(CC1)COC2C(C(C(C(O2)CO)O)O)O	SLC29A1	0.111501865
Perilloside C	146763-94-2	CC(=C)C1CCC(CC1)COC2C(C(C(C(O2)CO)O)O)O	ADORA2A	0.111501865
Perilloside C	146763-94-2	CC(=C)C1CCC(CC1)COC2C(C(C(C(O2)CO)O)O)O	ADORA3	0.111501865
Perilloside C	146763-94-2	CC(=C)C1CCC(CC1)COC2C(C(C(C(O2)CO)O)O)O	PYGL	0.111501865
Perilloside C	146763-94-2	CC(=C)C1CCC(CC1)COC2C(C(C(C(O2)CO)O)O)O	STAT3	0.111501865
Perilloside C	146763-94-2	CC(=C)C1CCC(CC1)COC2C(C(C(C(O2)CO)O)O)O	RORC	0.111501865
Perilloside C	146763-94-2	CC(=C)C1CCC(CC1)COC2C(C(C(C(O2)CO)O)O)O	LGALS3	0.111501865
Perilloside C	146763-94-2	CC(=C)C1CCC(CC1)COC2C(C(C(C(O2)CO)O)O)O	HK2	0.111501865
Perilloside C	146763-94-2	CC(=C)C1CCC(CC1)COC2C(C(C(C(O2)CO)O)O)O	HK1	0.111501865
Perilloside C	146763-94-2	CC(=C)C1CCC(CC1)COC2C(C(C(C(O2)CO)O)O)O	ADORA2B	0.111501865
Perilloside C	146763-	CC(=C)C1CCC(CC1)COC2C(C(C(C(O2)CO)O)O)O	MAPK14	0.111501865

	94-2			
Perilloside C	146763-94-2	CC(=C)C1CCC(CC1)COC2C(C(C(C(O2)CO)O)O)O	LGALS4	0.111501865
Perilloside C	146763-94-2	CC(=C)C1CCC(CC1)COC2C(C(C(C(O2)CO)O)O)O	LGALS8	0.111501865
Perilloside C	146763-94-2	CC(=C)C1CCC(CC1)COC2C(C(C(C(O2)CO)O)O)O	PTAFR	0.111501865
Perilloside C	146763-94-2	CC(=C)C1CCC(CC1)COC2C(C(C(C(O2)CO)O)O)O	CTSK	0.111501865
Perilloside C	146763-94-2	CC(=C)C1CCC(CC1)COC2C(C(C(C(O2)CO)O)O)O	CTSS	0.111501865
Perilloside C	146763-94-2	CC(=C)C1CCC(CC1)COC2C(C(C(C(O2)CO)O)O)O	P4HTM	0.111501865
Perilloside C	146763-94-2	CC(=C)C1CCC(CC1)COC2C(C(C(C(O2)CO)O)O)O	CTSL	0.111501865
Perilloside C	146763-94-2	CC(=C)C1CCC(CC1)COC2C(C(C(C(O2)CO)O)O)O	PFKFB3	0.111501865
Perilloside C	146763-94-2	CC(=C)C1CCC(CC1)COC2C(C(C(C(O2)CO)O)O)O	BCL2L1	0.111501865
Perilloside C	146763-94-2	CC(=C)C1CCC(CC1)COC2C(C(C(C(O2)CO)O)O)O	ADRA2A	0.111501865
Perilloside C	146763-94-2	CC(=C)C1CCC(CC1)COC2C(C(C(C(O2)CO)O)O)O	ADRA2C	0.111501865
Perilloside C	146763-94-2	CC(=C)C1CCC(CC1)COC2C(C(C(C(O2)CO)O)O)O	ADRA2B	0.111501865
Perilloside C	146763-94-2	CC(=C)C1CCC(CC1)COC2C(C(C(C(O2)CO)O)O)O	DRD1	0.111501865
Perilloside C	146763-94-2	CC(=C)C1CCC(CC1)COC2C(C(C(C(O2)CO)O)O)O	ADRA1D	0.111501865
Perilloside C	146763-94-2	CC(=C)C1CCC(CC1)COC2C(C(C(C(O2)CO)O)O)O	CYP2D6	0.111501865
Perilloside C	146763-94-2	CC(=C)C1CCC(CC1)COC2C(C(C(C(O2)CO)O)O)O	ADRA1A	0.111501865
Perilloside C	146763-94-2	CC(=C)C1CCC(CC1)COC2C(C(C(C(O2)CO)O)O)O	HTR1B	0.111501865
Perilloside C	146763-94-2	CC(=C)C1CCC(CC1)COC2C(C(C(C(O2)CO)O)O)O	MAP2K1	0.111501865
Perilloside C	146763-94-2	CC(=C)C1CCC(CC1)COC2C(C(C(C(O2)CO)O)O)O	GPR35	0.111501865
Perilloside C	146763-94-2	CC(=C)C1CCC(CC1)COC2C(C(C(C(O2)CO)O)O)O	HSPA5	0.111501865
Perilloside C	146763-94-2	CC(=C)C1CCC(CC1)COC2C(C(C(C(O2)CO)O)O)O	SLC5A4	0.111501865
Perilloside C	146763-	CC(=C)C1CCC(CC1)COC2C(C(C(C(O2)CO)O)O)O	HSPA8	0.111501865

	94-2			
Perilloside C	146763-94-2	CC(=C)C1CCC(CC1)COC2C(C(C(C(O2)CO)O)O)O	SLC28A2	0.111501865
Perilloside C	146763-94-2	CC(=C)C1CCC(CC1)COC2C(C(C(C(O2)CO)O)O)O	AKR1B1	0.111501865
Perilloside C	146763-94-2	CC(=C)C1CCC(CC1)COC2C(C(C(C(O2)CO)O)O)O	EGFR	0.111501865
Perilloside C	146763-94-2	CC(=C)C1CCC(CC1)COC2C(C(C(C(O2)CO)O)O)O	PSENEN	0.119403562
Perilloside C	146763-94-2	CC(=C)C1CCC(CC1)COC2C(C(C(C(O2)CO)O)O)O	NCSTN	0.119403562
Perilloside C	146763-94-2	CC(=C)C1CCC(CC1)COC2C(C(C(C(O2)CO)O)O)O	APH1A	0.119403562
Perilloside C	146763-94-2	CC(=C)C1CCC(CC1)COC2C(C(C(C(O2)CO)O)O)O	PSEN1	0.119403562
Perilloside C	146763-94-2	CC(=C)C1CCC(CC1)COC2C(C(C(C(O2)CO)O)O)O	APH1B	0.119403562
Sampangine	116664-93-8	C1=CC=C2C(=C1)C3=NC=CC4=C3C(=NC=C4)C2=O	METAP1	0.071715932
Sampangine	116664-93-8	C1=CC=C2C(=C1)C3=NC=CC4=C3C(=NC=C4)C2=O	PAOX	0.053556076
Sampangine	116664-93-8	C1=CC=C2C(=C1)C3=NC=CC4=C3C(=NC=C4)C2=O	EPHX1	0.053556076
Sampangine	116664-93-8	C1=CC=C2C(=C1)C3=NC=CC4=C3C(=NC=C4)C2=O	CHRNA4	0.053556076
Sampangine	116664-93-8	C1=CC=C2C(=C1)C3=NC=CC4=C3C(=NC=C4)C2=O	CHRNA3	0.053556076
Sampangine	116664-93-8	C1=CC=C2C(=C1)C3=NC=CC4=C3C(=NC=C4)C2=O	RNPEP	0.053556076
Sampangine	116664-93-8	C1=CC=C2C(=C1)C3=NC=CC4=C3C(=NC=C4)C2=O	CHRNA7	0.053556076
Sampangine	116664-93-8	C1=CC=C2C(=C1)C3=NC=CC4=C3C(=NC=C4)C2=O	DNPEP	0.053556076
Sampangine	116664-93-8	C1=CC=C2C(=C1)C3=NC=CC4=C3C(=NC=C4)C2=O	SLC5A7	0.053556076
Sampangine	116664-93-8	C1=CC=C2C(=C1)C3=NC=CC4=C3C(=NC=C4)C2=O	DPP4	0.053556076
Sampangine	116664-93-8	C1=CC=C2C(=C1)C3=NC=CC4=C3C(=NC=C4)C2=O	DNM1	0.053556076
Sampangine	116664-93-8	C1=CC=C2C(=C1)C3=NC=CC4=C3C(=NC=C4)C2=O	BCHE	0.053556076
Sampangine	116664-93-8	C1=CC=C2C(=C1)C3=NC=CC4=C3C(=NC=C4)C2=O	CHRNB4	0.053556076
Sampangine	116664-	C1=CC=C2C(=C1)C3=NC=CC4=C3C(=NC=C4)C2=O	CHRNB2	0.053556076

	93-8			
Solanolone	105933-57-1	CC1=CC2=C(C=C1O)C(=O)C(C3C2C(C(CC3)O)O)O	—	—
Sterol	—	C1CC2CCC3C4CCC(CC4CCC3C2C1)O	NR1I3	0.75133882
Sterol	—	C1CC2CCC3C4CCC(CC4CCC3C2C1)O	AR	0.743257372
Sterol	—	C1CC2CCC3C4CCC(CC4CCC3C2C1)O	ESR1	0.607142651
Sterol	—	C1CC2CCC3C4CCC(CC4CCC3C2C1)O	CDC25A	0.552546459
Sterol	—	C1CC2CCC3C4CCC(CC4CCC3C2C1)O	CDC25B	0.552546459
Sterol	—	C1CC2CCC3C4CCC(CC4CCC3C2C1)O	UGT2B7	0.534621858
Sterol	—	C1CC2CCC3C4CCC(CC4CCC3C2C1)O	ESR2	0.489298491
Sterol	—	C1CC2CCC3C4CCC(CC4CCC3C2C1)O	SHBG	0.452899738
Sterol	—	C1CC2CCC3C4CCC(CC4CCC3C2C1)O	HSD11B1	0.207865842
Sterol	—	C1CC2CCC3C4CCC(CC4CCC3C2C1)O	NR1H4	0.171518552
Sterol	—	C1CC2CCC3C4CCC(CC4CCC3C2C1)O	GPBAR1	0.171518552
Sterol	—	C1CC2CCC3C4CCC(CC4CCC3C2C1)O	SHH	0.171518552
Sterol	—	C1CC2CCC3C4CCC(CC4CCC3C2C1)O	TRPM8	0.162404208
Sterol	—	C1CC2CCC3C4CCC(CC4CCC3C2C1)O	CA1	0.162404208
Sterol	—	C1CC2CCC3C4CCC(CC4CCC3C2C1)O	CA2	0.080792387
Sterol	—	C1CC2CCC3C4CCC(CC4CCC3C2C1)O	CA4	0.071715932
Sterol	—	C1CC2CCC3C4CCC(CC4CCC3C2C1)O	POLA1	0.071715932
Sterol	—	C1CC2CCC3C4CCC(CC4CCC3C2C1)O	NPC1L1	0.071715932
Sterol	—	C1CC2CCC3C4CCC(CC4CCC3C2C1)O	CYP19A1	0.062621967
Sterol	—	C1CC2CCC3C4CCC(CC4CCC3C2C1)O	NR1H3	0.062621967
Sterol	—	C1CC2CCC3C4CCC(CC4CCC3C2C1)O	SERPINA6	0.053556076
Sterol	—	C1CC2CCC3C4CCC(CC4CCC3C2C1)O	G6PD	0.053556076
Sterol	—	C1CC2CCC3C4CCC(CC4CCC3C2C1)O	CNR1	0.053556076
Sterol	—	C1CC2CCC3C4CCC(CC4CCC3C2C1)O	CNR2	0.053556076
Sterol	—	C1CC2CCC3C4CCC(CC4CCC3C2C1)O	GABBR1	0.053556076
Sterol	—	C1CC2CCC3C4CCC(CC4CCC3C2C1)O	HSD17B3	0.053556076
Sterol	—	C1CC2CCC3C4CCC(CC4CCC3C2C1)O	SLC6A3	0.053556076
Sterol	—	C1CC2CCC3C4CCC(CC4CCC3C2C1)O	VDR	0.053556076
Sterol	—	C1CC2CCC3C4CCC(CC4CCC3C2C1)O	GABRA2	0.053556076
Sterol	—	C1CC2CCC3C4CCC(CC4CCC3C2C1)O	AKR1B10	0.053556076
Sterol	—	C1CC2CCC3C4CCC(CC4CCC3C2C1)O	CYP51A1	0.053556076
Sterol	—	C1CC2CCC3C4CCC(CC4CCC3C2C1)O	NR3C1	0.053556076
Sterol	—	C1CC2CCC3C4CCC(CC4CCC3C2C1)O	GABRB2	0.053556076
Sterol	—	C1CC2CCC3C4CCC(CC4CCC3C2C1)O	GABRG2	0.053556076
2-Dodecylbenzenesulfonic acid	27176-87-0	CCCCCCCCCCCCC1=CC=CC=C1S(=O)(=O)O	CHRM2	0.111501865
2-Dodecylbenzenesulfonic acid	27176-87-0	CCCCCCCCCCCCC1=CC=CC=C1S(=O)(=O)O	HTR1A	0.111501865
2-Dodecylbenzenesulfonic acid	27176-87-0	CCCCCCCCCCCCC1=CC=CC=C1S(=O)(=O)O	GRM5	0.111501865
2-Dodecylbenzenesulfonic	27176-8	CCCCCCCCCCCCC1=CC=CC=C1S(=O)(=O)O	PTGER4	0.111501865

acid	7-0			
2-Dodecylbenzenesulfonic acid	27176-87-0	CCCCCCCCCCCCC1=CC=CC=C1S(=O)(=O)O	PTGER2	0.111501865
2-Dodecylbenzenesulfonic acid	27176-87-0	CCCCCCCCCCCCC1=CC=CC=C1S(=O)(=O)O	ALOX5	0.111501865
2-Dodecylbenzenesulfonic acid	27176-87-0	CCCCCCCCCCCCC1=CC=CC=C1S(=O)(=O)O	LSS	0.111501865
2-Dodecylbenzenesulfonic acid	27176-87-0	CCCCCCCCCCCCC1=CC=CC=C1S(=O)(=O)O	MAOB	0.111501865
2-Dodecylbenzenesulfonic acid	27176-87-0	CCCCCCCCCCCCC1=CC=CC=C1S(=O)(=O)O	PDE10A	0.111501865
2-Dodecylbenzenesulfonic acid	27176-87-0	CCCCCCCCCCCCC1=CC=CC=C1S(=O)(=O)O	CYP11B1	0.111501865
2-Dodecylbenzenesulfonic acid	27176-87-0	CCCCCCCCCCCCC1=CC=CC=C1S(=O)(=O)O	CYP19A1	0.111501865
2-Dodecylbenzenesulfonic acid	27176-87-0	CCCCCCCCCCCCC1=CC=CC=C1S(=O)(=O)O	PDE4A	0.111501865
2-Dodecylbenzenesulfonic acid	27176-87-0	CCCCCCCCCCCCC1=CC=CC=C1S(=O)(=O)O	CYP11B2	0.111501865
2-Dodecylbenzenesulfonic acid	27176-87-0	CCCCCCCCCCCCC1=CC=CC=C1S(=O)(=O)O	PDE4B	0.111501865
2-Dodecylbenzenesulfonic acid	27176-87-0	CCCCCCCCCCCCC1=CC=CC=C1S(=O)(=O)O	KCNA5	0.111501865
2-Dodecylbenzenesulfonic acid	27176-87-0	CCCCCCCCCCCCC1=CC=CC=C1S(=O)(=O)O	DRD2	0.111501865
2-Dodecylbenzenesulfonic acid	27176-87-0	CCCCCCCCCCCCC1=CC=CC=C1S(=O)(=O)O	PDE4D	0.111501865
2-Dodecylbenzenesulfonic acid	27176-87-0	CCCCCCCCCCCCC1=CC=CC=C1S(=O)(=O)O	CHRNA7	0.111501865
2-Dodecylbenzenesulfonic acid	27176-87-0	CCCCCCCCCCCCC1=CC=CC=C1S(=O)(=O)O	P2RX7	0.111501865
2-Dodecylbenzenesulfonic acid	27176-87-0	CCCCCCCCCCCCC1=CC=CC=C1S(=O)(=O)O	GRM2	0.111501865
2-Dodecylbenzenesulfonic acid	27176-87-0	CCCCCCCCCCCCC1=CC=CC=C1S(=O)(=O)O	CHRM4	0.111501865
2-Dodecylbenzenesulfonic acid	27176-87-0	CCCCCCCCCCCCC1=CC=CC=C1S(=O)(=O)O	AGTR1	0.111501865
2-Dodecylbenzenesulfonic acid	27176-87-0	CCCCCCCCCCCCC1=CC=CC=C1S(=O)(=O)O	PPARG	0.111501865
2-Dodecylbenzenesulfonic acid	27176-87-0	CCCCCCCCCCCCC1=CC=CC=C1S(=O)(=O)O	CNR2	0.111501865
2-Dodecylbenzenesulfonic acid	27176-87-0	CCCCCCCCCCCCC1=CC=CC=C1S(=O)(=O)O	TGM2	0.111501865
2-Dodecylbenzenesulfonic	27176-8	CCCCCCCCCCCCC1=CC=CC=C1S(=O)(=O)O	MET	0.111501865

acid	7-0			
2-Dodecylbenzenesulfonic acid	27176-87-0	CCCCCCCCCCCCC1=CC=CC=C1S(=O)(=O)O	EPHX2	0.111501865
2-Dodecylbenzenesulfonic acid	27176-87-0	CCCCCCCCCCCCC1=CC=CC=C1S(=O)(=O)O	HCRTR2	0.111501865
2-Dodecylbenzenesulfonic acid	27176-87-0	CCCCCCCCCCCCC1=CC=CC=C1S(=O)(=O)O	HCRTR1	0.111501865
2-Dodecylbenzenesulfonic acid	27176-87-0	CCCCCCCCCCCCC1=CC=CC=C1S(=O)(=O)O	CYP24A1	0.111501865
2-Dodecylbenzenesulfonic acid	27176-87-0	CCCCCCCCCCCCC1=CC=CC=C1S(=O)(=O)O	CYP27A1	0.111501865
2-Dodecylbenzenesulfonic acid	27176-87-0	CCCCCCCCCCCCC1=CC=CC=C1S(=O)(=O)O	SLC5A7	0.111501865
2-Dodecylbenzenesulfonic acid	27176-87-0	CCCCCCCCCCCCC1=CC=CC=C1S(=O)(=O)O	PABPC1	0.111501865
2-Dodecylbenzenesulfonic acid	27176-87-0	CCCCCCCCCCCCC1=CC=CC=C1S(=O)(=O)O	IMPDH2	0.111501865
2-Dodecylbenzenesulfonic acid	27176-87-0	CCCCCCCCCCCCC1=CC=CC=C1S(=O)(=O)O	CHRM1	0.111501865
2-Dodecylbenzenesulfonic acid	27176-87-0	CCCCCCCCCCCCC1=CC=CC=C1S(=O)(=O)O	OPRD1	0.111501865
2-Dodecylbenzenesulfonic acid	27176-87-0	CCCCCCCCCCCCC1=CC=CC=C1S(=O)(=O)O	CPT1A	0.111501865
2-Dodecylbenzenesulfonic acid	27176-87-0	CCCCCCCCCCCCC1=CC=CC=C1S(=O)(=O)O	KCNJ1	0.111501865
2-Dodecylbenzenesulfonic acid	27176-87-0	CCCCCCCCCCCCC1=CC=CC=C1S(=O)(=O)O	KCNH2	0.111501865
2-Dodecylbenzenesulfonic acid	27176-87-0	CCCCCCCCCCCCC1=CC=CC=C1S(=O)(=O)O	ENPP2	0.111501865
2-Dodecylbenzenesulfonic acid	27176-87-0	CCCCCCCCCCCCC1=CC=CC=C1S(=O)(=O)O	SMO	0.111501865
2-Dodecylbenzenesulfonic acid	27176-87-0	CCCCCCCCCCCCC1=CC=CC=C1S(=O)(=O)O	ADORA1	0.111501865
2-Dodecylbenzenesulfonic acid	27176-87-0	CCCCCCCCCCCCC1=CC=CC=C1S(=O)(=O)O	ADORA2A	0.111501865
2-Dodecylbenzenesulfonic acid	27176-87-0	CCCCCCCCCCCCC1=CC=CC=C1S(=O)(=O)O	HSP90AA1	0.111501865
2-Dodecylbenzenesulfonic acid	27176-87-0	CCCCCCCCCCCCC1=CC=CC=C1S(=O)(=O)O	CMA1	0.111501865
2-Dodecylbenzenesulfonic acid	27176-87-0	CCCCCCCCCCCCC1=CC=CC=C1S(=O)(=O)O	MCL1	0.111501865
2-Dodecylbenzenesulfonic acid	27176-87-0	CCCCCCCCCCCCC1=CC=CC=C1S(=O)(=O)O	SLC6A9	0.111501865
2-Dodecylbenzenesulfonic	27176-8	CCCCCCCCCCCCC1=CC=CC=C1S(=O)(=O)O	TACR1	0.111501865

acid	7-0			
2-Dodecylbenzenesulfonic acid	27176-87-0	CCCCCCCCCCCCC1=CC=CC=C1S(=O)(=O)O	TSPO	0.111501865
2-Dodecylbenzenesulfonic acid	27176-87-0	CCCCCCCCCCCCC1=CC=CC=C1S(=O)(=O)O	CCNC	0.111501865
2-Dodecylbenzenesulfonic acid	27176-87-0	CCCCCCCCCCCCC1=CC=CC=C1S(=O)(=O)O	CDK8	0.111501865
2-Dodecylbenzenesulfonic acid	27176-87-0	CCCCCCCCCCCCC1=CC=CC=C1S(=O)(=O)O	RAF1	0.111501865
2-Dodecylbenzenesulfonic acid	27176-87-0	CCCCCCCCCCCCC1=CC=CC=C1S(=O)(=O)O	CNR1	0.111501865
2-Dodecylbenzenesulfonic acid	27176-87-0	CCCCCCCCCCCCC1=CC=CC=C1S(=O)(=O)O	HTR2A	0.111501865
2-Dodecylbenzenesulfonic acid	27176-87-0	CCCCCCCCCCCCC1=CC=CC=C1S(=O)(=O)O	ADAMTS5	0.111501865
2-Dodecylbenzenesulfonic acid	27176-87-0	CCCCCCCCCCCCC1=CC=CC=C1S(=O)(=O)O	CXCR2	0.111501865
2-Dodecylbenzenesulfonic acid	27176-87-0	CCCCCCCCCCCCC1=CC=CC=C1S(=O)(=O)O	ELANE	0.111501865
2-Dodecylbenzenesulfonic acid	27176-87-0	CCCCCCCCCCCCC1=CC=CC=C1S(=O)(=O)O	MAPK14	0.111501865
2-Dodecylbenzenesulfonic acid	27176-87-0	CCCCCCCCCCCCC1=CC=CC=C1S(=O)(=O)O	MAPK11	0.111501865
2-Dodecylbenzenesulfonic acid	27176-87-0	CCCCCCCCCCCCC1=CC=CC=C1S(=O)(=O)O	BRAF	0.111501865
2-Dodecylbenzenesulfonic acid	27176-87-0	CCCCCCCCCCCCC1=CC=CC=C1S(=O)(=O)O	HTR1D	0.111501865
2-Dodecylbenzenesulfonic acid	27176-87-0	CCCCCCCCCCCCC1=CC=CC=C1S(=O)(=O)O	SLC6A4	0.111501865
2-Dodecylbenzenesulfonic acid	27176-87-0	CCCCCCCCCCCCC1=CC=CC=C1S(=O)(=O)O	CTSK	0.111501865
2-Dodecylbenzenesulfonic acid	27176-87-0	CCCCCCCCCCCCC1=CC=CC=C1S(=O)(=O)O	KDR	0.111501865
2-Dodecylbenzenesulfonic acid	27176-87-0	CCCCCCCCCCCCC1=CC=CC=C1S(=O)(=O)O	FABP4	0.111501865
2-Dodecylbenzenesulfonic acid	27176-87-0	CCCCCCCCCCCCC1=CC=CC=C1S(=O)(=O)O	FABP3	0.111501865
2-Dodecylbenzenesulfonic acid	27176-87-0	CCCCCCCCCCCCC1=CC=CC=C1S(=O)(=O)O	CRHR1	0.111501865
2-Dodecylbenzenesulfonic acid	27176-87-0	CCCCCCCCCCCCC1=CC=CC=C1S(=O)(=O)O	AR	0.111501865
2-Dodecylbenzenesulfonic acid	27176-87-0	CCCCCCCCCCCCC1=CC=CC=C1S(=O)(=O)O	PGR	0.111501865
2-Dodecylbenzenesulfonic	27176-8	CCCCCCCCCCCCC1=CC=CC=C1S(=O)(=O)O	PIM1	0.111501865

acid	7-0			
2-Dodecylbenzenesulfonic acid	27176-87-0	CCCCCCCCCCCCC1=CC=CC=C1S(=O)(=O)O	HTR6	0.111501865
2-Dodecylbenzenesulfonic acid	27176-87-0	CCCCCCCCCCCCC1=CC=CC=C1S(=O)(=O)O	NAMPT	0.111501865
2-Dodecylbenzenesulfonic acid	27176-87-0	CCCCCCCCCCCCC1=CC=CC=C1S(=O)(=O)O	SSTR3	0.111501865
2-Dodecylbenzenesulfonic acid	27176-87-0	CCCCCCCCCCCCC1=CC=CC=C1S(=O)(=O)O	DYRK1A	0.111501865
2-Dodecylbenzenesulfonic acid	27176-87-0	CCCCCCCCCCCCC1=CC=CC=C1S(=O)(=O)O	ADRA1A	0.111501865
2-Dodecylbenzenesulfonic acid	27176-87-0	CCCCCCCCCCCCC1=CC=CC=C1S(=O)(=O)O	PFKFB3	0.111501865
2-Dodecylbenzenesulfonic acid	27176-87-0	CCCCCCCCCCCCC1=CC=CC=C1S(=O)(=O)O	DUSP3	0.111501865
2-Dodecylbenzenesulfonic acid	27176-87-0	CCCCCCCCCCCCC1=CC=CC=C1S(=O)(=O)O	PARP1	0.111501865
2-Dodecylbenzenesulfonic acid	27176-87-0	CCCCCCCCCCCCC1=CC=CC=C1S(=O)(=O)O	CPT2	0.111501865
2-Dodecylbenzenesulfonic acid	27176-87-0	CCCCCCCCCCCCC1=CC=CC=C1S(=O)(=O)O	PIK3CA	0.111501865
2-Dodecylbenzenesulfonic acid	27176-87-0	CCCCCCCCCCCCC1=CC=CC=C1S(=O)(=O)O	HDAC6	0.111501865
2-Dodecylbenzenesulfonic acid	27176-87-0	CCCCCCCCCCCCC1=CC=CC=C1S(=O)(=O)O	MTNR1A	0.111501865
2-Dodecylbenzenesulfonic acid	27176-87-0	CCCCCCCCCCCCC1=CC=CC=C1S(=O)(=O)O	INSR	0.111501865
2-Dodecylbenzenesulfonic acid	27176-87-0	CCCCCCCCCCCCC1=CC=CC=C1S(=O)(=O)O	ADRA1D	0.111501865
2-Dodecylbenzenesulfonic acid	27176-87-0	CCCCCCCCCCCCC1=CC=CC=C1S(=O)(=O)O	FAAH	0.111501865
2-Dodecylbenzenesulfonic acid	27176-87-0	CCCCCCCCCCCCC1=CC=CC=C1S(=O)(=O)O	ADRA1B	0.111501865
2-Dodecylbenzenesulfonic acid	27176-87-0	CCCCCCCCCCCCC1=CC=CC=C1S(=O)(=O)O	TRPV1	0.111501865
2-Dodecylbenzenesulfonic acid	27176-87-0	CCCCCCCCCCCCC1=CC=CC=C1S(=O)(=O)O	ABCG2	0.111501865
2-Dodecylbenzenesulfonic acid	27176-87-0	CCCCCCCCCCCCC1=CC=CC=C1S(=O)(=O)O	LRRK2	0.111501865
2-Dodecylbenzenesulfonic acid	27176-87-0	CCCCCCCCCCCCC1=CC=CC=C1S(=O)(=O)O	NDUFA4	0.111501865
2-Dodecylbenzenesulfonic acid	27176-87-0	CCCCCCCCCCCCC1=CC=CC=C1S(=O)(=O)O	HTR7	0.111501865
2-Dodecylbenzenesulfonic	27176-8	CCCCCCCCCCCCC1=CC=CC=C1S(=O)(=O)O	MMP9	0.111501865

acid	7-0			
2-Dodecylbenzenesulfonic acid	27176-87-0	CCCCCCCCCCCCC1=CC=CC=C1S(=O)(=O)O	HDAC1	0.111501865
2-Dodecylbenzenesulfonic acid	27176-87-0	CCCCCCCCCCCCC1=CC=CC=C1S(=O)(=O)O	CTSV	0.111501865
2-Dodecylbenzenesulfonic acid	27176-87-0	CCCCCCCCCCCCC1=CC=CC=C1S(=O)(=O)O	CTSL	0.111501865
2-Dodecylbenzenesulfonic acid	27176-87-0	CCCCCCCCCCCCC1=CC=CC=C1S(=O)(=O)O	PIM2	0.111501865
3,4',5-Trihydroxy-3',7-dimethoxyflavanone	—	COC1=CC(=C2C(=C1)OC(C(C2=O)O)C3=CC(=C(C=C3)O)OC)O	—	—
3-O-Caffeoyl-1-O-methyl quinic acid	—	COC1(CC(C(C(C1)OC(=O)C=CC2=CC(=C(C=C2)O)O)O)O)C(=O)O	AKR1B1	0.661195363
3-O-Caffeoyl-1-O-methyl quinic acid	—	COC1(CC(C(C(C1)OC(=O)C=CC2=CC(=C(C=C2)O)O)O)O)C(=O)O	AKR1B10	0.628755002
3-O-Caffeoyl-1-O-methyl quinic acid	—	COC1(CC(C(C(C1)OC(=O)C=CC2=CC(=C(C=C2)O)O)O)O)C(=O)O	MMP13	0.458331873
3-O-Caffeoyl-1-O-methyl quinic acid	—	COC1(CC(C(C(C1)OC(=O)C=CC2=CC(=C(C=C2)O)O)O)O)C(=O)O	MMP2	0.458331873
3-O-Caffeoyl-1-O-methyl quinic acid	—	COC1(CC(C(C(C1)OC(=O)C=CC2=CC(=C(C=C2)O)O)O)O)C(=O)O	MMP12	0.174258099
3-O-Caffeoyl-1-O-methyl quinic acid	—	COC1(CC(C(C(C1)OC(=O)C=CC2=CC(=C(C=C2)O)O)O)O)C(=O)O	APP	0.141778404
3-O-Caffeoyl-1-O-methyl quinic acid	—	COC1(CC(C(C(C1)OC(=O)C=CC2=CC(=C(C=C2)O)O)O)O)C(=O)O	SLC37A4	0.109339753
3-O-Caffeoyl-1-O-methyl quinic acid	—	COC1(CC(C(C(C1)OC(=O)C=CC2=CC(=C(C=C2)O)O)O)O)C(=O)O	PYGL	0.109339753
3-O-Caffeoyl-1-O-methyl quinic acid	—	COC1(CC(C(C(C1)OC(=O)C=CC2=CC(=C(C=C2)O)O)O)O)C(=O)O	CA2	0.109339753
3-O-Caffeoyl-1-O-methyl quinic acid	—	COC1(CC(C(C(C1)OC(=O)C=CC2=CC(=C(C=C2)O)O)O)O)C(=O)O	CA1	0.109339753
3-O-Caffeoyl-1-O-methyl quinic acid	—	COC1(CC(C(C(C1)OC(=O)C=CC2=CC(=C(C=C2)O)O)O)O)C(=O)O	CASP3	0.109339753
3-O-Caffeoyl-1-O-methyl quinic acid	—	COC1(CC(C(C(C1)OC(=O)C=CC2=CC(=C(C=C2)O)O)O)O)C(=O)O	CA12	0.109339753
3-O-Caffeoyl-1-O-methyl quinic acid	—	COC1(CC(C(C(C1)OC(=O)C=CC2=CC(=C(C=C2)O)O)O)O)C(=O)O	CA9	0.109339753
3-O-Caffeoyl-1-O-methyl quinic acid	—	COC1(CC(C(C(C1)OC(=O)C=CC2=CC(=C(C=C2)O)O)O)O)C(=O)O	NEU4	0.109339753
3-O-Caffeoyl-1-O-methyl quinic acid	—	COC1(CC(C(C(C1)OC(=O)C=CC2=CC(=C(C=C2)O)O)O)O)C(=O)O	ECE1	0.109339753
3-O-Caffeoyl-1-O-methyl quinic acid	—	COC1(CC(C(C(C1)OC(=O)C=CC2=CC(=C(C=C2)O)O)O)O)C(=O)O	CASP6	0.109339753
3-O-Caffeoyl-1-O-methyl	—	COC1(CC(C(C(C1)OC(=O)C=CC2=CC(=C(C=C2)O)O)O	CASP7	0.109339753

quinic acid		)O)C(=O)O		
3-O-Caffeoyl-1-O-methyl quinic acid	—	COC1(CC(C(C(C1)OC(=O)C=CC2=CC(=C(C=C2)O)O)O)O)C(=O)O	CASP8	0.109339753
3-O-Caffeoyl-1-O-methyl quinic acid	—	COC1(CC(C(C(C1)OC(=O)C=CC2=CC(=C(C=C2)O)O)O)O)C(=O)O	CASP1	0.109339753
3-O-Caffeoyl-1-O-methyl quinic acid	—	COC1(CC(C(C(C1)OC(=O)C=CC2=CC(=C(C=C2)O)O)O)O)C(=O)O	CASP2	0.109339753
3-O-Caffeoyl-1-O-methyl quinic acid	—	COC1(CC(C(C(C1)OC(=O)C=CC2=CC(=C(C=C2)O)O)O)O)C(=O)O	PRKCD	0.109339753
3-O-Caffeoyl-1-O-methyl quinic acid	—	COC1(CC(C(C(C1)OC(=O)C=CC2=CC(=C(C=C2)O)O)O)O)C(=O)O	PRKCA	0.109339753
3-O-Caffeoyl-1-O-methyl quinic acid	—	COC1(CC(C(C(C1)OC(=O)C=CC2=CC(=C(C=C2)O)O)O)O)C(=O)O	ABCB1	0.109339753
3-O-Caffeoyl-1-O-methyl quinic acid	—	COC1(CC(C(C(C1)OC(=O)C=CC2=CC(=C(C=C2)O)O)O)O)C(=O)O	ELANE	0.109339753
3-O-Caffeoyl-1-O-methyl quinic acid	—	COC1(CC(C(C(C1)OC(=O)C=CC2=CC(=C(C=C2)O)O)O)O)C(=O)O	CA5B	0.109339753
3-O-Caffeoyl-1-O-methyl quinic acid	—	COC1(CC(C(C(C1)OC(=O)C=CC2=CC(=C(C=C2)O)O)O)O)C(=O)O	MMP16	0.109339753
3-O-Caffeoyl-1-O-methyl quinic acid	—	COC1(CC(C(C(C1)OC(=O)C=CC2=CC(=C(C=C2)O)O)O)O)C(=O)O	MMP14	0.109339753
3-O-Caffeoyl-1-O-methyl quinic acid	—	COC1(CC(C(C(C1)OC(=O)C=CC2=CC(=C(C=C2)O)O)O)O)C(=O)O	MMP8	0.109339753
3-O-Caffeoyl-1-O-methyl quinic acid	—	COC1(CC(C(C(C1)OC(=O)C=CC2=CC(=C(C=C2)O)O)O)O)C(=O)O	PDE4D	0.109339753
3-O-Caffeoyl-1-O-methyl quinic acid	—	COC1(CC(C(C(C1)OC(=O)C=CC2=CC(=C(C=C2)O)O)O)O)C(=O)O	PDE9A	0.109339753
3-O-Caffeoyl-1-O-methyl quinic acid	—	COC1(CC(C(C(C1)OC(=O)C=CC2=CC(=C(C=C2)O)O)O)O)C(=O)O	PDE1B	0.109339753
3-O-Caffeoyl-1-O-methyl quinic acid	—	COC1(CC(C(C(C1)OC(=O)C=CC2=CC(=C(C=C2)O)O)O)O)C(=O)O	ENGASE	0.109339753
3-O-Caffeoyl-1-O-methyl quinic acid	—	COC1(CC(C(C(C1)OC(=O)C=CC2=CC(=C(C=C2)O)O)O)O)C(=O)O	NEU2	0.109339753
3-O-Caffeoyl-1-O-methyl quinic acid	—	COC1(CC(C(C(C1)OC(=O)C=CC2=CC(=C(C=C2)O)O)O)O)C(=O)O	MMP1	0.109339753
3-O-Caffeoyl-1-O-methyl quinic acid	—	COC1(CC(C(C(C1)OC(=O)C=CC2=CC(=C(C=C2)O)O)O)O)C(=O)O	CFTR	0.109339753
3-O-Caffeoyl-1-O-methyl quinic acid	—	COC1(CC(C(C(C1)OC(=O)C=CC2=CC(=C(C=C2)O)O)O)O)C(=O)O	MMP9	0.109339753
3-O-Caffeoyl-1-O-methyl quinic acid	—	COC1(CC(C(C(C1)OC(=O)C=CC2=CC(=C(C=C2)O)O)O)O)C(=O)O	HPRT1	0.109339753
3-O-Caffeoyl-1-O-methyl quinic acid	—	COC1(CC(C(C(C1)OC(=O)C=CC2=CC(=C(C=C2)O)O)O)O)C(=O)O	ACE	0.109339753
3-O-Caffeoyl-1-O-methyl	—	COC1(CC(C(C(C1)OC(=O)C=CC2=CC(=C(C=C2)O)O)O	SELL	0.109339753

quinic acid		)O)C(=O)O		
3-O-Caffeoyl-1-O-methyl quinic acid	—	COC1(CC(C(C(C1)OC(=O)C=CC2=CC(=C(C=C2)O)O)O)O)C(=O)O	SELP	0.109339753
3-O-Caffeoyl-1-O-methyl quinic acid	—	COC1(CC(C(C(C1)OC(=O)C=CC2=CC(=C(C=C2)O)O)O)O)C(=O)O	AMPD3	0.109339753
3-O-Caffeoyl-1-O-methyl quinic acid	—	COC1(CC(C(C(C1)OC(=O)C=CC2=CC(=C(C=C2)O)O)O)O)C(=O)O	HK2	0.109339753
3-O-Caffeoyl-1-O-methyl quinic acid	—	COC1(CC(C(C(C1)OC(=O)C=CC2=CC(=C(C=C2)O)O)O)O)C(=O)O	HK1	0.109339753
3-O-Caffeoyl-1-O-methyl quinic acid	—	COC1(CC(C(C(C1)OC(=O)C=CC2=CC(=C(C=C2)O)O)O)O)C(=O)O	FOLH1	0.109339753
3-O-Caffeoyl-1-O-methyl quinic acid	—	COC1(CC(C(C(C1)OC(=O)C=CC2=CC(=C(C=C2)O)O)O)O)C(=O)O	EGLN1	0.109339753
3-O-Caffeoyl-1-O-methyl quinic acid	—	COC1(CC(C(C(C1)OC(=O)C=CC2=CC(=C(C=C2)O)O)O)O)C(=O)O	OGA	0.109339753
3-O-Caffeoyl-1-O-methyl quinic acid	—	COC1(CC(C(C(C1)OC(=O)C=CC2=CC(=C(C=C2)O)O)O)O)C(=O)O	DNMT3B	0.109339753
3-O-Caffeoyl-1-O-methyl quinic acid	—	COC1(CC(C(C(C1)OC(=O)C=CC2=CC(=C(C=C2)O)O)O)O)C(=O)O	KDR	0.109339753
Carvyl propionate	97-45-0	CCC(=O)OC1CC(CC=C1C)C(=C)C	HMGCR	0.061946622
Carvyl propionate	97-45-0	CCC(=O)OC1CC(CC=C1C)C(=C)C	CYP19A1	0.042894212
Carvyl propionate	97-45-0	CCC(=O)OC1CC(CC=C1C)C(=C)C	ADRA2A	0.042894212
Carvyl propionate	97-45-0	CCC(=O)OC1CC(CC=C1C)C(=C)C	ADRA2C	0.042894212
Carvyl propionate	97-45-0	CCC(=O)OC1CC(CC=C1C)C(=C)C	ADRA2B	0.042894212
Carvyl propionate	97-45-0	CCC(=O)OC1CC(CC=C1C)C(=C)C	IL1B	0.042894212
Carvyl propionate	97-45-0	CCC(=O)OC1CC(CC=C1C)C(=C)C	CTSK	0.042894212
Carvyl propionate	97-45-0	CCC(=O)OC1CC(CC=C1C)C(=C)C	CTSL	0.042894212
Carvyl propionate	97-45-0	CCC(=O)OC1CC(CC=C1C)C(=C)C	CTSB	0.042894212
Carvyl propionate	97-45-0	CCC(=O)OC1CC(CC=C1C)C(=C)C	PDE4D	0.042894212
Carvyl propionate	97-45-0	CCC(=O)OC1CC(CC=C1C)C(=C)C	ADRA1A	0.042894212
Carvyl propionate	97-45-0	CCC(=O)OC1CC(CC=C1C)C(=C)C	MAOA	0.042894212
Carvyl propionate	97-45-0	CCC(=O)OC1CC(CC=C1C)C(=C)C	PPARG	0.042894212
Carvyl propionate	97-45-0	CCC(=O)OC1CC(CC=C1C)C(=C)C	EPHX1	0.042894212
Carvyl propionate	97-45-0	CCC(=O)OC1CC(CC=C1C)C(=C)C	CA2	0.042894212
Carvyl propionate	97-45-0	CCC(=O)OC1CC(CC=C1C)C(=C)C	TTL	0.042894212
Carvyl propionate	97-45-0	CCC(=O)OC1CC(CC=C1C)C(=C)C	CTSH	0.042894212
Carvyl propionate	97-45-0	CCC(=O)OC1CC(CC=C1C)C(=C)C	F2	0.042894212
Carvyl propionate	97-45-0	CCC(=O)OC1CC(CC=C1C)C(=C)C	PRSS1	0.042894212
Carvyl propionate	97-45-0	CCC(=O)OC1CC(CC=C1C)C(=C)C	FNTA	0.042894212
Carvyl propionate	97-45-0	CCC(=O)OC1CC(CC=C1C)C(=C)C	RPS6KA5	0.042894212
Carvyl propionate	97-45-0	CCC(=O)OC1CC(CC=C1C)C(=C)C	BRD4	0.042894212
Carvyl propionate	97-45-0	CCC(=O)OC1CC(CC=C1C)C(=C)C	GABRA1	0.042894212
Carvyl propionate	97-45-0	CCC(=O)OC1CC(CC=C1C)C(=C)C	GABRA3	0.042894212
Carvyl propionate	97-45-0	CCC(=O)OC1CC(CC=C1C)C(=C)C	GABRA2	0.042894212

Carvyl propionate	97-45-0	CCC(=O)OC1CC(CC=C1C)C(=C)C	GABRA5	0.042894212
Carvyl propionate	97-45-0	CCC(=O)OC1CC(CC=C1C)C(=C)C	TRPA1	0.042894212
Carvyl propionate	97-45-0	CCC(=O)OC1CC(CC=C1C)C(=C)C	AR	0.042894212
Carvyl propionate	97-45-0	CCC(=O)OC1CC(CC=C1C)C(=C)C	PGR	0.042894212
Carvyl propionate	97-45-0	CCC(=O)OC1CC(CC=C1C)C(=C)C	CYP11B1	0.042894212
Carvyl propionate	97-45-0	CCC(=O)OC1CC(CC=C1C)C(=C)C	GABRA2	0.042894212
Carvyl propionate	97-45-0	CCC(=O)OC1CC(CC=C1C)C(=C)C	CYP11B2	0.042894212
Carvyl propionate	97-45-0	CCC(=O)OC1CC(CC=C1C)C(=C)C	CHRNA4	0.042894212
Carvyl propionate	97-45-0	CCC(=O)OC1CC(CC=C1C)C(=C)C	HSD11B1	0.042894212
Carvyl propionate	97-45-0	CCC(=O)OC1CC(CC=C1C)C(=C)C	PTPN1	0.042894212
Carvyl propionate	97-45-0	CCC(=O)OC1CC(CC=C1C)C(=C)C	ATP12A	0.042894212
Carvyl propionate	97-45-0	CCC(=O)OC1CC(CC=C1C)C(=C)C	GRM2	0.042894212
Carvyl propionate	97-45-0	CCC(=O)OC1CC(CC=C1C)C(=C)C	PLA2G6	0.042894212
Carvyl propionate	97-45-0	CCC(=O)OC1CC(CC=C1C)C(=C)C	ALDH3A1	0.042894212
Carvyl propionate	97-45-0	CCC(=O)OC1CC(CC=C1C)C(=C)C	CDC25C	0.042894212
Carvyl propionate	97-45-0	CCC(=O)OC1CC(CC=C1C)C(=C)C	CDC25A	0.042894212
Carvyl propionate	97-45-0	CCC(=O)OC1CC(CC=C1C)C(=C)C	CTRB1	0.042894212
Carvyl propionate	97-45-0	CCC(=O)OC1CC(CC=C1C)C(=C)C	GRM5	0.042894212
Carvyl propionate	97-45-0	CCC(=O)OC1CC(CC=C1C)C(=C)C	CCR1	0.042894212
Carvyl propionate	97-45-0	CCC(=O)OC1CC(CC=C1C)C(=C)C	CCR8	0.042894212
Carvyl propionate	97-45-0	CCC(=O)OC1CC(CC=C1C)C(=C)C	GABRB3	0.042894212
Carvyl propionate	97-45-0	CCC(=O)OC1CC(CC=C1C)C(=C)C	GABRA1	0.042894212
Carvyl propionate	97-45-0	CCC(=O)OC1CC(CC=C1C)C(=C)C	GABRA5	0.042894212
Carvyl propionate	97-45-0	CCC(=O)OC1CC(CC=C1C)C(=C)C	GABRA2	0.042894212
Carvyl propionate	97-45-0	CCC(=O)OC1CC(CC=C1C)C(=C)C	ADORA2A	0.042894212
Carvyl propionate	97-45-0	CCC(=O)OC1CC(CC=C1C)C(=C)C	TGFBR1	0.042894212
Carvyl propionate	97-45-0	CCC(=O)OC1CC(CC=C1C)C(=C)C	TAAR1	0.042894212
Carvyl propionate	97-45-0	CCC(=O)OC1CC(CC=C1C)C(=C)C	FNTB	0.042894212
Carvyl propionate	97-45-0	CCC(=O)OC1CC(CC=C1C)C(=C)C	GABRA3	0.042894212
Carvyl propionate	97-45-0	CCC(=O)OC1CC(CC=C1C)C(=C)C	GABRG2	0.042894212
Carvyl propionate	97-45-0	CCC(=O)OC1CC(CC=C1C)C(=C)C	GABRB2	0.042894212
Eremopetasidione	163135-94-2	CC1C(CCC2C1(C=C(C(=O)C2)C(=O)C)C)O	CYP19A1	0.328168976
Eremopetasidione	163135-94-2	CC1C(CCC2C1(C=C(C(=O)C2)C(=O)C)C)O	HSD11B1	0.184102383
Eremopetasidione	163135-94-2	CC1C(CCC2C1(C=C(C(=O)C2)C(=O)C)C)O	AR	0.184102383
Eremopetasidione	163135-94-2	CC1C(CCC2C1(C=C(C(=O)C2)C(=O)C)C)O	NR3C1	0.184102383
Eremopetasidione	163135-94-2	CC1C(CCC2C1(C=C(C(=O)C2)C(=O)C)C)O	SIGMAR1	0.14806166
Eremopetasidione	163135-94-2	CC1C(CCC2C1(C=C(C(=O)C2)C(=O)C)C)O	NOS2	0.130044233

Eremopetasidione	163135-94-2	CC1C(CCC2C1(C=C(C(=O)C2)C(=O)C)C)O	PTGES	0.120996593
Eremopetasidione	163135-94-2	CC1C(CCC2C1(C=C(C(=O)C2)C(=O)C)C)O	NPC1L1	0.112023618
Eremopetasidione	163135-94-2	CC1C(CCC2C1(C=C(C(=O)C2)C(=O)C)C)O	NR3C2	0.112023618
Eremopetasidione	163135-94-2	CC1C(CCC2C1(C=C(C(=O)C2)C(=O)C)C)O	CYP17A1	0.103007428
Eremopetasidione	163135-94-2	CC1C(CCC2C1(C=C(C(=O)C2)C(=O)C)C)O	SLC6A3	0.094005352
Eremopetasidione	163135-94-2	CC1C(CCC2C1(C=C(C(=O)C2)C(=O)C)C)O	PTPN11	0.094005352
Eremopetasidione	163135-94-2	CC1C(CCC2C1(C=C(C(=O)C2)C(=O)C)C)O	AKR1B10	0.094005352
Eremopetasidione	163135-94-2	CC1C(CCC2C1(C=C(C(=O)C2)C(=O)C)C)O	PGR	0.094005352
Eremopetasidione	163135-94-2	CC1C(CCC2C1(C=C(C(=O)C2)C(=O)C)C)O	SERPINA6	0.094005352
Eremopetasidione	163135-94-2	CC1C(CCC2C1(C=C(C(=O)C2)C(=O)C)C)O	SHBG	0.094005352
Eremopetasidione	163135-94-2	CC1C(CCC2C1(C=C(C(=O)C2)C(=O)C)C)O	HSD11B2	0.094005352
Eremopetasidione	163135-94-2	CC1C(CCC2C1(C=C(C(=O)C2)C(=O)C)C)O	PTPN2	0.075973634
Eremopetasidione	163135-94-2	CC1C(CCC2C1(C=C(C(=O)C2)C(=O)C)C)O	BACE1	0.075973634
Eremopetasidione	163135-94-2	CC1C(CCC2C1(C=C(C(=O)C2)C(=O)C)C)O	ADORA3	0.075973634
Eremopetasidione	163135-94-2	CC1C(CCC2C1(C=C(C(=O)C2)C(=O)C)C)O	PRKCH	0.075973634
Eremopetasidione	163135-94-2	CC1C(CCC2C1(C=C(C(=O)C2)C(=O)C)C)O	CDC25A	0.066975016
Eremopetasidione	163135-94-2	CC1C(CCC2C1(C=C(C(=O)C2)C(=O)C)C)O	MAPK3	0.066975016
Eremopetasidione	163135-94-2	CC1C(CCC2C1(C=C(C(=O)C2)C(=O)C)C)O	ESR2	0.066975016
Eremopetasidione	163135-94-2	CC1C(CCC2C1(C=C(C(=O)C2)C(=O)C)C)O	ALOX5	0.066975016
Eremopetasidione	163135-94-2	CC1C(CCC2C1(C=C(C(=O)C2)C(=O)C)C)O	BCHE	0.057956876
Eremopetasidione	163135-94-2	CC1C(CCC2C1(C=C(C(=O)C2)C(=O)C)C)O	RORA	0.057956876
Eremopetasidione	163135-94-2	CC1C(CCC2C1(C=C(C(=O)C2)C(=O)C)C)O	PTGS2	0.057956876

Eremopetasidione	163135-94-2	CC1C(CCC2C1(C=C(C(=O)C2)C(=O)C)C)O	PLA2G1B	0.057956876
Eremopetasidione	163135-94-2	CC1C(CCC2C1(C=C(C(=O)C2)C(=O)C)C)O	ACP1	0.057956876
Eremopetasidione	163135-94-2	CC1C(CCC2C1(C=C(C(=O)C2)C(=O)C)C)O	PTPN6	0.057956876
Eremopetasidione	163135-94-2	CC1C(CCC2C1(C=C(C(=O)C2)C(=O)C)C)O	PTPN1	0.048952898
Eremopetasidione	163135-94-2	CC1C(CCC2C1(C=C(C(=O)C2)C(=O)C)C)O	POLB	0.048952898
Eremopetasidione	163135-94-2	CC1C(CCC2C1(C=C(C(=O)C2)C(=O)C)C)O	NR1I3	0.048952898
Eremopetasidione	163135-94-2	CC1C(CCC2C1(C=C(C(=O)C2)C(=O)C)C)O	CD81	0.048952898
Eremopetasidione	163135-94-2	CC1C(CCC2C1(C=C(C(=O)C2)C(=O)C)C)O	IDO1	0.048952898
Eremopetasidione	163135-94-2	CC1C(CCC2C1(C=C(C(=O)C2)C(=O)C)C)O	PDE4D	0.048952898
Eremopetasidione	163135-94-2	CC1C(CCC2C1(C=C(C(=O)C2)C(=O)C)C)O	RORC	0.048952898
Eremopetasidione	163135-94-2	CC1C(CCC2C1(C=C(C(=O)C2)C(=O)C)C)O	FNTA	0.048952898
Eremopetasidione	163135-94-2	CC1C(CCC2C1(C=C(C(=O)C2)C(=O)C)C)O	CES2	0.048952898
Eremopetasidione	163135-94-2	CC1C(CCC2C1(C=C(C(=O)C2)C(=O)C)C)O	ESR1	0.048952898
Eremopetasidione	163135-94-2	CC1C(CCC2C1(C=C(C(=O)C2)C(=O)C)C)O	SLC6A4	0.048952898
Eremopetasidione	163135-94-2	CC1C(CCC2C1(C=C(C(=O)C2)C(=O)C)C)O	PTPRF	0.048952898
Eremopetasidione	163135-94-2	CC1C(CCC2C1(C=C(C(=O)C2)C(=O)C)C)O	ATP12A	0.048952898
Eremopetasidione	163135-94-2	CC1C(CCC2C1(C=C(C(=O)C2)C(=O)C)C)O	NR1H3	0.048952898
Eremopetasidione	163135-94-2	CC1C(CCC2C1(C=C(C(=O)C2)C(=O)C)C)O	FABP4	0.048952898
Eremopetasidione	163135-94-2	CC1C(CCC2C1(C=C(C(=O)C2)C(=O)C)C)O	FABP3	0.048952898
Eremopetasidione	163135-94-2	CC1C(CCC2C1(C=C(C(=O)C2)C(=O)C)C)O	FABP5	0.048952898
Eremopetasidione	163135-94-2	CC1C(CCC2C1(C=C(C(=O)C2)C(=O)C)C)O	BRD4	0.048952898
Eremopetasidione	163135-94-2	CC1C(CCC2C1(C=C(C(=O)C2)C(=O)C)C)O	BRD2	0.048952898

Eremopetasidione	163135-94-2	CC1C(CCC2C1(C=C(C(=O)C2)C(=O)C)C)O	POLA1	0.048952898
Eremopetasidione	163135-94-2	CC1C(CCC2C1(C=C(C(=O)C2)C(=O)C)C)O	FAAH	0.048952898
Eremopetasidione	163135-94-2	CC1C(CCC2C1(C=C(C(=O)C2)C(=O)C)C)O	PRKCA	0.048952898
Eremopetasidione	163135-94-2	CC1C(CCC2C1(C=C(C(=O)C2)C(=O)C)C)O	CDC25B	0.048952898
Eremopetasidione	163135-94-2	CC1C(CCC2C1(C=C(C(=O)C2)C(=O)C)C)O	CES1	0.048952898
Eremopetasidione	163135-94-2	CC1C(CCC2C1(C=C(C(=O)C2)C(=O)C)C)O	CDC7	0.048952898
Eremopetasidione	163135-94-2	CC1C(CCC2C1(C=C(C(=O)C2)C(=O)C)C)O	SCD	0.048952898
Eremopetasidione	163135-94-2	CC1C(CCC2C1(C=C(C(=O)C2)C(=O)C)C)O	NR1I2	0.048952898
Eremopetasidione	163135-94-2	CC1C(CCC2C1(C=C(C(=O)C2)C(=O)C)C)O	G6PD	0.048952898
Eremopetasidione	163135-94-2	CC1C(CCC2C1(C=C(C(=O)C2)C(=O)C)C)O	HMGCR	0.048952898
Eremopetasidione	163135-94-2	CC1C(CCC2C1(C=C(C(=O)C2)C(=O)C)C)O	LTB4R	0.048952898
Eremopetasidione	163135-94-2	CC1C(CCC2C1(C=C(C(=O)C2)C(=O)C)C)O	JAK1	0.048952898
Eremopetasidione	163135-94-2	CC1C(CCC2C1(C=C(C(=O)C2)C(=O)C)C)O	TAAR1	0.048952898
Eremopetasidione	163135-94-2	CC1C(CCC2C1(C=C(C(=O)C2)C(=O)C)C)O	HSD17B2	0.048952898
Eremopetasidione	163135-94-2	CC1C(CCC2C1(C=C(C(=O)C2)C(=O)C)C)O	CHRNA3	0.048952898
Eremopetasidione	163135-94-2	CC1C(CCC2C1(C=C(C(=O)C2)C(=O)C)C)O	ADAM17	0.048952898
Eremopetasidione	163135-94-2	CC1C(CCC2C1(C=C(C(=O)C2)C(=O)C)C)O	JAK3	0.048952898
Eremopetasidione	163135-94-2	CC1C(CCC2C1(C=C(C(=O)C2)C(=O)C)C)O	JAK2	0.048952898
Eremopetasidione	163135-94-2	CC1C(CCC2C1(C=C(C(=O)C2)C(=O)C)C)O	TYK2	0.048952898
Eremopetasidione	163135-94-2	CC1C(CCC2C1(C=C(C(=O)C2)C(=O)C)C)O	SRD5A2	0.048952898
Eremopetasidione	163135-94-2	CC1C(CCC2C1(C=C(C(=O)C2)C(=O)C)C)O	PREP	0.048952898
Eremopetasidione	163135-94-2	CC1C(CCC2C1(C=C(C(=O)C2)C(=O)C)C)O	PPARG	0.048952898

Eremopetasidione	163135-94-2	CC1C(CCC2C1(C=C(C(=O)C2)C(=O)C)C)O	PPARA	0.048952898
Eremopetasidione	163135-94-2	CC1C(CCC2C1(C=C(C(=O)C2)C(=O)C)C)O	TERT	0.048952898
Eremopetasidione	163135-94-2	CC1C(CCC2C1(C=C(C(=O)C2)C(=O)C)C)O	LRRK2	0.048952898
Eremopetasidione	163135-94-2	CC1C(CCC2C1(C=C(C(=O)C2)C(=O)C)C)O	ADRB2	0.048952898
Eremopetasidione	163135-94-2	CC1C(CCC2C1(C=C(C(=O)C2)C(=O)C)C)O	ADRB1	0.048952898
Eremopetasidione	163135-94-2	CC1C(CCC2C1(C=C(C(=O)C2)C(=O)C)C)O	ADRB3	0.048952898
Eremopetasidione	163135-94-2	CC1C(CCC2C1(C=C(C(=O)C2)C(=O)C)C)O	CA7	0.048952898
Eremopetasidione	163135-94-2	CC1C(CCC2C1(C=C(C(=O)C2)C(=O)C)C)O	CA3	0.048952898
Eremopetasidione	163135-94-2	CC1C(CCC2C1(C=C(C(=O)C2)C(=O)C)C)O	CA6	0.048952898
Eremopetasidione	163135-94-2	CC1C(CCC2C1(C=C(C(=O)C2)C(=O)C)C)O	CA12	0.048952898
Eremopetasidione	163135-94-2	CC1C(CCC2C1(C=C(C(=O)C2)C(=O)C)C)O	CA14	0.048952898
Eremopetasidione	163135-94-2	CC1C(CCC2C1(C=C(C(=O)C2)C(=O)C)C)O	CA4	0.048952898
Eremopetasidione	163135-94-2	CC1C(CCC2C1(C=C(C(=O)C2)C(=O)C)C)O	CA13	0.048952898
Eremopetasidione	163135-94-2	CC1C(CCC2C1(C=C(C(=O)C2)C(=O)C)C)O	CA5B	0.048952898
Eremopetasidione	163135-94-2	CC1C(CCC2C1(C=C(C(=O)C2)C(=O)C)C)O	CREBBP	0.048952898
Eremopetasidione	163135-94-2	CC1C(CCC2C1(C=C(C(=O)C2)C(=O)C)C)O	NOS1	0.048952898
Eremopetasidione	163135-94-2	CC1C(CCC2C1(C=C(C(=O)C2)C(=O)C)C)O	NOS3	0.048952898
Eremopetasidione	163135-94-2	CC1C(CCC2C1(C=C(C(=O)C2)C(=O)C)C)O	CYP51A1	0.048952898
Eremopetasidione	163135-94-2	CC1C(CCC2C1(C=C(C(=O)C2)C(=O)C)C)O	PPARD	0.048952898
Eremopetasidione	163135-94-2	CC1C(CCC2C1(C=C(C(=O)C2)C(=O)C)C)O	CHRM4	0.048952898
Eremopetasidione	163135-94-2	CC1C(CCC2C1(C=C(C(=O)C2)C(=O)C)C)O	CHRM5	0.048952898
Eremopetasidione	163135-94-2	CC1C(CCC2C1(C=C(C(=O)C2)C(=O)C)C)O	CHRM1	0.048952898

Eremopetasidione	163135-94-2	CC1C(CCC2C1(C=C(C(=O)C2)C(=O)C)C)O	MPO	0.048952898
Eremopetasidione	163135-94-2	CC1C(CCC2C1(C=C(C(=O)C2)C(=O)C)C)O	CHRM3	0.048952898
Eremopetasidione	163135-94-2	CC1C(CCC2C1(C=C(C(=O)C2)C(=O)C)C)O	CDK9	0.048952898
Eremopetasidione	163135-94-2	CC1C(CCC2C1(C=C(C(=O)C2)C(=O)C)C)O	FABP1	0.048952898
Eremopetasidione	163135-94-2	CC1C(CCC2C1(C=C(C(=O)C2)C(=O)C)C)O	FKBP1A	0.048952898
Eremopetasidione	163135-94-2	CC1C(CCC2C1(C=C(C(=O)C2)C(=O)C)C)O	HSD17B3	0.048952898
Eremopetasidione	163135-94-2	CC1C(CCC2C1(C=C(C(=O)C2)C(=O)C)C)O	FNTB	0.048952898
Eremopetasidione	163135-94-2	CC1C(CCC2C1(C=C(C(=O)C2)C(=O)C)C)O	CHRNB4	0.048952898
Glucoliquiritin apioside	157226-47-6	C1C(OC2=C(C1=O)C=CC(=C2)OC3C(C(C(C(O3)CO)O)O)O)C4=CC=C(C=C4)OC5C(C(C(C(O5)CO)O)O)OC6C(C(CO6)(CO)O)O	CYP19A1	0.351256342
Glucoliquiritin apioside	157226-47-6	C1C(OC2=C(C1=O)C=CC(=C2)OC3C(C(C(C(O3)CO)O)O)O)C4=CC=C(C=C4)OC5C(C(C(C(O5)CO)O)O)OC6C(C(CO6)(CO)O)O	SRD5A1	0.020178064
Glucoliquiritin apioside	157226-47-6	C1C(OC2=C(C1=O)C=CC(=C2)OC3C(C(C(C(O3)CO)O)O)O)C4=CC=C(C=C4)OC5C(C(C(C(O5)CO)O)O)OC6C(C(CO6)(CO)O)O	SLC5A1	0.020178064
Glucoliquiritin apioside	157226-47-6	C1C(OC2=C(C1=O)C=CC(=C2)OC3C(C(C(C(O3)CO)O)O)O)C4=CC=C(C=C4)OC5C(C(C(C(O5)CO)O)O)OC6C(C(CO6)(CO)O)O	SLC5A4	0.020178064
Glucoliquiritin apioside	157226-47-6	C1C(OC2=C(C1=O)C=CC(=C2)OC3C(C(C(C(O3)CO)O)O)O)C4=CC=C(C=C4)OC5C(C(C(C(O5)CO)O)O)OC6C(C(CO6)(CO)O)O	SLC5A2	0.020178064
Glucoliquiritin apioside	157226-47-6	C1C(OC2=C(C1=O)C=CC(=C2)OC3C(C(C(C(O3)CO)O)O)O)C4=CC=C(C=C4)OC5C(C(C(C(O5)CO)O)O)OC6C(C(CO6)(CO)O)O	SLC28A3	0.020178064
Glucoliquiritin apioside	157226-47-6	C1C(OC2=C(C1=O)C=CC(=C2)OC3C(C(C(C(O3)CO)O)O)O)C4=CC=C(C=C4)OC5C(C(C(C(O5)CO)O)O)OC6C(C(CO6)(CO)O)O	PTGS1	0.020178064
Glucoliquiritin apioside	157226-47-6	C1C(OC2=C(C1=O)C=CC(=C2)OC3C(C(C(C(O3)CO)O)O)O)C4=CC=C(C=C4)OC5C(C(C(C(O5)CO)O)O)OC6C(C(CO6)(CO)O)O	EIF4A1	0.020178064
Glucoliquiritin apioside	157226-47-6	C1C(OC2=C(C1=O)C=CC(=C2)OC3C(C(C(C(O3)CO)O)O)O)C4=CC=C(C=C4)OC5C(C(C(C(O5)CO)O)O)OC6C(C(CO6)(CO)O)O	EPHX2	0.020178064
Glucoliquiritin apioside	157226-	C1C(OC2=C(C1=O)C=CC(=C2)OC3C(C(C(C(O3)CO)O)	TYR	0.020178064

	47-6	O)O)C4=CC=C(C=C4)OC5C(C(C(C(O5)CO)O)O)OC6C(C(CO6)(CO)O)O		
Glucoliquiritin apioside	157226-47-6	C1C(OC2=C(C1=O)C=CC(=C2)OC3C(C(C(C(O3)CO)O)O)O)C4=CC=C(C=C4)OC5C(C(C(C(O5)CO)O)O)OC6C(C(CO6)(CO)O)O	TDP1	0.020178064
Glucoliquiritin apioside	157226-47-6	C1C(OC2=C(C1=O)C=CC(=C2)OC3C(C(C(C(O3)CO)O)O)O)C4=CC=C(C=C4)OC5C(C(C(C(O5)CO)O)O)OC6C(C(CO6)(CO)O)O	FGF1	0.020178064
Glucoliquiritin apioside	157226-47-6	C1C(OC2=C(C1=O)C=CC(=C2)OC3C(C(C(C(O3)CO)O)O)O)C4=CC=C(C=C4)OC5C(C(C(C(O5)CO)O)O)OC6C(C(CO6)(CO)O)O	FGF2	0.020178064
Glucoliquiritin apioside	157226-47-6	C1C(OC2=C(C1=O)C=CC(=C2)OC3C(C(C(C(O3)CO)O)O)O)C4=CC=C(C=C4)OC5C(C(C(C(O5)CO)O)O)OC6C(C(CO6)(CO)O)O	HPSE	0.020178064
Glucoliquiritin apioside	157226-47-6	C1C(OC2=C(C1=O)C=CC(=C2)OC3C(C(C(C(O3)CO)O)O)O)C4=CC=C(C=C4)OC5C(C(C(C(O5)CO)O)O)OC6C(C(CO6)(CO)O)O	MAOB	0.020178064
Glucoliquiritin apioside	157226-47-6	C1C(OC2=C(C1=O)C=CC(=C2)OC3C(C(C(C(O3)CO)O)O)O)C4=CC=C(C=C4)OC5C(C(C(C(O5)CO)O)O)OC6C(C(CO6)(CO)O)O	TAS2R31	0.020178064
Glucoliquiritin apioside	157226-47-6	C1C(OC2=C(C1=O)C=CC(=C2)OC3C(C(C(C(O3)CO)O)O)O)C4=CC=C(C=C4)OC5C(C(C(C(O5)CO)O)O)OC6C(C(CO6)(CO)O)O	ADORA3	0.020178064
Glucoliquiritin apioside	157226-47-6	C1C(OC2=C(C1=O)C=CC(=C2)OC3C(C(C(C(O3)CO)O)O)O)C4=CC=C(C=C4)OC5C(C(C(C(O5)CO)O)O)OC6C(C(CO6)(CO)O)O	PTPN2	0.020178064
Inulobiose	470-58-6	C(C1C(C(C(O1)(CO)OCC2(C(C(C(O2)CO)O)O)O)O)O)O	CDK1	0.309880067
Inulobiose	470-58-6	C(C1C(C(C(O1)(CO)OCC2(C(C(C(O2)CO)O)O)O)O)O)O	VEGFA	0.159936683
Inulobiose	470-58-6	C(C1C(C(C(O1)(CO)OCC2(C(C(C(O2)CO)O)O)O)O)O)O	FGF1	0.159936683
Inulobiose	470-58-6	C(C1C(C(C(O1)(CO)OCC2(C(C(C(O2)CO)O)O)O)O)O)O	HPSE	0.159936683
Inulobiose	470-58-6	C(C1C(C(C(O1)(CO)OCC2(C(C(C(O2)CO)O)O)O)O)O)O	HSP90AA1	0.118277085
Inulobiose	470-58-6	C(C1C(C(C(O1)(CO)OCC2(C(C(C(O2)CO)O)O)O)O)O)O	FGF2	0.118277085
Inulobiose	470-58-6	C(C1C(C(C(O1)(CO)OCC2(C(C(C(O2)CO)O)O)O)O)O)O	LGALS4	0.10994577
Inulobiose	470-58-6	C(C1C(C(C(O1)(CO)OCC2(C(C(C(O2)CO)O)O)O)O)O)O	LGALS3	0.10994577
Inulobiose	470-58-6	C(C1C(C(C(O1)(CO)OCC2(C(C(C(O2)CO)O)O)O)O)O)O	LGALS8	0.10994577
Inulobiose	470-58-6	C(C1C(C(C(O1)(CO)OCC2(C(C(C(O2)CO)O)O)O)O)O)O	PSEN2	0.101613855
Inulobiose	470-58-6	C(C1C(C(C(O1)(CO)OCC2(C(C(C(O2)CO)O)O)O)O)O)O	HTR2B	0.101613855
Inulobiose	470-58-6	C(C1C(C(C(O1)(CO)OCC2(C(C(C(O2)CO)O)O)O)O)O)O	ADRA2A	0.101613855
Inulobiose	470-58-6	C(C1C(C(C(O1)(CO)OCC2(C(C(C(O2)CO)O)O)O)O)O)O	ADRA2C	0.101613855
Inulobiose	470-58-6	C(C1C(C(C(O1)(CO)OCC2(C(C(C(O2)CO)O)O)O)O)O)O	ADRA2B	0.101613855
Inulobiose	470-58-6	C(C1C(C(C(O1)(CO)OCC2(C(C(C(O2)CO)O)O)O)O)O)O	DRD1	0.101613855
Inulobiose	470-58-6	C(C1C(C(C(O1)(CO)OCC2(C(C(C(O2)CO)O)O)O)O)O)O	DRD2	0.101613855
Inulobiose	470-58-6	C(C1C(C(C(O1)(CO)OCC2(C(C(C(O2)CO)O)O)O)O)O)O	ADRA1D	0.101613855

Inulobiose	470-58-6	C(C1C(C(C(O1)(CO)OCC2(C(C(C(O2)CO)O)O)O)O)O)O	HTR2A	0.101613855
Inulobiose	470-58-6	C(C1C(C(C(O1)(CO)OCC2(C(C(C(O2)CO)O)O)O)O)O)O	HTR2C	0.101613855
Inulobiose	470-58-6	C(C1C(C(C(O1)(CO)OCC2(C(C(C(O2)CO)O)O)O)O)O)O	DRD3	0.101613855
Inulobiose	470-58-6	C(C1C(C(C(O1)(CO)OCC2(C(C(C(O2)CO)O)O)O)O)O)O	CYP2D6	0.101613855
Inulobiose	470-58-6	C(C1C(C(C(O1)(CO)OCC2(C(C(C(O2)CO)O)O)O)O)O)O	HTR6	0.101613855
Inulobiose	470-58-6	C(C1C(C(C(O1)(CO)OCC2(C(C(C(O2)CO)O)O)O)O)O)O	ADRA1A	0.101613855
Inulobiose	470-58-6	C(C1C(C(C(O1)(CO)OCC2(C(C(C(O2)CO)O)O)O)O)O)O	HTR1B	0.101613855
Inulobiose	470-58-6	C(C1C(C(C(O1)(CO)OCC2(C(C(C(O2)CO)O)O)O)O)O)O	RORC	0.101613855
Inulobiose	470-58-6	C(C1C(C(C(O1)(CO)OCC2(C(C(C(O2)CO)O)O)O)O)O)O	STAT3	0.101613855
Inulobiose	470-58-6	C(C1C(C(C(O1)(CO)OCC2(C(C(C(O2)CO)O)O)O)O)O)O	FOLH1	0.101613855
Inulobiose	470-58-6	C(C1C(C(C(O1)(CO)OCC2(C(C(C(O2)CO)O)O)O)O)O)O	TRPV1	0.101613855
Inulobiose	470-58-6	C(C1C(C(C(O1)(CO)OCC2(C(C(C(O2)CO)O)O)O)O)O)O	PSENEN	0.101613855
Inulobiose	470-58-6	C(C1C(C(C(O1)(CO)OCC2(C(C(C(O2)CO)O)O)O)O)O)O	NCSTN	0.101613855
Inulobiose	470-58-6	C(C1C(C(C(O1)(CO)OCC2(C(C(C(O2)CO)O)O)O)O)O)O	APH1A	0.101613855
Inulobiose	470-58-6	C(C1C(C(C(O1)(CO)OCC2(C(C(C(O2)CO)O)O)O)O)O)O	PSEN1	0.101613855
Inulobiose	470-58-6	C(C1C(C(C(O1)(CO)OCC2(C(C(C(O2)CO)O)O)O)O)O)O	APH1B	0.101613855
Isoliquiritin	7014-39-3	C1=CC(=CC=C1C=CC(=O)C2=C(C=C(C=C2)O)O)OC3C(C(C(C(O3)CO)O)O)O	AKR1B1	1
Isoliquiritin	7014-39-3	C1=CC(=CC=C1C=CC(=O)C2=C(C=C(C=C2)O)O)OC3C(C(C(C(O3)CO)O)O)O	TYR	0.106165761
Isoliquiritin	7014-39-3	C1=CC(=CC=C1C=CC(=O)C2=C(C=C(C=C2)O)O)OC3C(C(C(C(O3)CO)O)O)O	TNF	0.106165761
Naringin 6''-rhamnoside	—	CC1C(C(C(C(O1)OCC2C(C(C(C(O2)OC3=CC(=C4C(=O)CC(OC4=C3)C5=CC=C(C=C5)O)O)OC6C(C(C(C(O6)C)O)O)O)O)O)O)O)O	CYP19A1	0.09832327
Naringin 6''-rhamnoside	—	CC1C(C(C(C(O1)OCC2C(C(C(C(O2)OC3=CC(=C4C(=O)CC(OC4=C3)C5=CC=C(C=C5)O)O)OC6C(C(C(C(O6)C)O)O)O)O)O)O)O)O	SRD5A1	0.049845722
Naringin 6''-rhamnoside	—	CC1C(C(C(C(O1)OCC2C(C(C(C(O2)OC3=CC(=C4C(=O)CC(OC4=C3)C5=CC=C(C=C5)O)O)OC6C(C(C(C(O6)C)O)O)O)O)O)O)O)O	SLC5A4	0.049845722
Naringin 6''-rhamnoside	—	CC1C(C(C(C(O1)OCC2C(C(C(C(O2)OC3=CC(=C4C(=O)CC(OC4=C3)C5=CC=C(C=C5)O)O)OC6C(C(C(C(O6)C)O)O)O)O)O)O)O)O	PTGS1	0.049845722
Naringin 6''-rhamnoside	—	CC1C(C(C(C(O1)OCC2C(C(C(C(O2)OC3=CC(=C4C(=O)CC(OC4=C3)C5=CC=C(C=C5)O)O)OC6C(C(C(C(O6)C)O)O)O)O)O)O)O)O	EIF4A1	0.049845722
Naringin 6''-rhamnoside	—	CC1C(C(C(C(O1)OCC2C(C(C(C(O2)OC3=CC(=C4C(=O)CC(OC4=C3)C5=CC=C(C=C5)O)O)OC6C(C(C(C(O6)C)O)O)O)O)O)O)O)O	SLC28A3	0.049845722
Naringin 6''-rhamnoside	—	CC1C(C(C(C(O1)OCC2C(C(C(C(O2)OC3=CC(=C4C(=O)CC(OC4=C3)C5=CC=C(C=C5)O)O)OC6C(C(C(C(O6)C)O)O)O)O)O)O)O)O	ADORA1	0.049845722
Naringin 6''-rhamnoside	—	CC1C(C(C(C(O1)OCC2C(C(C(C(O2)OC3=CC(=C4C(=O)	EPHX2	0.049845722

		CC(OC4=C3)C5=CC=C(C=C5)O)O)OC6C(C(C(C(O6)C)O)O)O)O)O)O)O		
Naringin 6''-rhamnoside	—	CC1C(C(C(C(O1)OCC2C(C(C(C(O2)OC3=CC(=C4C(=O)CC(OC4=C3)C5=CC=C(C=C5)O)O)OC6C(C(C(C(O6)C)O)O)O)O)O)O)O	TYR	0.049845722
Naringin 6''-rhamnoside	—	CC1C(C(C(C(O1)OCC2C(C(C(C(O2)OC3=CC(=C4C(=O)CC(OC4=C3)C5=CC=C(C=C5)O)O)OC6C(C(C(C(O6)C)O)O)O)O)O)O)O	TAS2R31	0.049845722
Naringin 6''-rhamnoside	—	CC1C(C(C(C(O1)OCC2C(C(C(C(O2)OC3=CC(=C4C(=O)CC(OC4=C3)C5=CC=C(C=C5)O)O)OC6C(C(C(C(O6)C)O)O)O)O)O)O)O	TDP1	0.049845722
Naringin 6''-rhamnoside	—	CC1C(C(C(C(O1)OCC2C(C(C(C(O2)OC3=CC(=C4C(=O)CC(OC4=C3)C5=CC=C(C=C5)O)O)OC6C(C(C(C(O6)C)O)O)O)O)O)O)O	SLC5A1	0.049845722
Naringin 6''-rhamnoside	—	CC1C(C(C(C(O1)OCC2C(C(C(C(O2)OC3=CC(=C4C(=O)CC(OC4=C3)C5=CC=C(C=C5)O)O)OC6C(C(C(C(O6)C)O)O)O)O)O)O)O	MAOB	0.049845722
Naringin 6''-rhamnoside	—	CC1C(C(C(C(O1)OCC2C(C(C(C(O2)OC3=CC(=C4C(=O)CC(OC4=C3)C5=CC=C(C=C5)O)O)OC6C(C(C(C(O6)C)O)O)O)O)O)O)O	CYP1B1	0.049845722
Naringin 6''-rhamnoside	—	CC1C(C(C(C(O1)OCC2C(C(C(C(O2)OC3=CC(=C4C(=O)CC(OC4=C3)C5=CC=C(C=C5)O)O)OC6C(C(C(C(O6)C)O)O)O)O)O)O)O	ABCG2	0.049845722
Naringin 6''-rhamnoside	—	CC1C(C(C(C(O1)OCC2C(C(C(C(O2)OC3=CC(=C4C(=O)CC(OC4=C3)C5=CC=C(C=C5)O)O)OC6C(C(C(C(O6)C)O)O)O)O)O)O)O	SLC5A2	0.049845722
Naringin 6''-rhamnoside	—	CC1C(C(C(C(O1)OCC2C(C(C(C(O2)OC3=CC(=C4C(=O)CC(OC4=C3)C5=CC=C(C=C5)O)O)OC6C(C(C(C(O6)C)O)O)O)O)O)O)O	ABCC1	0.049845722
Naringin 6''-rhamnoside	—	CC1C(C(C(C(O1)OCC2C(C(C(C(O2)OC3=CC(=C4C(=O)CC(OC4=C3)C5=CC=C(C=C5)O)O)OC6C(C(C(C(O6)C)O)O)O)O)O)O)O	HSD17B1	0.049845722
Naringin 6''-rhamnoside	—	CC1C(C(C(C(O1)OCC2C(C(C(C(O2)OC3=CC(=C4C(=O)CC(OC4=C3)C5=CC=C(C=C5)O)O)OC6C(C(C(C(O6)C)O)O)O)O)O)O)O	SHBG	0.049845722
Naringin 6''-rhamnoside	—	CC1C(C(C(C(O1)OCC2C(C(C(C(O2)OC3=CC(=C4C(=O)CC(OC4=C3)C5=CC=C(C=C5)O)O)OC6C(C(C(C(O6)C)O)O)O)O)O)O)O	CBR1	0.049845722
Naringin 6''-rhamnoside	—	CC1C(C(C(C(O1)OCC2C(C(C(C(O2)OC3=CC(=C4C(=O)CC(OC4=C3)C5=CC=C(C=C5)O)O)OC6C(C(C(C(O6)C)O)O)O)O)O)O)O	AMY2A	0.049845722
Neolicuroside	120926-46-7	C1C(C(C(O1)OC2C(C(C(OC2OC3=CC=C(C=C3)C=CC(=O)C4=C(C=C(C=C4)O)O)CO)O)O)O)(CO)O	AKR1B1	0.235611249
Neolicuroside	120926-	C1C(C(C(O1)OC2C(C(C(OC2OC3=CC=C(C=C3)C=CC(=	TYR	0.095623787

46-7	O)C4=C(C=C(C(=C4)O)O)CO)O)O)O)(CO)O

Supplementary Table2

KEGG_class	Pathway	out	All	Pvalue	Qvalue	Pathway ID	Genes
Environmental Information Processing	Neuroactive ligand-receptor interaction	74	293	2.79E-36	7.87E-34	ko04080	ENSG00000011677;ENSG00000022355;ENSG00000043591;ENSG00000050628;ENSG00000080644;ENSG00000089041;ENSG00000100300;ENSG00000101180;ENSG00000101204;ENSG00000102239;ENSG00000102468;ENSG00000113327;ENSG00000113580;ENSG00000115353;ENSG00000116329;ENSG00000117971;ENSG00000118432;ENSG00000120907;ENSG00000121764;ENSG00000122194;ENSG00000122420;ENSG00000125384;ENSG00000128271;ENSG00000133019;ENSG00000135312;ENSG00000135914;ENSG00000136160;ENSG00000136928;ENSG00000137252;ENSG00000144891;ENSG00000145864;ENSG00000146399;ENSG00000147246;ENSG00000148680;ENSG00000149295;ENSG00000150594;ENSG00000151577;ENSG00000151617;ENSG00000151834;ENSG00000158748;ENSG00000160716;ENSG00000163485;ENSG00000164082;ENSG00000166206;ENSG00000168412;ENSG00000168539;ENSG00000168959;ENSG00000169252;ENSG00000169403;ENSG00000170214;ENSG00000170425;ENSG00000171522;ENSG00000171873;ENSG00000175344;ENSG00000178394;ENSG00000178623;ENSG00000179546;ENSG00000180210;ENSG00000180720;ENSG00000181072;ENSG00000184160;ENSG00000184845;ENSG00000184984;ENSG00000186297;ENSG00000188778;ENSG00000188822;ENSG00000196639;ENSG00000196689;ENSG00000204681;ENSG00000204983;ENSG00000213903;ENSG00000274286;ENSG00000278195;ENSG00000282608
Environmental Information Processing	Calcium signaling pathway	38	189	3.05E-15	4.30E-13	ko04020	ENSG00000004468;ENSG00000007171;ENSG00000043591;ENSG00000050628;ENSG00000089041;ENSG00000089250;ENSG00000102468;ENSG00000113721;ENSG00000115353;ENSG00000120907;ENSG00000122420;ENSG00000123360;ENSG00000128271;ENSG00000133019;ENSG00000135914;ENSG00000136160;ENSG00000144891;ENSG00000146648;ENSG0

							0000147246;ENSG00000148680;ENSG00000151617;ENSG00000154229;ENSG00000154678;ENSG00000158748;ENSG00000164867;ENSG00000168539;ENSG00000168959;ENSG00000169252;ENSG00000169403;ENSG00000170214;ENSG00000170425;ENSG00000171873;ENSG00000175344;ENSG00000181072;ENSG00000184845;ENSG00000184984;ENSG00000188778;ENSG00000196639
Metabolism	Nitrogen metabolism	12	17	3.12E-13	2.93E-11	ko00910	ENSG00000074410;ENSG00000104267;ENSG00000107159;ENSG00000118298;ENSG00000131686;ENSG00000133742;ENSG00000164879;ENSG00000167434;ENSG00000168748;ENSG00000169239;ENSG00000174990;ENSG00000185015
Environmental Information Processing	cAMP signaling pathway	36	208	1.99E-12	1.40E-10	ko04024	ENSG00000001626;ENSG00000005339;ENSG00000043591;ENSG00000050628;ENSG00000051382;ENSG00000065989;ENSG00000079435;ENSG00000102882;ENSG00000109339;ENSG00000111087;ENSG00000113448;ENSG00000121879;ENSG00000125384;ENSG00000128271;ENSG00000132155;ENSG00000135312;ENSG00000136928;ENSG00000142208;ENSG00000149295;ENSG00000151617;ENSG00000157764;ENSG00000158748;ENSG00000163485;ENSG00000168539;ENSG00000169032;ENSG00000169252;ENSG00000171608;ENSG00000172572;ENSG00000178394;ENSG00000179546;ENSG00000181072;ENSG00000184588;ENSG00000184845;ENSG00000186298;ENSG00000186951;ENSG00000204681
Human Diseases	EGFR tyrosine kinase inhibitor resistance	22	82	6.04E-12	3.40E-10	ko01521	ENSG00000051382;ENSG00000082701;ENSG00000096968;ENSG00000102882;ENSG00000105976;ENSG00000108443;ENSG00000112715;ENSG00000113721;ENSG00000121879;ENSG00000128052;ENSG00000132155;ENSG00000138685;ENSG00000142208;ENSG00000146648;ENSG00000154229;ENSG00000157764;ENSG00000162434;ENSG00000168610;ENSG00000169032;ENSG00000171552;ENSG00000171608;ENSG00000174775
Human Diseases	Pathways in cancer	62	550	9.60E-12	4.51E-10	ko05200	ENSG00000005339;ENSG00000007171;ENSG00000050628;ENSG00000051180;ENSG00000051382;ENSG00000064012;ENSG0000007375

							6;ENSG00000080824;ENSG00000082701;ENSG00000087245;ENSG00000091831;ENSG00000096968;ENSG00000100784;ENSG00000100985;ENSG00000102096;ENSG00000102882;ENSG00000105639;ENSG00000105976;ENSG00000106799;ENSG00000108443;ENSG00000109339;ENSG00000109471;ENSG00000111087;ENSG00000112033;ENSG00000112715;ENSG00000113578;ENSG00000113721;ENSG00000116478;ENSG00000121879;ENSG00000123374;ENSG00000125384;ENSG00000128602;ENSG00000132155;ENSG00000132170;ENSG00000133101;ENSG00000135446;ENSG00000135766;ENSG00000136160;ENSG00000137193;ENSG00000138685;ENSG00000140009;ENSG00000142208;ENSG00000144891;ENSG00000146648;ENSG00000151617;ENSG00000154229;ENSG00000154342;ENSG00000157404;ENSG00000157764;ENSG00000162434;ENSG00000164305;ENSG00000164362;ENSG00000164690;ENSG00000165806;ENSG00000168610;ENSG00000169032;ENSG00000169083;ENSG00000171522;ENSG00000171552;ENSG00000171608;ENSG00000174775;ENSG00000196611
Human Diseases	AGE-RAGE signaling pathway in diabetic complications	25	114	2.74E-11	1.10E-09	ko04933	ENSG00000051382;ENSG00000067606;ENSG00000087245;ENSG00000096968;ENSG00000102882;ENSG00000106799;ENSG00000109339;ENSG00000112062;ENSG00000112715;ENSG00000117525;ENSG00000121879;ENSG00000125538;ENSG00000135446;ENSG00000137193;ENSG00000142208;ENSG00000144891;ENSG00000154229;ENSG00000163932;ENSG00000164305;ENSG00000164867;ENSG00000168610;ENSG00000171608;ENSG00000174775;ENSG00000185386;ENSG00000232810
Human Diseases	Morphine addiction	23	101	7.71E-11	2.72E-09	ko05032	ENSG00000011677;ENSG00000022355;ENSG00000065989;ENSG00000112541;ENSG00000113327;ENSG00000113448;ENSG00000123360;ENSG00000128655;ENSG00000136928;ENSG00000145864;ENSG00000151834;ENSG00000154229;ENSG00000154678;ENSG00000163485;ENSG00000166206;ENSG00000172572;ENSG00000173020;ENSG00000184588;ENSG0

							0000184845;ENSG00000186297;ENSG00000186642;ENSG00000204681;ENSG00000284741
Environmental Information Processing	HIF-1 signaling pathway	23	102	9.55E-11	2.99E-09	ko04066	ENSG00000005339;ENSG00000007171;ENSG00000051382;ENSG00000079277;ENSG00000102755;ENSG00000102882;ENSG00000108443;ENSG00000111640;ENSG00000112715;ENSG00000121879;ENSG00000134333;ENSG00000135766;ENSG00000142208;ENSG00000146648;ENSG00000154229;ENSG00000156515;ENSG00000159399;ENSG00000164867;ENSG00000168610;ENSG00000169032;ENSG00000170525;ENSG00000171105;ENSG00000171608
Organismal Systems	Serotonergic synapse	25	125	2.26E-10	6.38E-09	ko04726	ENSG00000012779;ENSG00000069535;ENSG00000073756;ENSG00000095303;ENSG00000100197;ENSG00000102468;ENSG00000102882;ENSG00000108576;ENSG00000132155;ENSG00000135312;ENSG00000135914;ENSG00000142192;ENSG00000145864;ENSG00000147246;ENSG00000148680;ENSG00000154229;ENSG00000157764;ENSG00000158748;ENSG00000164305;ENSG00000166206;ENSG00000169032;ENSG00000174775;ENSG00000178394;ENSG00000179546;ENSG00000189221
Human Diseases	MicroRNAs in cancer	29	168	3.30E-10	8.46E-09	ko05206	ENSG00000005339;ENSG00000019186;ENSG00000073756;ENSG00000085563;ENSG00000088305;ENSG00000100784;ENSG00000100985;ENSG00000101224;ENSG00000103222;ENSG00000105976;ENSG00000112715;ENSG00000113721;ENSG00000116478;ENSG00000121879;ENSG00000132155;ENSG00000137193;ENSG00000138061;ENSG00000143384;ENSG00000146648;ENSG00000149311;ENSG00000154229;ENSG00000154342;ENSG00000156103;ENSG00000158402;ENSG00000164045;ENSG00000164305;ENSG00000168610;ENSG00000169032;ENSG00000174775
Human Diseases	Acute myeloid leukemia	18	68	6.71E-10	1.58E-08	ko05221	ENSG00000005381;ENSG00000051382;ENSG00000102096;ENSG00000102882;ENSG00000108443;ENSG00000112033;ENSG00000121879;ENSG00000132155;ENSG00000133101;ENSG00000137193;ENSG00000140379;ENSG00000142208;ENSG00000157404;ENSG00000157764;ENSG00000168610;ENSG00000169032;ENSG00000171608;ENSG00000174775

Human Diseases	Insulin resistance	24	122	7.49E-10	1.62E-08	ko04931	ENSG00000025434;ENSG00000051382;ENSG00000067606;ENSG00000068976;ENSG00000071242;ENSG00000082701;ENSG00000100504;ENSG00000108443;ENSG00000109339;ENSG00000110090;ENSG00000121879;ENSG00000142208;ENSG00000142949;ENSG00000163932;ENSG00000164867;ENSG00000168610;ENSG00000171105;ENSG00000171608;ENSG00000179295;ENSG00000186298;ENSG00000186951;ENSG00000196396;ENSG00000198408;ENSG00000232810
Cellular Processes	Apoptosis	27	153	8.26E-10	1.66E-08	ko04210	ENSG00000051382;ENSG00000064012;ENSG00000102882;ENSG00000103811;ENSG00000106144;ENSG00000109339;ENSG00000121879;ENSG00000132155;ENSG00000135047;ENSG00000136943;ENSG00000138794;ENSG00000140379;ENSG00000142208;ENSG00000143384;ENSG00000143387;ENSG00000143799;ENSG00000149311;ENSG00000163131;ENSG00000164305;ENSG00000164733;ENSG00000165806;ENSG00000169032;ENSG00000171552;ENSG00000171608;ENSG00000174775;ENSG00000178607;ENSG00000232810
Human Diseases	Proteoglycans in cancer	32	210	1.06E-09	1.95E-08	ko05205	ENSG00000051382;ENSG00000087245;ENSG00000091831;ENSG00000100985;ENSG00000102882;ENSG00000105976;ENSG00000108443;ENSG00000111679;ENSG00000112062;ENSG00000112715;ENSG00000121879;ENSG00000128052;ENSG00000128602;ENSG00000132155;ENSG00000135047;ENSG00000138685;ENSG00000142208;ENSG00000146648;ENSG00000154229;ENSG00000154342;ENSG00000157764;ENSG00000164305;ENSG00000164690;ENSG00000168610;ENSG00000169032;ENSG00000171608;ENSG00000173083;ENSG00000174775;ENSG00000179295;ENSG00000185386;ENSG00000186298;ENSG00000232810
Organismal Systems	Progesterone-mediated oocyte maturation	22	105	1.11E-09	1.95E-08	ko04914	ENSG00000051382;ENSG00000071242;ENSG00000080824;ENSG00000082175;ENSG00000101224;ENSG00000102882;ENSG00000109339;ENSG00000112062;ENSG00000121879;ENSG00000123374;ENSG00000132155;ENSG00000133101;ENSG00000134057;ENSG00000142208;ENSG00000145386;ENSG00000157764;E

							NSG00000158402;ENSG00000164045;ENSG00000169032;ENSG00000170312;ENSG00000171608;ENSG00000185386
Organismal Systems	Inflammatory mediator regulation of TRP channels	21	101	3.09E-09	5.13E-08	ko04750	ENSG00000027075;ENSG00000051382;ENSG00000102468;ENSG00000104321;ENSG00000109339;ENSG00000112062;ENSG00000121879;ENSG00000125384;ENSG00000125538;ENSG00000135914;ENSG00000144481;ENSG00000147246;ENSG00000154229;ENSG00000163932;ENSG00000171522;ENSG00000171608;ENSG00000184381;ENSG00000185386;ENSG00000186298;ENSG00000196639;ENSG00000196689
Human Diseases	Pancreatic cancer	18	77	5.91E-09	9.27E-08	ko05212	ENSG00000051180;ENSG00000051382;ENSG00000102882;ENSG00000106799;ENSG00000108443;ENSG00000109339;ENSG00000112715;ENSG00000121879;ENSG00000132155;ENSG00000135446;ENSG00000142208;ENSG00000146648;ENSG00000157764;ENSG00000162434;ENSG00000168610;ENSG00000169032;ENSG00000171552;ENSG00000171608
Environmental Information Processing	VEGF signaling pathway	16	61	7.02E-09	1.04E-07	ko04370	ENSG00000051382;ENSG00000073756;ENSG00000102882;ENSG00000112062;ENSG00000112715;ENSG00000121879;ENSG00000128052;ENSG00000132155;ENSG00000142208;ENSG00000154229;ENSG00000162889;ENSG00000164867;ENSG00000169032;ENSG00000171608;ENSG00000174775;ENSG00000185386
Organismal Systems	Relaxin signaling pathway	24	138	9.96E-09	1.40E-07	ko04926	ENSG00000007171;ENSG00000051382;ENSG00000067606;ENSG00000087245;ENSG00000089250;ENSG00000100985;ENSG00000102882;ENSG00000106799;ENSG00000109339;ENSG00000112062;ENSG00000112715;ENSG00000121879;ENSG00000132155;ENSG00000136160;ENSG00000137745;ENSG00000142208;ENSG00000146648;ENSG00000154229;ENSG00000164867;ENSG00000169032;ENSG00000171608;ENSG00000174775;ENSG00000185386;ENSG00000196611
Organismal Systems	Prolactin signaling pathway	17	74	2.09E-08	2.80E-07	ko04917	ENSG00000051382;ENSG00000082701;ENSG00000091831;ENSG00000096968;ENSG00000102882;ENSG00000109339;ENSG00000112062;ENSG00000121879;ENSG00000132155;ENSG00000140009;ENSG00000142208;ENSG000

							00148795;ENSG00000168610;ENSG00000169032;ENSG00000171608;ENSG00000174775;ENSG00000185386
Human Diseases	Bladder cancer	13	43	2.87E-08	3.68E-07	ko05219	ENSG00000025708;ENSG00000087245;ENSG00000100784;ENSG00000100985;ENSG00000102882;ENSG00000112715;ENSG00000132155;ENSG00000135446;ENSG00000146648;ENSG00000157764;ENSG00000169032;ENSG00000174775;ENSG00000196611
Human Diseases	Central carbon metabolism in cancer	16	69	4.75E-08	5.73E-07	ko05230	ENSG00000051382;ENSG00000102882;ENSG00000105976;ENSG00000113721;ENSG00000121879;ENSG00000132155;ENSG00000134333;ENSG00000142208;ENSG00000146648;ENSG00000156515;ENSG00000157404;ENSG00000159399;ENSG00000160211;ENSG00000169032;ENSG00000171608;ENSG00000174775
Environmental Information Processing	cGMP - PKG signaling pathway	26	172	4.88E-08	5.73E-07	ko04022	ENSG00000043591;ENSG00000102882;ENSG00000116329;ENSG00000120907;ENSG00000132155;ENSG00000136160;ENSG00000138735;ENSG00000142208;ENSG00000144891;ENSG00000150594;ENSG00000151617;ENSG00000156113;ENSG00000163485;ENSG00000164867;ENSG00000169032;ENSG00000169252;ENSG00000170214;ENSG00000171105;ENSG00000171873;ENSG00000172572;ENSG00000184160;ENSG00000186298;ENSG00000186642;ENSG00000188778;ENSG00000274286;ENSG00000282608
Human Diseases	Prostate cancer	19	101	9.73E-08	1.10E-06	ko05215	ENSG00000005339;ENSG00000051382;ENSG00000080824;ENSG00000082701;ENSG00000100985;ENSG00000102882;ENSG00000113721;ENSG00000121879;ENSG00000123374;ENSG00000132155;ENSG00000142208;ENSG00000146648;ENSG00000149968;ENSG00000157764;ENSG00000169032;ENSG00000169083;ENSG00000171608;ENSG00000174775;ENSG00000277893
Organismal Systems	Regulation of lipolysis in adipocyte	14	56	1.21E-07	1.31E-06	ko04923	ENSG00000043591;ENSG00000050628;ENSG00000051382;ENSG00000073756;ENSG00000079435;ENSG00000095303;ENSG00000121879;ENSG00000142208;ENSG00000163485;ENSG00000169252;ENSG00000170323;ENSG00000171105;ENSG00000171608;ENSG00000188778

Human Diseases	Endocrine resistance	20	116	2.01E-07	2.03E-06	ko01522	ENSG00000051382;ENSG00000087245;ENSG00000091831;ENSG00000100197;ENSG00000100985;ENSG00000102882;ENSG00000108443;ENSG00000109339;ENSG00000112062;ENSG00000121879;ENSG00000132155;ENSG00000135446;ENSG00000140009;ENSG00000142208;ENSG00000146648;ENSG00000157764;ENSG00000169032;ENSG00000171608;ENSG00000174775;ENSG00000185386
Organismal Systems	C-type lectin receptor signaling pathway	20	116	2.01E-07	2.03E-06	ko04625	ENSG00000051382;ENSG00000064012;ENSG00000073756;ENSG00000102882;ENSG00000109339;ENSG00000109471;ENSG00000112062;ENSG00000121879;ENSG00000125538;ENSG00000132155;ENSG00000137752;ENSG00000142208;ENSG00000162889;ENSG00000163932;ENSG00000171608;ENSG00000172175;ENSG00000174775;ENSG00000179295;ENSG00000185386;ENSG00000232810
Environmental Information Processing	FoxO signaling pathway	22	139	2.36E-07	2.30E-06	ko04068	ENSG00000005339;ENSG00000051382;ENSG00000102882;ENSG00000106799;ENSG00000109339;ENSG00000112062;ENSG00000121879;ENSG00000123374;ENSG00000132155;ENSG00000134057;ENSG00000142208;ENSG00000146648;ENSG00000149311;ENSG00000157764;ENSG00000168610;ENSG00000169032;ENSG00000171105;ENSG00000171608;ENSG00000174775;ENSG00000185386;ENSG00000213923;ENSG00000283900
Organismal Systems	Insulin signaling pathway	23	153	3.27E-07	3.07E-06	ko04910	ENSG00000051382;ENSG00000067606;ENSG00000068976;ENSG00000079277;ENSG00000079435;ENSG00000082701;ENSG00000100504;ENSG00000102882;ENSG00000108443;ENSG00000109339;ENSG00000121879;ENSG00000132155;ENSG00000142208;ENSG00000142949;ENSG00000156515;ENSG00000157764;ENSG00000159399;ENSG00000169032;ENSG00000171105;ENSG00000171608;ENSG00000174775;ENSG00000186298;ENSG00000196396
Environmental Information Processing	Sphingolipid signaling pathway	21	132	4.08E-07	3.61E-06	ko04071	ENSG00000051382;ENSG00000066027;ENSG00000067606;ENSG00000102882;ENSG00000103222;ENSG00000109339;ENSG00000112062;ENSG00000113575;ENSG00000116329;ENSG00000121879;ENSG00000132155;ENSG00000142208;ENSG00000154229;ENSG00000163

							485;ENSG00000164867;ENSG00000169032;ENSG00000171608;ENSG00000174775;ENSG00000185386;ENSG00000232810;ENSG00000282608
Organismal Systems	Cholinergic synapse	20	121	4.10E-07	3.61E-06	ko04725	ENSG00000051382;ENSG00000080644;ENSG00000096968;ENSG00000101204;ENSG00000102882;ENSG00000115665;ENSG00000117971;ENSG00000121879;ENSG00000133019;ENSG00000142208;ENSG00000154229;ENSG00000160716;ENSG00000168539;ENSG00000169032;ENSG00000171608;ENSG00000174775;ENSG00000175344;ENSG00000180720;ENSG00000181072;ENSG00000184984
Metabolism	Steroid hormone biosynthesis	15	72	5.61E-07	4.79E-06	ko00140	ENSG00000086696;ENSG00000101846;ENSG00000108786;ENSG00000117594;ENSG00000130948;ENSG00000137869;ENSG00000138061;ENSG00000145545;ENSG00000148795;ENSG00000160882;ENSG00000171234;ENSG00000176387;ENSG00000179142;ENSG00000196139;ENSG00000277893
Organismal Systems	Neurotrophin signaling pathway	20	124	6.16E-07	5.11E-06	ko04722	ENSG00000051382;ENSG00000071242;ENSG00000080815;ENSG00000082701;ENSG00000100784;ENSG00000102882;ENSG00000109339;ENSG00000112062;ENSG00000121879;ENSG00000132155;ENSG00000142208;ENSG00000143801;ENSG00000157764;ENSG00000162889;ENSG00000163932;ENSG00000169032;ENSG00000171608;ENSG00000174775;ENSG00000179295;ENSG00000185386
Environmental Information Processing	Rap1 signaling pathway	27	211	8.66E-07	6.97E-06	ko04015	ENSG00000051382;ENSG00000067606;ENSG00000102755;ENSG00000102882;ENSG00000105976;ENSG00000112062;ENSG00000112715;ENSG00000113578;ENSG00000113721;ENSG00000118432;ENSG00000121879;ENSG00000128052;ENSG00000128271;ENSG00000132155;ENSG00000138685;ENSG00000142208;ENSG00000146648;ENSG00000149295;ENSG00000154229;ENSG00000157404;ENSG00000157764;ENSG00000169032;ENSG00000170425;ENSG00000171105;ENSG00000171608;ENSG00000174775;ENSG00000185386
Human Diseases	Melanoma	15	76	1.17E-06	9.15E-06	ko05218	ENSG00000051382;ENSG00000102882;ENSG00000105976;ENSG00000113578;ENSG00000113721;ENSG00000121879;ENSG0000013215

							5;ENSG00000135446;ENSG00000138685;ENSG00000142208;ENSG00000146648;ENSG00000157764;ENSG00000169032;ENSG00000171608;ENSG00000174775
Organismal Systems	Renin secretion	14	67	1.29E-06	9.84E-06	ko04924	ENSG00000043591;ENSG00000123360;ENSG00000125384;ENSG00000144891;ENSG00000151617;ENSG00000154678;ENSG00000156113;ENSG00000159640;ENSG00000163485;ENSG00000164733;ENSG00000169252;ENSG00000171522;ENSG00000172572;ENSG00000188778
Human Diseases	Hepatitis C	21	144	1.78E-06	1.32E-05	ko05160	ENSG00000025434;ENSG00000051382;ENSG00000082701;ENSG00000102882;ENSG00000105397;ENSG00000109339;ENSG00000110651;ENSG00000112062;ENSG00000113575;ENSG00000121879;ENSG00000132155;ENSG00000142208;ENSG00000146648;ENSG00000157764;ENSG00000162434;ENSG00000168610;ENSG00000171608;ENSG00000174775;ENSG00000185386;ENSG00000186951;ENSG00000232810
Human Diseases	Renal cell carcinoma	14	71	2.69E-06	1.95E-05	ko05211	ENSG00000005339;ENSG00000051382;ENSG00000102882;ENSG00000105976;ENSG00000112715;ENSG00000121879;ENSG00000132155;ENSG00000135766;ENSG00000142208;ENSG00000157764;ENSG00000169032;ENSG00000171608;ENSG00000174775;ENSG00000179295
Human Diseases	Hepatitis B	22	160	2.76E-06	1.95E-05	ko05161	ENSG00000005339;ENSG00000051382;ENSG00000064012;ENSG00000100985;ENSG00000102882;ENSG00000106799;ENSG00000109339;ENSG00000121879;ENSG00000123374;ENSG00000132155;ENSG00000133101;ENSG00000135446;ENSG00000142208;ENSG00000145386;ENSG00000154229;ENSG00000162434;ENSG00000164305;ENSG00000168610;ENSG00000169032;ENSG00000171608;ENSG00000174775;ENSG00000232810
Human Diseases	Non-small cell lung cancer	14	74	4.51E-06	3.10E-05	ko05223	ENSG00000051382;ENSG00000102882;ENSG00000105639;ENSG00000121879;ENSG00000132155;ENSG00000135446;ENSG00000142208;ENSG00000146648;ENSG00000154229;ENSG00000157764;ENSG00000168610;ENSG00000169032;ENSG00000171608;ENSG00000174

							775
Environmental Information Processing	AMPK signaling pathway	19	129	4.78E-06	3.21E-05	ko04152	ENSG00000001626;ENSG00000051382;ENSG00000066027;ENSG00000079435;ENSG00000099194;ENSG00000108443;ENSG00000110090;ENSG00000113161;ENSG00000113575;ENSG00000114268;ENSG00000120907;ENSG00000121879;ENSG00000132170;ENSG00000133101;ENSG00000142208;ENSG00000145386;ENSG00000170525;ENSG00000171105;ENSG00000171608
Environmental Information Processing	TNF signaling pathway	19	130	5.37E-06	3.52E-05	ko04668	ENSG00000051382;ENSG00000064012;ENSG00000073756;ENSG00000100784;ENSG00000100985;ENSG00000102882;ENSG00000109339;ENSG00000112062;ENSG00000121879;ENSG00000125538;ENSG00000142208;ENSG00000149968;ENSG00000157227;ENSG00000164305;ENSG00000165806;ENSG00000169032;ENSG00000171608;ENSG00000185386;ENSG00000232810
Cellular Processes	Gap junction	16	98	7.05E-06	4.52E-05	ko04540	ENSG00000043591;ENSG00000102468;ENSG00000102882;ENSG00000113721;ENSG00000132155;ENSG00000135914;ENSG00000141551;ENSG00000146648;ENSG00000147246;ENSG00000149295;ENSG00000154229;ENSG00000168959;ENSG00000169032;ENSG00000170312;ENSG00000174775;ENSG00000184845
Human Diseases	Colorectal cancer	15	88	7.93E-06	4.97E-05	ko05210	ENSG00000051382;ENSG00000082701;ENSG00000102882;ENSG00000106799;ENSG00000108443;ENSG00000109339;ENSG00000121879;ENSG00000132155;ENSG00000142208;ENSG00000146648;ENSG00000157764;ENSG00000164305;ENSG00000169032;ENSG00000171608;ENSG00000174775
Organismal Systems	Carbohydrate digestion and absorption	11	49	8.64E-06	5.30E-05	ko04973	ENSG00000051382;ENSG00000090402;ENSG00000100170;ENSG00000121879;ENSG00000137700;ENSG00000142208;ENSG00000156515;ENSG00000159399;ENSG00000171608;ENSG00000243480;ENSG00000257335
Organismal Systems	Fc epsilon RI signaling	14	79	1.00E-05	5.88E-05	ko04664	ENSG00000012779;ENSG00000051382;ENSG00000102882;ENSG00000109339;ENSG00000112062;ENSG00000121879;ENSG00000132155;ENSG00000142208;ENSG00000154229;ENSG00000169032;ENSG00000171608;ENSG000

	pathway						00174775;ENSG00000185386;ENSG00000232810
Human Diseases	Chronic myeloid leukemia	14	79	1.00E-05	5.88E-05	ko05220	ENSG00000051382;ENSG00000102882;ENSG00000106799;ENSG00000116478;ENSG00000121879;ENSG00000132155;ENSG00000135446;ENSG00000142208;ENSG00000157764;ENSG00000169032;ENSG00000171552;ENSG00000171608;ENSG00000174775;ENSG00000179295
Human Diseases	Gastric cancer	21	161	1.08E-05	6.22E-05	ko05226	ENSG00000051382;ENSG00000082701;ENSG00000085563;ENSG00000102882;ENSG00000105976;ENSG00000106799;ENSG00000108443;ENSG00000113578;ENSG00000121879;ENSG00000123374;ENSG00000132155;ENSG00000138685;ENSG00000142208;ENSG00000146648;ENSG00000154342;ENSG00000157764;ENSG00000164362;ENSG00000164690;ENSG00000169032;ENSG00000171608;ENSG00000174775
Organismal Systems	Vascular smooth muscle contraction	18	125	1.17E-05	6.62E-05	ko04270	ENSG00000027075;ENSG00000102882;ENSG00000120907;ENSG00000128271;ENSG00000132155;ENSG00000144891;ENSG00000151617;ENSG00000154229;ENSG00000156113;ENSG00000157764;ENSG00000163932;ENSG00000169032;ENSG00000170214;ENSG00000170425;ENSG00000170890;ENSG00000171873;ENSG00000184381;ENSG00000186298
Human Diseases	Nicotine addiction	10	42	1.28E-05	7.06E-05	ko05033	ENSG00000011677;ENSG00000022355;ENSG00000101204;ENSG00000113327;ENSG00000145864;ENSG00000151834;ENSG00000160716;ENSG00000166206;ENSG00000175344;ENSG00000186297
Environmental Information Processing	Jak-STAT signaling pathway	21	167	1.91E-05	1.03E-04	ko04630	ENSG00000005339;ENSG00000051382;ENSG00000096968;ENSG00000105397;ENSG00000105639;ENSG00000109471;ENSG00000111679;ENSG00000113721;ENSG00000121879;ENSG00000132155;ENSG00000137193;ENSG00000142208;ENSG00000143384;ENSG00000146648;ENSG00000162434;ENSG00000168610;ENSG00000171552;ENSG00000171608;ENSG00000174775;ENSG00000175354;ENSG00000179295
Environmental Information	Phospholipase D	20	157	2.49E-05	1.32E-04	ko04072	ENSG00000051382;ENSG00000102882;ENSG00000106976;ENSG00000113721;ENSG00000

Processing	signaling pathway						121879;ENSG00000122420;ENSG00000132155;ENSG00000142208;ENSG00000144891;ENSG00000146648;ENSG00000154229;ENSG00000157404;ENSG00000164082;ENSG00000168959;ENSG00000169032;ENSG00000171105;ENSG00000171608;ENSG00000174775;ENSG00000179295;ENSG00000180871
Human Diseases	Glioma	13	75	2.66E-05	1.39E-04	ko05214	ENSG00000051382;ENSG00000102882;ENSG00000113721;ENSG00000121879;ENSG00000132155;ENSG00000135446;ENSG00000142208;ENSG00000146648;ENSG00000154229;ENSG00000157764;ENSG00000169032;ENSG00000171608;ENSG00000174775
Organismal Systems	Aldosterone-regulated sodium reabsorption	9	37	2.90E-05	1.49E-04	ko04960	ENSG00000051382;ENSG00000102882;ENSG00000121879;ENSG00000151623;ENSG00000151704;ENSG00000154229;ENSG00000171105;ENSG00000171608;ENSG00000176387
Human Diseases	Alzheimer disease	23	199	3.07E-05	1.51E-04	ko05010	ENSG00000064012;ENSG00000080815;ENSG00000082701;ENSG00000089250;ENSG00000102882;ENSG00000111640;ENSG00000117362;ENSG00000125538;ENSG00000138613;ENSG00000142192;ENSG00000143801;ENSG00000151694;ENSG00000162736;ENSG00000164305;ENSG00000164885;ENSG00000165806;ENSG00000176749;ENSG00000178607;ENSG00000186318;ENSG00000189043;ENSG00000196549;ENSG00000205155;ENSG00000232810
Environmental Information Processing	PI3K-Akt signaling pathway	35	374	3.12E-05	1.51E-04	ko04151	ENSG00000051382;ENSG00000066027;ENSG00000080824;ENSG00000082701;ENSG00000096968;ENSG00000102755;ENSG00000102882;ENSG00000105639;ENSG00000105976;ENSG00000108443;ENSG00000109471;ENSG00000112715;ENSG00000113575;ENSG00000113578;ENSG00000113721;ENSG00000121879;ENSG00000123374;ENSG00000128052;ENSG00000132155;ENSG00000135446;ENSG00000138685;ENSG00000142208;ENSG00000143384;ENSG00000146648;ENSG00000154229;ENSG00000157404;ENSG00000162434;ENSG00000164867;ENSG00000168539;ENSG00000169032;ENSG00000171105;ENSG00000171552;ENSG00000171608;ENSG00000174775;ENSG0

							0000181072
Environmental Information Processing	ErbB signaling pathway	14	87	3.13E-05	1.51E-04	ko04012	ENSG00000051382;ENSG00000082701;ENSG00000102882;ENSG00000108443;ENSG00000109339;ENSG00000121879;ENSG00000132155;ENSG00000142208;ENSG00000146648;ENSG00000154229;ENSG00000157764;ENSG00000169032;ENSG00000171608;ENSG00000174775
Human Diseases	Kaposi sarcoma-associated herpesvirus infection	26	241	3.17E-05	1.51E-04	ko05167	ENSG00000005339;ENSG00000051382;ENSG00000064012;ENSG00000073756;ENSG00000082701;ENSG00000096968;ENSG00000102882;ENSG00000105397;ENSG00000109339;ENSG00000112062;ENSG00000112715;ENSG00000121879;ENSG00000132155;ENSG00000135446;ENSG00000138685;ENSG00000142208;ENSG00000162434;ENSG00000162889;ENSG00000163823;ENSG00000164305;ENSG00000168610;ENSG00000169032;ENSG00000171608;ENSG00000174775;ENSG00000179934;ENSG00000185386
Human Diseases	Type II diabetes mellitus	11	56	3.32E-05	1.56E-04	ko04930	ENSG00000051382;ENSG00000067606;ENSG00000102882;ENSG00000109339;ENSG00000121879;ENSG00000156515;ENSG00000159399;ENSG00000163932;ENSG00000171105;ENSG00000171608;ENSG00000232810
Environmental Information Processing	MAPK signaling pathway	32	332	3.87E-05	1.79E-04	ko04010	ENSG00000071242;ENSG00000079277;ENSG00000100784;ENSG00000101224;ENSG00000102755;ENSG00000102882;ENSG00000105976;ENSG00000106799;ENSG00000108861;ENSG00000109339;ENSG00000109971;ENSG00000112062;ENSG00000112715;ENSG00000113578;ENSG00000113721;ENSG00000125538;ENSG00000128052;ENSG00000132155;ENSG00000138685;ENSG00000142208;ENSG00000146648;ENSG00000153956;ENSG00000154229;ENSG00000157404;ENSG00000157764;ENSG00000162889;ENSG00000164305;ENSG00000169032;ENSG00000171105;ENSG00000174775;ENSG00000185386;ENSG00000232810
Human Diseases	Breast cancer	20	162	3.94E-05	1.79E-04	ko05224	ENSG00000051382;ENSG00000082175;ENSG00000082701;ENSG00000091831;ENSG00000102882;ENSG00000108443;ENSG00000113578;ENSG00000121879;ENSG00000132155;ENSG00000135446;ENSG00000138685;ENSG000

							00140009;ENSG00000142208;ENSG00000146648;ENSG00000154342;ENSG00000157404;ENSG00000157764;ENSG00000169032;ENSG00000171608;ENSG00000174775
Organismal Systems	PPAR signaling pathway	13	79	4.70E-05	2.10E-04	ko03320	ENSG00000025434;ENSG00000099194;ENSG00000110090;ENSG00000112033;ENSG00000121769;ENSG00000132170;ENSG00000135929;ENSG00000157184;ENSG00000163586;ENSG00000164687;ENSG00000170323;ENSG00000186951;ENSG00000196611
Organismal Systems	Renin-angiotensin system	7	23	4.78E-05	2.11E-04	ko04614	ENSG00000085377;ENSG00000092009;ENSG00000138792;ENSG00000144891;ENSG00000159640;ENSG00000166825;ENSG00000196549
Organismal Systems	T cell receptor signaling pathway	16	114	4.95E-05	2.15E-04	ko04660	ENSG00000051382;ENSG00000082701;ENSG00000102882;ENSG00000109471;ENSG00000111679;ENSG00000112062;ENSG00000121879;ENSG00000132155;ENSG00000135446;ENSG00000142208;ENSG00000169032;ENSG00000171608;ENSG00000172175;ENSG00000174775;ENSG00000185386;ENSG00000232810
Metabolism	Starch and sucrose metabolism	9	40	5.65E-05	2.41E-04	ko00500	ENSG00000068976;ENSG00000090402;ENSG00000100504;ENSG00000118094;ENSG00000156515;ENSG00000159399;ENSG00000171298;ENSG00000243480;ENSG00000257335
Metabolism	Arachidonic acid metabolism	12	70	6.07E-05	2.54E-04	ko00590	ENSG00000012779;ENSG00000059377;ENSG00000073756;ENSG00000095303;ENSG00000111144;ENSG00000120915;ENSG00000148344;ENSG00000159228;ENSG00000163106;ENSG00000170890;ENSG00000184381;ENSG00000196139
Human Diseases	Chagas disease (American trypanosomiasis)	16	116	6.13E-05	2.54E-04	ko05142	ENSG00000007171;ENSG00000051382;ENSG00000064012;ENSG00000102882;ENSG00000106799;ENSG00000109339;ENSG00000109471;ENSG00000112062;ENSG00000113575;ENSG00000121879;ENSG00000125538;ENSG00000142208;ENSG00000159640;ENSG00000171608;ENSG00000185386;ENSG00000232810
Human Diseases	Endometrial cancer	11	61	7.61E-05	3.10E-04	ko05213	ENSG00000051382;ENSG00000082701;ENSG00000102882;ENSG00000121879;ENSG00000132155;ENSG00000142208;ENSG00000146648;ENSG00000157764;ENSG00000169032;ENSG00000171608;ENSG00000174775

Organismal Systems	IL-17 signaling pathway	15	106	7.70E-05	3.10E-04	ko04657	ENSG00000064012;ENSG00000073756;ENSG00000080824;ENSG00000082701;ENSG00000100985;ENSG00000102882;ENSG00000109339;ENSG00000112062;ENSG00000125538;ENSG00000137745;ENSG00000149968;ENSG00000164305;ENSG00000185386;ENSG00000196611;ENSG00000232810
Organismal Systems	Estrogen signaling pathway	20	171	8.54E-05	3.39E-04	ko04915	ENSG00000051382;ENSG00000080824;ENSG00000082175;ENSG00000087245;ENSG00000091831;ENSG00000100985;ENSG00000102882;ENSG00000109971;ENSG00000121879;ENSG00000132155;ENSG00000136928;ENSG00000140009;ENSG00000142208;ENSG00000146648;ENSG00000163932;ENSG00000164867;ENSG00000169032;ENSG00000171608;ENSG00000174775;ENSG00000204681
Environmental Information Processing	Ras signaling pathway	25	243	9.82E-05	3.85E-04	ko04014	ENSG00000051382;ENSG00000102755;ENSG00000102882;ENSG00000105976;ENSG00000109339;ENSG00000112715;ENSG00000113578;ENSG00000113721;ENSG00000121879;ENSG00000128052;ENSG00000132155;ENSG00000138685;ENSG00000142208;ENSG00000146648;ENSG00000148680;ENSG00000154229;ENSG00000157404;ENSG00000169032;ENSG00000170890;ENSG00000171105;ENSG00000171552;ENSG00000171608;ENSG00000174775;ENSG00000179295;ENSG00000184381
Organismal Systems	B cell receptor signaling pathway	12	74	0.000106244	4.10E-04	ko04662	ENSG00000051382;ENSG00000082701;ENSG00000102882;ENSG00000110651;ENSG00000111679;ENSG00000121879;ENSG00000132155;ENSG00000142208;ENSG00000169032;ENSG00000171608;ENSG00000172175;ENSG00000174775
Metabolism	Other glycan degradation	7	27	0.000148052	5.64E-04	ko00511	ENSG00000109323;ENSG00000115488;ENSG00000162139;ENSG00000167280;ENSG00000177628;ENSG00000179163;ENSG00000204099
Human Diseases	Human cytomegalovirus infection	28	307	0.000301778	1.13E-03	ko05163	ENSG00000050628;ENSG00000051382;ENSG00000064012;ENSG00000073756;ENSG00000082701;ENSG00000102882;ENSG00000108443;ENSG00000112062;ENSG00000112715;ENSG00000121879;ENSG00000125384;ENSG00000125538;ENSG00000132155;ENSG00000135446;ENSG00000142208;ENSG00000146648;E

							NSG00000154229;ENSG00000162434;ENSG00000163823;ENSG00000164305;ENSG00000168610;ENSG00000169032;ENSG00000171522;ENSG00000171608;ENSG00000174775;ENSG00000180871;ENSG00000185386;ENSG00000232810
Cellular Processes	Autophagy - animal	16	133	0.000313024	1.16E-03	ko04140	ENSG00000051382;ENSG00000102882;ENSG00000108443;ENSG00000109339;ENSG00000113575;ENSG00000121879;ENSG00000132155;ENSG00000135047;ENSG00000142208;ENSG00000163932;ENSG00000164733;ENSG00000169032;ENSG00000171552;ENSG00000171608;ENSG00000174775;ENSG00000178607
Human Diseases	Choline metabolism in cancer	14	109	0.000374888	1.37E-03	ko05231	ENSG00000051382;ENSG00000102882;ENSG00000108443;ENSG00000109339;ENSG00000113721;ENSG00000115665;ENSG00000121879;ENSG00000132155;ENSG00000142208;ENSG00000146648;ENSG00000154229;ENSG00000169032;ENSG00000171608;ENSG00000174775
Human Diseases	Fluid shear stress and atherosclerosis	17	149	0.000386583	1.40E-03	ko05418	ENSG00000051382;ENSG00000067606;ENSG00000080824;ENSG00000087245;ENSG00000100985;ENSG00000109339;ENSG00000112062;ENSG00000112715;ENSG00000121879;ENSG00000125538;ENSG00000128052;ENSG00000135047;ENSG00000142208;ENSG00000164867;ENSG00000171608;ENSG00000185386;ENSG00000232810
Organismal Systems	Ovarian Steroidogenesis	9	51	0.000404521	1.44E-03	ko04913	ENSG00000012779;ENSG00000073756;ENSG00000086696;ENSG00000108786;ENSG00000137869;ENSG00000138061;ENSG00000148795;ENSG00000171105;ENSG00000196139
Cellular Processes	Signaling pathways regulating pluripotency of stem cells	17	150	0.000418331	1.47E-03	ko04550	ENSG00000051382;ENSG00000082701;ENSG00000096968;ENSG00000102882;ENSG00000105639;ENSG00000112062;ENSG00000121879;ENSG00000132155;ENSG00000138685;ENSG00000142208;ENSG00000154342;ENSG00000162434;ENSG00000168610;ENSG00000169032;ENSG00000171608;ENSG00000174775;ENSG00000185386
Human Diseases	Hepatocellular	19	178	0.00042085	1.47E-03	ko05225	ENSG00000051382;ENSG00000082701;ENSG00000102882;ENSG00000105976;ENSG00000

	carcinoma			4			106799;ENSG00000108443;ENSG00000121879;ENSG00000132155;ENSG00000135446;ENSG00000142208;ENSG00000146648;ENSG00000154229;ENSG00000154342;ENSG00000157764;ENSG00000164362;ENSG00000169032;ENSG00000171552;ENSG00000171608;ENSG00000174775
Human Diseases	Human papillomavirus infection	34	416	0.000549678	1.89E-03	ko05165	ENSG00000005339;ENSG00000051382;ENSG00000064012;ENSG00000066027;ENSG00000067606;ENSG00000073756;ENSG00000080815;ENSG00000082701;ENSG00000094631;ENSG00000102882;ENSG00000105397;ENSG00000108443;ENSG00000112715;ENSG00000113575;ENSG00000113721;ENSG00000116478;ENSG00000121879;ENSG00000123374;ENSG00000132155;ENSG00000133101;ENSG00000135446;ENSG00000142208;ENSG00000145386;ENSG00000146648;ENSG00000149311;ENSG00000154342;ENSG00000162434;ENSG00000164305;ENSG00000164362;ENSG00000169032;ENSG00000171522;ENSG00000171608;ENSG00000174775;ENSG00000232810
Human Diseases	Measles	17	155	0.000613109	2.08E-03	ko05162	ENSG00000051382;ENSG00000082701;ENSG00000096968;ENSG00000101266;ENSG00000105397;ENSG00000105639;ENSG00000109471;ENSG00000109971;ENSG00000115353;ENSG00000121879;ENSG00000123374;ENSG00000125538;ENSG00000135446;ENSG00000142208;ENSG00000162434;ENSG00000168610;ENSG00000171608
Metabolism	Galactose metabolism	7	34	0.000682123	2.29E-03	ko00052	ENSG00000085662;ENSG00000090402;ENSG00000156515;ENSG00000159399;ENSG00000171298;ENSG00000198074;ENSG00000257335
Cellular Processes	Cell cycle	15	129	0.000688883	2.29E-03	ko04110	ENSG00000005339;ENSG00000082701;ENSG00000097046;ENSG00000101224;ENSG00000116478;ENSG00000123374;ENSG00000133101;ENSG00000134057;ENSG00000135446;ENSG00000145386;ENSG00000149311;ENSG00000158402;ENSG00000164045;ENSG00000170312;ENSG00000253729
Organismal Systems	Parathyroid hormon	14	117	0.000774492	2.54E-03	ko04928	ENSG00000019186;ENSG00000065989;ENSG00000102882;ENSG00000111424;ENSG00000113448;ENSG00000132155;ENSG0000013774

	e synthesis, secretion and action						5;ENSG00000146648;ENSG00000154229;ENSG00000156103;ENSG00000157227;ENSG00000157764;ENSG00000169032;ENSG00000184588
Metabolism	Folate biosynthesis	6	26	0.00087548	2.80E-03	ko00790	ENSG00000085662;ENSG00000137563;ENSG00000159228;ENSG00000162551;ENSG00000196139;ENSG00000198074
Cellular Processes	Adherens junction	11	80	0.000882363	2.80E-03	ko04520	ENSG00000005339;ENSG00000101266;ENSG00000102882;ENSG00000105976;ENSG00000106799;ENSG00000111679;ENSG00000142949;ENSG00000143727;ENSG00000146648;ENSG00000171105;ENSG00000196396
Metabolism	Purine metabolism	19	189	0.000884782	2.80E-03	ko00230	ENSG00000065989;ENSG00000101868;ENSG00000112541;ENSG00000113448;ENSG00000123360;ENSG00000128655;ENSG00000133805;ENSG00000138735;ENSG00000154678;ENSG00000156110;ENSG00000160191;ENSG00000165704;ENSG00000172572;ENSG00000178035;ENSG00000184588;ENSG00000186642;ENSG00000196839;ENSG00000198805;ENSG00000284741
Environmental Information Processing	Notch signaling pathway	9	58	0.001070944	3.33E-03	ko04330	ENSG00000005339;ENSG00000080815;ENSG00000116478;ENSG00000117362;ENSG00000138613;ENSG00000143801;ENSG00000151694;ENSG00000162736;ENSG00000205155
Organismal Systems	Chemokine signaling pathway	20	207	0.001074549	3.33E-03	ko04062	ENSG00000051382;ENSG00000067606;ENSG00000082701;ENSG00000096968;ENSG00000102882;ENSG00000105639;ENSG00000121879;ENSG00000132155;ENSG00000142208;ENSG00000157764;ENSG00000163823;ENSG00000163932;ENSG00000168610;ENSG00000169032;ENSG00000171608;ENSG00000173020;ENSG00000173585;ENSG00000174775;ENSG00000179934;ENSG00000180871
Organismal Systems	Long-term potentiation	10	70	0.001111659	3.41E-03	ko04720	ENSG00000005339;ENSG00000071242;ENSG00000102882;ENSG00000132155;ENSG00000154229;ENSG00000157764;ENSG00000168959;ENSG00000169032;ENSG00000174775;ENSG00000186298
Organismal Systems	GnRH signaling	12	96	0.001234805	3.74E-03	ko04912	ENSG00000087245;ENSG00000102882;ENSG00000109339;ENSG00000112062;ENSG00000132155;ENSG00000146648;ENSG0000015422

	pathway						9;ENSG00000157227;ENSG00000163932;ENSG00000169032;ENSG00000174775;ENSG00000185386
Environmental Information Processing	Hedgehog signaling pathway	8	48	0.001250527	3.75E-03	ko04340	ENSG00000082701;ENSG00000111087;ENSG00000128602;ENSG00000141551;ENSG00000164690;ENSG00000173020;ENSG00000213923;ENSG00000283900
Organismal Systems	Retrograde endocannabinoid signaling	17	168	0.001516167	4.50E-03	ko04723	ENSG00000011677;ENSG00000022355;ENSG00000073756;ENSG00000102882;ENSG00000109339;ENSG00000112062;ENSG00000113327;ENSG00000117480;ENSG00000118432;ENSG00000145864;ENSG00000151834;ENSG00000154229;ENSG00000166206;ENSG00000168959;ENSG00000185386;ENSG00000186297;ENSG00000189043
Organismal Systems	Adrenergic signaling in cardiomyocytes	16	154	0.001570555	4.61E-03	ko04261	ENSG00000043591;ENSG00000066027;ENSG00000100784;ENSG00000102882;ENSG00000112062;ENSG00000113575;ENSG00000120907;ENSG00000142208;ENSG00000144891;ENSG00000153956;ENSG00000154229;ENSG00000169252;ENSG00000170214;ENSG00000171873;ENSG00000185386;ENSG00000186298
Organismal Systems	Dopaminergic synapse	15	142	0.001848004	5.37E-03	ko04728	ENSG00000066027;ENSG00000069535;ENSG00000082701;ENSG00000109339;ENSG00000112062;ENSG00000113575;ENSG00000142208;ENSG00000142319;ENSG00000149295;ENSG00000151577;ENSG00000154229;ENSG00000184845;ENSG00000185386;ENSG00000186298;ENSG00000189221
Organismal Systems	Taste transduction	13	117	0.002331669	6.71E-03	ko04742	ENSG00000011677;ENSG00000022355;ENSG00000123360;ENSG00000133019;ENSG00000135312;ENSG00000136928;ENSG00000151834;ENSG00000154678;ENSG00000178394;ENSG00000179546;ENSG00000186297;ENSG00000204681;ENSG00000256436
Human Diseases	Cocaine addiction	8	53	0.002412631	6.87E-03	ko05030	ENSG00000069535;ENSG00000142319;ENSG00000149295;ENSG00000164082;ENSG00000164885;ENSG00000176749;ENSG00000184845;ENSG00000189221
Cellular Processes	Focal adhesion	19	212	0.003361369	9.43E-03	ko04510	ENSG00000051382;ENSG00000082701;ENSG00000102755;ENSG00000102882;ENSG00000105976;ENSG00000109339;ENSG00000112715;ENSG00000113721;ENSG00000121879;ENS

							G00000128052;ENSG00000132155;ENSG00000142208;ENSG00000146648;ENSG00000154229;ENSG00000157764;ENSG00000169032;ENSG00000171608;ENSG00000174775;ENSG00000186298
Organismal Systems	Toll-like receptor signaling pathway	13	122	0.003376237	9.43E-03	ko04620	ENSG00000051382;ENSG00000064012;ENSG00000102882;ENSG00000109339;ENSG00000112062;ENSG00000121879;ENSG00000125538;ENSG00000142208;ENSG00000143387;ENSG00000169032;ENSG00000171608;ENSG00000185386;ENSG00000232810
Organismal Systems	Fc gamma R-mediated phagocytosis	11	95	0.003592156	9.93E-03	ko04666	ENSG00000051382;ENSG00000102882;ENSG00000108443;ENSG00000121879;ENSG00000132155;ENSG00000142208;ENSG00000154229;ENSG00000163932;ENSG00000169032;ENSG00000171608;ENSG00000184381
Metabolism	Fructose and mannose metabolism	6	34	0.003768051	1.03E-02	ko00051	ENSG00000085662;ENSG00000114268;ENSG00000156515;ENSG00000159399;ENSG00000170525;ENSG00000198074
Cellular Processes	Regulation of actin cytoskeleton	19	218	0.004564889	1.24E-02	ko04810	ENSG00000051382;ENSG00000102882;ENSG00000113578;ENSG00000113721;ENSG00000121879;ENSG00000132155;ENSG00000133019;ENSG00000138685;ENSG00000146648;ENSG00000157764;ENSG00000168539;ENSG00000169032;ENSG00000171608;ENSG00000174775;ENSG00000180210;ENSG00000180720;ENSG00000181072;ENSG00000184984;ENSG00000186298
Environmental Information Processing	mTOR signaling pathway	16	172	0.004797759	1.29E-02	ko04150	ENSG00000051382;ENSG00000071242;ENSG00000082701;ENSG00000102882;ENSG00000108443;ENSG00000121879;ENSG00000132155;ENSG00000142208;ENSG00000154229;ENSG00000154342;ENSG00000157764;ENSG00000169032;ENSG00000171105;ENSG00000171608;ENSG00000174775;ENSG00000232810
Organismal Systems	Long-term depression	8	60	0.005293231	1.40E-02	ko04730	ENSG00000089250;ENSG00000102882;ENSG00000113575;ENSG00000132155;ENSG00000154229;ENSG00000157764;ENSG00000169032;ENSG00000174775
Organismal	Salivary	11	100	0.005	1.40E-0	ko04970	ENSG00000004468;ENSG00000043591;ENSG

Systems	secretion			330415	2		00000089250;ENSG00000120907;ENSG00000133019;ENSG00000154229;ENSG00000156113;ENSG00000169252;ENSG00000170214;ENSG00000171873;ENSG00000188778
Cellular Processes	p53 signaling pathway	9	73	0.005384697	1.41E-02	ko04115	ENSG00000064012;ENSG00000123374;ENSG00000134057;ENSG00000135446;ENSG00000146674;ENSG00000149311;ENSG00000164305;ENSG00000170312;ENSG00000171552
Organismal Systems	Pancreatic secretion	11	102	0.00618833	1.59E-02	ko04972	ENSG00000001626;ENSG00000004468;ENSG00000080618;ENSG00000104267;ENSG00000133019;ENSG00000154229;ENSG00000156113;ENSG00000168925;ENSG00000170890;ENSG00000204983;ENSG00000243480
Organismal Systems	Thyroid hormone signaling pathway	13	131	0.006197152	1.59E-02	ko04919	ENSG00000005339;ENSG00000051382;ENSG00000082701;ENSG00000091831;ENSG00000102882;ENSG00000116478;ENSG00000121879;ENSG00000132155;ENSG00000142208;ENSG00000154229;ENSG00000169032;ENSG00000171608;ENSG00000174775
Human Diseases	Non-alcoholic fatty liver disease (NAFLD)	16	178	0.00665962	1.69E-02	ko04932	ENSG00000025434;ENSG00000051382;ENSG00000064012;ENSG00000082701;ENSG00000109339;ENSG00000121879;ENSG00000125538;ENSG00000142208;ENSG00000164305;ENSG00000165806;ENSG00000171105;ENSG00000171608;ENSG00000178607;ENSG00000186951;ENSG00000189043;ENSG00000232810
Human Diseases	Platinum drug resistance	9	76	0.007019379	1.77E-02	ko01524	ENSG00000051382;ENSG00000064012;ENSG00000102882;ENSG00000121879;ENSG00000142208;ENSG00000149311;ENSG00000164305;ENSG00000171552;ENSG00000171608
Human Diseases	Amyotrophic lateral sclerosis (ALS)	8	63	0.00712587	1.77E-02	ko05014	ENSG00000089250;ENSG00000110436;ENSG00000112062;ENSG00000137752;ENSG00000164305;ENSG00000171552;ENSG00000185386;ENSG00000232810
Human Diseases	Influenza A	22	277	0.007150806	1.77E-02	ko05164	ENSG00000005339;ENSG00000051382;ENSG00000082701;ENSG00000096968;ENSG00000102882;ENSG00000105397;ENSG00000109339;ENSG00000109971;ENSG00000112062;ENSG00000121879;ENSG00000122194;ENSG00000125538;ENSG00000132155;ENSG00000137752;ENSG00000142208;ENSG00000154229;ENSG00000162434;ENSG00000169032;ENSG00000171608;ENSG00000185386;ENSG000002

							04983;ENSG00000232810
Metabolism	Arginine and proline metabolism	7	51	0.007594762	1.85E-02	ko00330	ENSG00000007171;ENSG00000065154;ENSG00000069535;ENSG00000089250;ENSG00000123505;ENSG00000164867;ENSG00000189221
Organismal Systems	Melanogenesis	11	105	0.007674541	1.85E-02	ko04916	ENSG00000005339;ENSG00000077498;ENSG00000082701;ENSG00000102882;ENSG00000132155;ENSG00000136160;ENSG00000154229;ENSG00000154342;ENSG00000157404;ENSG00000169032;ENSG00000174775
Human Diseases	Viral carcinogenesis	21	262	0.00767901	1.85E-02	ko05203	ENSG00000005339;ENSG00000051382;ENSG00000064012;ENSG00000070501;ENSG00000094631;ENSG00000102882;ENSG00000105639;ENSG00000116478;ENSG00000121879;ENSG00000123374;ENSG00000133101;ENSG00000135446;ENSG00000145386;ENSG00000162434;ENSG00000162889;ENSG00000164305;ENSG00000168610;ENSG00000170312;ENSG00000171608;ENSG00000174775;ENSG00000179934
Organismal Systems	Platelet activation	13	135	0.007941009	1.90E-02	ko04611	ENSG00000051382;ENSG00000059377;ENSG00000067606;ENSG00000095303;ENSG00000102882;ENSG00000112062;ENSG00000121879;ENSG00000142208;ENSG00000164867;ENSG00000169313;ENSG00000171608;ENSG00000185386;ENSG00000186298
Human Diseases	Epithelial cell signaling in Helicobacter pylori infection	9	78	0.00830393	1.97E-02	ko05120	ENSG00000105976;ENSG00000109339;ENSG00000112062;ENSG00000146648;ENSG00000151694;ENSG00000164305;ENSG00000179295;ENSG00000180871;ENSG00000185386
Human Diseases	Pertussis	10	92	0.008392901	1.97E-02	ko05133	ENSG00000007171;ENSG00000102882;ENSG00000109339;ENSG00000112062;ENSG00000125538;ENSG00000137752;ENSG00000164305;ENSG00000165806;ENSG00000185386;ENSG00000232810
Human Diseases	Antifolate resistanc	6	40	0.008565226	2.00E-02	ko01523	ENSG00000103222;ENSG00000118777;ENSG00000125538;ENSG00000137563;ENSG00000176890;ENSG00000232810

Organismal Systems	e Longevity regulating pathway - multiple species	9	79	0.00900962 2	2.08E-02	ko04213	ENSG00000051382;ENSG00000108443;ENSG00000109971;ENSG00000116478;ENSG00000121879;ENSG00000142208;ENSG00000171105;ENSG00000171608;ENSG00000174775
Organismal Systems	Osteoclast differentiation	16	187	0.01050403	2.41E-02	ko04380	ENSG00000051382;ENSG00000102882;ENSG00000105397;ENSG00000106799;ENSG00000109339;ENSG00000112062;ENSG00000121879;ENSG00000125538;ENSG00000132170;ENSG00000142208;ENSG00000143387;ENSG00000162434;ENSG00000169032;ENSG00000171608;ENSG00000185386;ENSG00000232810
Human Diseases	Alcoholism	16	189	0.0115596	2.63E-02	ko05034	ENSG00000069535;ENSG00000094631;ENSG00000102882;ENSG00000112759;ENSG00000116478;ENSG00000128271;ENSG00000132155;ENSG00000142319;ENSG00000149295;ENSG00000157764;ENSG00000169032;ENSG00000170425;ENSG00000174775;ENSG00000184845;ENSG00000186298;ENSG00000189221
Organismal Systems	Circadian rhythm	5	31	0.01184911	2.67E-02	ko04710	ENSG00000069667;ENSG00000141551;ENSG00000143365;ENSG00000213923;ENSG00000283900
Metabolism	Drug metabolism - other enzymes	9	84	0.01323939	2.96E-02	ko00983	ENSG00000005381;ENSG00000025708;ENSG00000158825;ENSG00000165704;ENSG00000167900;ENSG00000171234;ENSG00000172831;ENSG00000178035;ENSG00000198848
Organismal Systems	GABAergic synapse	10	99	0.01373807	3.05E-02	ko04727	ENSG00000011677;ENSG00000022355;ENSG00000113327;ENSG00000136928;ENSG00000145864;ENSG00000151834;ENSG00000154229;ENSG00000166206;ENSG00000186297;ENSG00000204681
Organismal Systems	NOD-like receptor signaling pathway	16	193	0.01392047	3.07E-02	ko04621	ENSG00000064012;ENSG00000080824;ENSG00000089041;ENSG00000102882;ENSG00000105397;ENSG00000105835;ENSG00000109339;ENSG00000112062;ENSG00000125538;ENSG00000137752;ENSG00000162434;ENSG00000163932;ENSG00000164733;ENSG00000171552;ENSG00000185386;ENSG00000232810
Human	Small	10	100	0.014	3.21E-0	ko05222	ENSG00000007171;ENSG00000051382;ENSG

Diseases	cell lung cancer			67225	2		00000073756;ENSG00000121879;ENSG00000123374;ENSG00000135446;ENSG00000142208;ENSG00000164305;ENSG00000171552;ENSG00000171608
Cellular Processes	Oocyte meiosis	12	131	0.01528157	3.31E-02	ko04114	ENSG00000066027;ENSG00000071242;ENSG00000082175;ENSG00000102882;ENSG00000113575;ENSG00000123374;ENSG00000134057;ENSG00000158402;ENSG00000169032;ENSG00000169083;ENSG00000170312;ENSG00000186298
Cellular Processes	Apoptosis - multiple species	5	33	0.01537754	3.31E-02	ko04215	ENSG00000064012;ENSG00000109339;ENSG00000164305;ENSG00000165806;ENSG00000171552
Metabolism	Neomycin, kanamycin and gentamicin biosynthesis	2	5	0.01862291	3.98E-02	ko00524	ENSG00000156515;ENSG00000159399
Organismal Systems	Bile secretion	8	75	0.01946942	4.13E-02	ko04976	ENSG00000001626;ENSG00000012504;ENSG00000085563;ENSG00000100170;ENSG00000104267;ENSG00000113161;ENSG00000118777;ENSG00000143819
Human Diseases	Chemical carcinogenesis	9	90	0.02007761	4.23E-02	ko05204	ENSG00000073756;ENSG00000108602;ENSG00000117594;ENSG00000138061;ENSG00000143819;ENSG00000159228;ENSG00000163106;ENSG00000171234;ENSG00000175344
Organismal Systems	Th17 cell differentiation	15	186	0.02122267	4.43E-02	ko04659	ENSG00000069667;ENSG00000080824;ENSG00000096968;ENSG00000102882;ENSG00000105397;ENSG00000105639;ENSG00000106799;ENSG00000109339;ENSG00000109471;ENSG00000112062;ENSG00000125538;ENSG00000143365;ENSG00000162434;ENSG00000168610;ENSG00000185386
Cellular Processes	Cellular senescence	23	328	0.02399554	4.98E-02	ko04218	ENSG00000051382;ENSG00000102882;ENSG00000106799;ENSG00000112062;ENSG00000113456;ENSG00000121879;ENSG00000123374;ENSG00000132155;ENSG00000133101;ENSG00000134057;ENSG00000135446;ENSG00000142208;ENSG00000145386;ENSG00000146674;ENSG00000149311;ENSG00000162889;E

NSG00000164045;ENSG00000169032;ENSG00000170312;ENSG00000171608;ENSG00000174775;ENSG00000185386;ENSG00000186298

Supplementary Table3

name	Description	Betweenness Centrality	Closeness Centrality	Degree
Inulobiose	comound	0.00434041	0.31404959	3
CREBBP	target	0.0158264	0.37254902	2
TYK2	target	0.010209	0.37254902	2
JAK2	target	0.010209	0.37254902	2
JAK3	target	0.010209	0.37254902	2
JAK1	target	0.010209	0.37254902	2
RORA	target	0.010209	0.37254902	2
MAPK3	target	0.09922822	0.46341463	3
Eremopetasidione	comound	0.20728131	0.41758242	10
TGFBR1	target	0.02560455	0.33333333	2
IL1B	target	0.02560455	0.33333333	2
Carvylpropionate	comound	7.11E-04	0.25503356	2
PRKCA	target	0.06845798	0.38	3
3_O_Caffeoyl_1_O_methylquinicacid	comound	0	0.27737226	1
PIK3CA	target	0.01453678	0.35185185	2
MAPK11	target	0.0137547	0.35185185	2
RAF1	target	0.01453678	0.35185185	2
2_Dodecylbenzenesulfonicacid	comound	0.07905023	0.36893204	6
Sterol	comound	0	0.28148148	1
MAP2K1	target	0.02933351	0.36538462	2
MAPK14	target	0.02320451	0.36538462	3
RORC	target	0.05132536	0.39583333	4
STAT3	target	0.0138141	0.34545455	3
HSP90AA1	target	0.0416347	0.37254902	4
PerillosideC	comound	0.05861554	0.38383838	6
ESR1	target	0.09941723	0.3877551	4
AKT1	target	0.0054147	0.32758621	2
GSK3B	target	0.0054147	0.32758621	2
PIK3CD	target	0.0054147	0.32758621	2
PIK3CB	target	0.0054147	0.32758621	2
Moclobemide	comound	0.01705615	0.2992126	5
MAPK10	target	0.01486148	0.34545455	2
HDAC1	target	0.0368812	0.37254902	3
CorchoionosideB	comound	0.03591825	0.35514019	4
HRAS	target	0.04179788	0.34545455	3
IL2	target	0.05850047	0.36538462	4
5_Megastigmen_7_y	comound	0.00759509	0.33628319	2

ne_3_9_diol3_glucos<br>ide				
ko04659	path	0.38700415	0.48101266	15
ko04919	path	0.36885721	0.4691358	13
